# Assessing the Reproducibility of the Structured Abstracts Generated by ChatGPT and Bard Compared to Human-Written Abstracts in the Field of Spine Surgery: Comparative Analysis

**DOI:** 10.2196/52001

**Published:** 2024-06-26

**Authors:** Hong Jin Kim, Jae Hyuk Yang, Dong-Gune Chang, Lawrence G Lenke, Javier Pizones, René Castelein, Kota Watanabe, Per D Trobisch, Gregory M Mundis Jr, Seung Woo Suh, Se-Il Suk

**Affiliations:** 1 Department of Orthopedic Surgery Inje University Sanggye Paik Hospital, College of Medicine Inje University Seoul Republic of Korea; 2 Department of Orthopedic Surgery Korea University Anam Hospital, College of Medicine Korea University Seoul Republic of Korea; 3 Department of Orthopedic Surgery The Daniel and Jane Och Spine Hospital Columbia University New York, NY United States; 4 Department of Orthopedic Surgery Hospital Universitario La Paz Madrid Spain; 5 Department of Orthopedic Surgery University Medical Centre Utrecht Utrecht Netherlands; 6 Department of Orthopedic Surgery Keio University School of Medicine Tokyo Japan; 7 Department of Spine Surgery Eifelklinik St. Brigida Simmerath Germany; 8 Department of Orthopaedic Surgery Scripps Clinic La Jolla, CA United States; 9 Department of Orthopedic Surgery Korea University Guro Hospital, College of Medicine Korea University Seoul Republic of Korea

**Keywords:** artificial intelligence, AI, ChatGPT, Bard, scientific abstract, orthopedic surgery, spine, journal guidelines, plagiarism, ethics, spine surgery, surgery, language model, chatbot, formatting guidelines, abstract

## Abstract

**Background:**

Due to recent advances in artificial intelligence (AI), language model applications can generate logical text output that is difficult to distinguish from human writing. ChatGPT (OpenAI) and Bard (subsequently rebranded as “Gemini”; Google AI) were developed using distinct approaches, but little has been studied about the difference in their capability to generate the abstract. The use of AI to write scientific abstracts in the field of spine surgery is the center of much debate and controversy.

**Objective:**

The objective of this study is to assess the reproducibility of the structured abstracts generated by ChatGPT and Bard compared to human-written abstracts in the field of spine surgery.

**Methods:**

In total, 60 abstracts dealing with spine sections were randomly selected from 7 reputable journals and used as ChatGPT and Bard input statements to generate abstracts based on supplied paper titles. A total of 174 abstracts, divided into human-written abstracts, ChatGPT-generated abstracts, and Bard-generated abstracts, were evaluated for compliance with the structured format of journal guidelines and consistency of content. The likelihood of plagiarism and AI output was assessed using the iThenticate and ZeroGPT programs, respectively. A total of 8 reviewers in the spinal field evaluated 30 randomly extracted abstracts to determine whether they were produced by AI or human authors.

**Results:**

The proportion of abstracts that met journal formatting guidelines was greater among ChatGPT abstracts (34/60, 56.6%) compared with those generated by Bard (6/54, 11.1%; *P*<.001). However, a higher proportion of Bard abstracts (49/54, 90.7%) had word counts that met journal guidelines compared with ChatGPT abstracts (30/60, 50%; *P*<.001). The similarity index was significantly lower among ChatGPT-generated abstracts (20.7%) compared with Bard-generated abstracts (32.1%; *P*<.001). The AI-detection program predicted that 21.7% (13/60) of the human group, 63.3% (38/60) of the ChatGPT group, and 87% (47/54) of the Bard group were possibly generated by AI, with an area under the curve value of 0.863 (*P*<.001). The mean detection rate by human reviewers was 53.8% (SD 11.2%), achieving a sensitivity of 56.3% and a specificity of 48.4%. A total of 56.3% (63/112) of the actual human-written abstracts and 55.9% (62/128) of AI-generated abstracts were recognized as human-written and AI-generated by human reviewers, respectively.

**Conclusions:**

Both ChatGPT and Bard can be used to help write abstracts, but most AI-generated abstracts are currently considered unethical due to high plagiarism and AI-detection rates. ChatGPT-generated abstracts appear to be superior to Bard-generated abstracts in meeting journal formatting guidelines. Because humans are unable to accurately distinguish abstracts written by humans from those produced by AI programs, it is crucial to exercise special caution and examine the ethical boundaries of using AI programs, including ChatGPT and Bard.

## Introduction

Artificial intelligence (AI) language models are being applied to various fields, including medicine and health care [1-3]. Novel and open AI programs make it possible to generate structured text within seconds, accelerating the use of AI and providing valuable insights into clinical research [4,5]. One of the more successful applications of these AI programs is generating high-quality theses and providing answers to questions from the United States Medical Licensing Examination [3,6,7]. However, many concerns have been raised about the scientific value of AI-based tools, with ethical issues and reproducibility at the forefront of public debates [8,9].

AI language models are based on complex neural network transformer models known as large language models (LLMs) [10]. Pretraining with large-sized data is used to predict the optimal next elements of textual input. ChatGPT, released in November 2022 and based on GPT-3 software, is the first popular AI language model application. It generates fluent output and reinforces human feedback [5,10]. However, ChatGPT responses are based on information drawn from the internet before a data cut-off date of September 2021. Bard (subsequently rebranded Gemini), released in March 2023, is a new AI language model developed by Google that is based on the language model for dialogue applications family of LLMs. Unlike ChatGPT, it replies to prompts using real-time information in conjunction with Google Internet searches.

Although many proposals for the use of AI programs in scientific writing have been suggested, few relevant studies have been published. One study attempted to compare scientific abstracts generated by ChatGPT with those gathered from 50 human-written abstracts in the field of medicine [5]. However, no similar comparative analyses have involved more specialized subjects. Furthermore, the differences between ChatGPT and Bard in abstract generation have not yet been studied. This study aimed to evaluate the reproducibility of abstracts generated by ChatGPT and Bard compared with human-written abstracts in the field of spinal surgery.

## Methods

### Journal Selection and Abstract Extraction

To evaluate abstracts in the field of spinal surgery, specialists in spinal surgery with more than 10 years of experience responded to the query: “Please introduce the reputable journals in the area of spinal surgery in orthopedics and neurosurgery.” From the responses, we selected 7 journals: *Spine (Phila pa 1976), The Spine Journal, European Spine Journal, Journal of Neurosurgery: Spine, Global Spine Journal, Neurosurgery*, and *The Journal of Bone and Joint Surgery*. We randomly extracted the 60 papers published by the 7 journals (10 from *Spine (Phila pa 1976)*, 10 from *The Spine Journal*, 10 from *European Spine Journal*, 10 from *Journal of Neurosurgery: Spine*, 10 from *Global Spine Journal*, 5 from *Neurosurgery*, and 5 from *The Journal of Bone and Joint Surgery*) to minimize the likelihood of any prior knowledge of the abstracts by the AI programs ChatGPT (May 24 version; OpenAI) and Bard (experiment version after May 15, 2023; Google AI; considering ChatGPT’s knowledge cut-off of September 2021) and by human reviewers.

### Abstract Generation

The titles from the 60 randomly extracted abstracts were used in prompts presented to the AI programs as follows: “Please write a scientific abstract for the article [title] in the style of [journal] at [link].” However, Bard did not produce abstracts in 6 cases (1 from *Spine (Phila pa 1976)*, 1 from *The Spine Journal*, 1 from *European Spine Journal*, 1 from *Journal of Neurosurgery: Spine*, and 2 from *Global Spine Journal*) in response to this prompt, replying that “I’m just a language model, so I can’t help you with that,” “I can’t assist you with that, as I’m only a language model and don’t have the capacity to understand and respond,” and “I’m not programmed to assist with that.” A total of 114 AI-generated abstracts were presented, 60 from ChatGPT (GPT-3.5) and 54 from Bard (Multimedia Appendix 1). Regarding abstract generation, because ChatGPT was sensitive to changes in prompts, we chose the abstract generated in response to the first prompt. As Bard supplied 3 answers to each prompt, we selected the generated abstract that was most similar to the format specified by the journal guidelines.

### Abstract Evaluation

**Figure 1:**
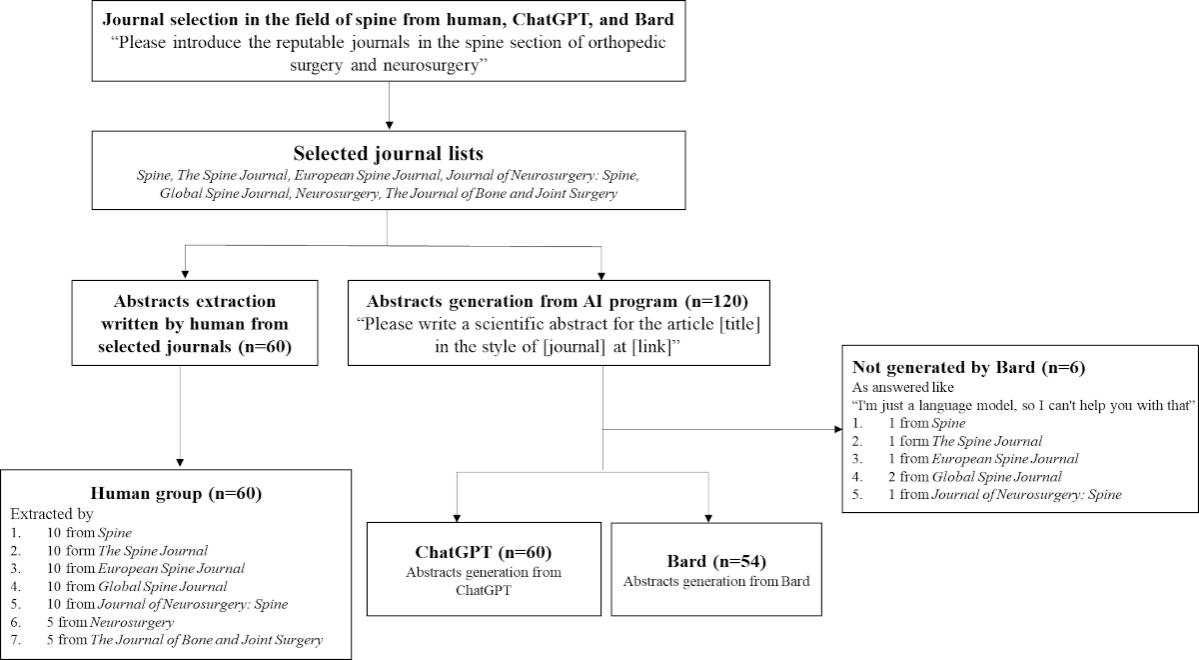
Flowchart of the study. AI: artificial intelligence.

The 60 human-written abstracts and 114 AI-generated abstracts were divided into 3 groups: human (n=60), ChatGPT (n=60), and Bard (n=54; Figure 1). For the AI-generated abstracts, we collected data to assess their reproducibility according to format compliance (binary data: yes or no), total word count, consistency of conclusion (binary data: yes or no), and size of cohort sample. We also assessed the similarity index using iThenticate, which is a widely used program. The similarity index of the human group of abstracts, which were published in journals, was nearly 100%. We compared the similarity indices of the ChatGPT group and the Bard group. Based on popular consensus, plagiarism was considered at a similarity index of 15% or higher. We evaluated the AI detection rate in the 3 groups using ZeroGPT (access date: June 5, 2023), which is a tool designed to detect whether texts are generated by an AI program. ZeroGPT provides both a percentage from 0% (human-written) to 100% (AI and GPT-generated) and 1 of 9 sentences: “Your text is Human written,” “Your text is Most Likely Human written,” “Your text is Most Likely Human written, may include parts generated by AI/GPT,” “Your text is Likely Human written, may include parts generated by AI/GPT,” “Your text contains mixed signals, with some parts generated by AI/GPT,” “Your text is Likely generated by AI/GPT,” “Your text is Most Likely AI/GPT generated,” “Most of Your text is AI/GPT Generated,” and “Your text is AI/GPT Generated.” We evaluated the accuracy of the AI detection using the expression “Your text contains mixed signals, with some parts generated by AI/GPT” for all 3 groups. Last, we evaluated the accuracy of 8 blinded human reviewers in determining how each of the 30 abstracts randomly chosen from the 174 used in the study was written (human- or AI-generated)‚ in the form of binary scores collected by electronic records using Google Forms. None of the blinded human reviewers were provided any information regarding the abstracts until the survey was complete.

### Statistical Analysis and Visualization

Statistical analysis and visualization were performed using R (version 4.3.0; The R Foundation). A normal distribution was confirmed with the Kolmogorov-Smirnov test. After confirming data homogeneity or heteroscedasticity, Student 2-tailed *t* test was used for continuous variables, and the chi-square test was used for categorical variables, as appropriate. A comparison of the 3 groups used a 1-way repeated-measures ANOVA and post hoc analyses using the Bonferroni test. A Pearson correlation analysis was used to assess the correlation of the cohort sample number between human-written and AI-generated abstracts, which were visualized using a heatmap. Receiver operative characteristic (ROC) curve analysis was performed to compare the AI detection rates for human-written and AI-generated abstracts. We calculated the *P* value in the ROC curve based on a null hypothesis with an area under the curve (AUC) of 0.5. The cut-off point in the ROC curve was measured using Youden’s index. Statistical significance was set with a 2-tailed *P*<.05.

### Ethical Considerations

This study was approved by the institutional review board of the Inje University Sanggye Paik Hospital (IRB NON2023-008), and informed written consent was waived from the participants for the electronic survey and publication of this study. The participants who are specialists with more than 10 years of experience in spine surgery were voluntarily recruited without any compensation, and the data from the electronic survey were collected in deidentified status.

## Results

### Similarity Index, AI Detection Rate, and Word Count

The mean similarity indices of the ChatGPT and Bard groups were 20.7% (SD 8.7%) and 32.1% (SD 11%), respectively, exceeding the 15% threshold for the commonly recognized standard of plagiarism (*P*<.001; 95% CI –15.05 to –7.62). The mean AI detection rates achieved by the human, ChatGPT, and Bard groups were 28.4% (SD 25.8%), 60.7% (SD 25%), and 77.7% (SD 21.1%), respectively, with significant differences (*P*<.001). All of the Bonferroni post hoc analyses for AI detection showed statistically significant differences (*P*<.001). For word counts in text, there was a significant difference between the 3 groups (*P*<.001), and the Bonferroni post hoc analysis showed significant differences, except between the human and ChatGPT groups (*P*>.99; Table 1).

**Table 1:** Baseline data in this study.

Variables	Human (n=60), mean (SD)	ChatGPT (n=60), mean (SD)	Bard (n=54), mean (SD)	*P* value	95% CI
Similarity index (%)	100 (0)	20.7 (8.7)	32.1 (11.0)	<.001	–15.05 to –7.62
AI**^a^** detection rate (%)	28.4 (25.8)	60.7 (25.0)	77.7 (21.1)	<.001^b^	N/A^c^
Word count (n)	317.8 (72.4)	317.8 (53.9)	223.8 (44.5)	<.001^d^	N/A

^a^AI: artificial intelligence.

^b^Bonferroni post hoc analysis results for the AI detection rate were as follows: human versus ChatGPT: *P*<.001; 95% CI –42.92 to –21.58; human versus Bard: *P*<.001; 95% CI –60.18 to –38.26; and ChatGPT versus Bard: *P*=.001; 95% CI –27.93 to –6.01.

^c^N/A: not applicable.

^d^For text number count, Bonferroni post hoc analysis showed the following: human versus ChatGPT: *P*>.99; 95% CI –25.89 to –25.29; human versus Bard: *P*<.001; 95% CI 67.41-120.49; and ChatGPT versus Bard: *P*<.001; 95% CI 67.46-120.54.

### AI-Generated Abstract Formats and Content

With respect to abstracts that met the structured format requirements of the journals, 56.7% (34/60) of ChatGPT’s abstracts complied with the journal guidelines, but only 11.1% (6/54) of Bard’s abstracts matched the journal’s requirements because Bard only produced abstracts in the form of “Background, Methods, Results, Conclusions,” regardless of the journal specified in the prompt. For word count, the proportion deemed acceptable by the journals’ guidelines was significantly higher among Bard-generated abstracts (49/54, 90.7%) than among the ChatGPT group (30/60, 50%; *P*<.001). For consistency of conclusions, no statistically significant differences were seen between the ChatGPT and Bard groups (*P*=.85). For cohort sample size, Pearson correlation analysis revealed strong and significant correlations between the human and ChatGPT groups (*r*=0.955; *P*<.001) and between the human and Bard groups (*r*=0.953; *P*<.001; Table 2).

**Table 2:** Assessment of the reproducibility of artificial intelligence–generated abstracts.

Variables	ChatGPT^a^ (n=60)	Bard^b^ (n=54)	*P* value
Structured abstract format of compliance with the journal’s guideline (matched:unmatched)	34:26	6:48	<.001
The abstract word counts acceptable for journal’s guideline (acceptable:unacceptable)	30:30	49:5	<.001
Consistency of conclusions (consistent:inconsistent)	39:21	36:18	.85

^a^Correlation of the cohort’s sample size with human-written abstracts was analyzed by the Pearson correlation analysis: *r*=0.955; *P*<.001.

^b^Correlation of the cohort’s sample size with human-written abstracts was analyzed by the Pearson correlation analysis: *r*=0.953; *P*<.001.

### Plagiarism in AI-Generated Abstracts

A total of 106 of the 114 (84.2%) AI-generated abstracts met the criteria for plagiarism, with 94.4% (51/54) of the Bard-generated abstracts and 75% (45/60) of the ChatGPT abstracts meeting the threshold with statistical significance (*P*<.001; Table 3). Only 3 abstracts generated by Bard were not considered examples of plagiarism.

**Table 3:** Assessment of plagiarism in artificial intelligence–generated abstracts.

Variables	ChatGPT (n=60)	Bard (n=54)	*P* value
Plagiarism (+, similarity index≥15%)	45	51	<.001
Plagiarism (–, similarity index<15%)	15	3	<.001

### Evaluation of Abstracts by AI Detection Programs

**Figure 2:**
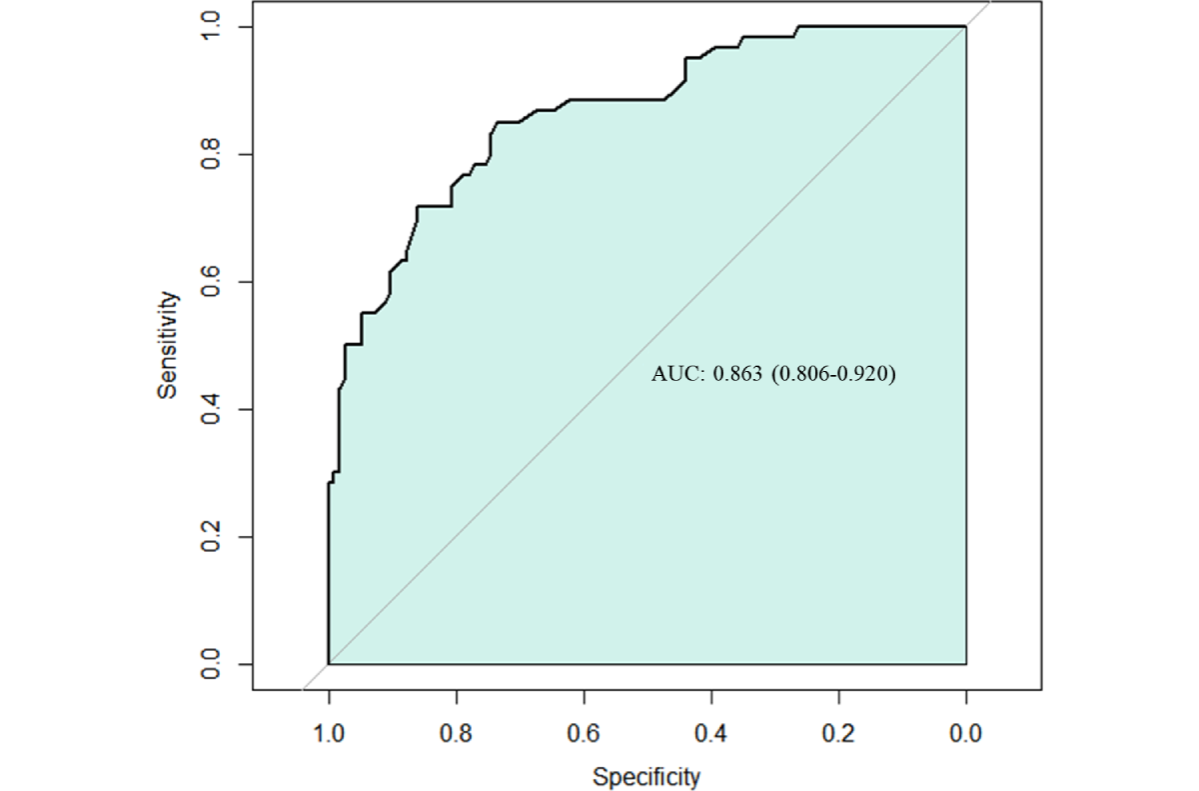
Assessment of artificial intelligence program detection. Receiver operative characteristics analysis showed an area under the curve of 0.863 (*P*<.001; 95% CI 0.806-0.920) and a cut-off value of 52.5%, with 73.7% sensitivity and 85% specificity. AUC: area under the curve.

ZeroGPT incorrectly identified 13 of the 60 (21.7%) human-authored abstracts as AI-generated. It successfully detected 74.6% (85/114) of the AI-generated abstracts, but 36.7% (22/60) of the ChatGPT abstracts were not detected. There was a statistically significant difference between the 3 groups (*P*<.001; Table 4). For ROC curve analysis of human-written and AI-generated abstracts, the AUC was 0.863 (*P*<.001; 95% CI 0.806-0.920), indicating robust models, and the cut-off value was 52.5% for the AI detection rate in the ZeroGPT, with 73.7% and 85% sensitivity and specificity, respectively (Figure 2). From the results of sentences presented by ZeroGPT, the AI programs successfully detected AI-generated abstracts with 74.6% and 78.3% sensitivity and specificity, respectively (Table 5).

**Table 4:** Assessment of AI^a^ program detection for human-written and AI-generated abstracts.

Variables	Human (n=60)	ChatGPT (n=60)	Bard (n=54)	*P* value
AI detection (+, detection rate≥50%)	13	38	47	<.001
AI detection (–, detection rate<50%)	47	22	7	<.001

^a^AI: artificial intelligence.

**Table 5:** AI^a^ program detection of abstracts using ZeroGPT in this study^b^.

Variables		Abstracts			Predictive value
		AI program^c^	Human^d^		
**Detection from ZeroGPT**					
	AI program	85	13	PPV^e^=86.7%	
	Human	29	47	NPV^f^=61.8%	

^a^AI: artificial intelligence.

^b^Sensitivity+specificity=154.9% (≥150%).

^c^Sensitivity=74.6%.

^d^Specificity=78.3%.

^e^PPV: positive predictive value.

^f^NPV: negative predictive value.

### Evaluation of the Abstracts by Human Reviewers

Using 8 human reviewers who had specialized in the spine field with more than 10 years of experience and the role of journal reviewer, the mean detection rate by human reviewers was 53.8% (SD 11.2%). Among the actual human-written abstracts, 48.8% (positive predictive values) were recognized as human-written, and of the AI-generated abstracts, 55.9% were recognized as AI-generated. Detection by human reviewers achieved a sensitivity of 56.3% and a specificity of 48.4% (Table 6).

**Table 6:** Blinded human reviewers’ detection of abstracts in this study^a^.

Variables		Abstracts			Predictive value
		Human^b^	AI^c^ program^d^		
**Assessment on human reviewers**					
	Human	63	66	PPV^e^=48.8%	
	AI program	49	62	NPV^f^=55.9%	

^a^The mean detection rate (%) for human-written abstracts=53.8 (SD 10.5), which were reviewed by 8 reviewers. Sensitivity+specificity=104.7% (<150%).

^b^Sensitivity=56.3%.

^c^AI: artificial intelligence.

^d^Specificity=48.4%.

^e^PPV: positive predictive value.

^f^NPV: negative predictive value.

## Discussion

### Principal Findings and Comparison With the Literature

Our findings demonstrated that both ChatGPT and Bard are capable of generating scientific abstracts from titles. Moreover, it is also noteworthy that the ability of LLM to produce abstracts has advanced to a level where it is challenging for humans to differentiate between AI-generated abstracts and human-written abstracts. The merits of writing a scientific paper using ChatGPT or Bard include the creation of a brief summary of complex research, rapid generation of suitable paragraphs, and visualization of important results in just a few seconds. Various trials of ChatGPT have been conducted, involving the writing of scientific papers, taking the United States Medical Licensing Examination, and expressing critical thinking [7,11]. However, these attempts have been met with concern by many experts, and the challenges posed by LLM-based AI have become the subject of social and ethical debates beyond the fields of medicine and science [2-4,12].

Controversies persist over whether AI-generated content itself has scientific value within ethical boundaries [5,9,12]. Debates weighing the advantages and limitations of AI language applications can be found in a variety of editorial forums [4,8-10]. However, to the best of our knowledge, few, if any, studies of the impact of ChatGPT on scientific writing have been conducted. Given the recent release date of Bard, no studies regarding Bard-generated scientific papers or comparing its abilities to those of ChatGPT of different LLMs are available. Gao et al [5] compared ChatGPT-generated abstracts with human-written scientific abstracts from 5 reputable journals, with an impact factor of over 87 (well quoted). However, little is known about the reproducibility of AI-generated abstracts in fields with a low citation index due to specialization, such as spinal surgery and neurosurgery [13-15].

We assessed the reproducibility of ChatGPT- and Bard-generated abstracts by comparing them with 60 human-written abstracts dealing with spine surgery. We also compared the differences between ChatGPT- and Bard-generated abstracts. Using iThenticate, ChatGPT- and Bard-generated abstracts had a mean similarity index greater than 15%, and a mean AI-detection rate greater than 60% reported by ZeroGPT indicates the current limitations of writing abstracts using only an AI program. The AI detection program did not identify all the AI-generated abstracts, achieving a sensitivity and specificity of 74.6% and 78.3%, respectively. However, these results indicate that AI programs have potential diagnostic value (sensitivity+specificity≥150%) to distinguish between human and AI authors. Contrary to the results achieved by the AI-detection program, humans who specialize in spine surgery were unable to distinguish between human-written and AI-generated abstracts (sensitivity+specificity<150%).

Our study revealed differences in the abstracts produced by ChatGPT and Bard, which use different LMMs [5,10,16]. ChatGPT is superior in creating structured abstracts that conform to journal guidelines but inferior in generating acceptable word counts. The contents, including consistency of conclusions and cohort sample sizes, of the ChatGPT-generated and Bard-generated abstracts did not differ significantly. One notable point in this study is related to 20% of hallucinations, which are AI’s own power and plausible ability to tell a lie. Our data showed 33.6% of hallucinations in the consistency of conclusions in spine abstracts [2,9]. It seems to have come out with a higher hallucination rate because the contents of the spine are a specialized area. This indicates that humans cannot easily distinguish between AI outputs, but it is clear that the current LMMs face limitations in writing spine abstracts in terms of the completeness of the format.

ChatGPT generated all requested abstracts in response to our prompts, but Bard failed to do so in 6 cases. The titles of 2 of the 6 papers were related to machine learning, and the other 4 involved low-prevalence diseases such as spinal tumors, a newly suggested scoring system, and surgical treatment. Our data suggest that Bard is less capable of generating scientific abstracts compared with ChatGPT in response to prompts referring to rare, poorly known, or new data. Nevertheless, given Bard’s characteristics, the program is likely to be used to provide scientific abstracts from unlimited sources connected to Google, and a reassessment is essential [16-18].

ChatGPT is superior with respect to meeting format requirements compared with Bard. The LMM of ChatGPT is based on a data-feed system with an information cutoff of September 2021. As a pretrained system, ChatGPT was able to generate abstracts that complied with journal guidelines. The trend in word count was larger in ChatGPT than in Bard. Language model for dialogue applications, the language model on which Bard is based, provides real-time information from Google, making it possible to provide an accurate summary of up-to-date paper contents. Bard-generated abstracts were more concise, with relatively low word counts compared with ChatGPT-generated abstracts. This result indicates that Bard has significant advantages for summarizing content concisely. However, in these LMMs, it is important to recognize the primary difference between the 2 AI programs: ChatGPT provides data-based, biased output, while Bard provides web-based biased output, and this can be reflected in the generated abstracts. Although Bard generated the web-based output from Google, some studies suggest Bard’s current limitations for accessing real-time data from Google [19,20]. For this controversial issue, our study also presented ChatGPT as superior in the formation of structured abstracts in compliance with the journal’s guidelines, with no differences in the consistency of conclusions as beneficial works for access to search engines. Bard is currently an experimental version of LLMs that has the potential to develop further in the future.

The sources of information were real-time internet data from Google in Bard and pretrained data up to 2021 in ChatGPT [5]. Despite these differences, no statistically significant differences in the consistency of conclusions between ChatGPT and Bard were evident. Furthermore, both AI programs suggested cohort sample sizes that were similar to those of human-written abstracts, based on data analyzed with Pearson correlations. This suggests that cohort size was an important factor in making the AI-generated abstracts indistinguishable from human reviewers. Importantly, the version of ChatGPT in this study was GPT-3.5, but newly the launched version (GPT-4) was considered more reliable, creative, and able to handle much more nuanced instructions [21]. Our further analysis demonstrated that GPT-4 showed better improvements in compliance with word count (from 30/60, 50% to 49/60, 81.7%) and consistency of conclusions (from 39/60, 65% to 42/60, 70%) than GPT-3.5. Thus, the capability for generating medical abstracts in GPT-4 may be superior to GPT-3.5, but it should be demonstrated through new studies in the future.

Our data on plagiarism using iThenticate reflected the distinctive characteristics of the 2 AI programs. The output from ChatGPT was regenerated in context using data published through 2021. Bard generates output from real-time information from the internet, producing a similarity index and plagiarism scores that were significantly higher than those of ChatGPT. AI-detection software determined that 84.2% (96/114) of the AI-generated abstracts included plagiarism.

ZeroGPT’s performance was relatively strong as measured by a ROC analysis (AUC=0.863; 95% CI 0.806-0.920). However, ZeroGPT had a limited detection value (73.7% sensitivity and 85% specificity in the ROC model) with respect to determining whether a spine section abstract was written by a human or AI. Similar results were produced for the expression of sentences (74.6% of sensitivity and 78.3% of specificity in sentence presentation). ZeroGPT also concluded (with sentences such as “Your text is AI/GPT Generated”) that 21.7% (13/60) of human-written abstracts were AI-generated abstracts, indicating a limit to practical applications. Therefore, in the spine field, human-written abstracts need to be verified through other methods to distinguish them from AI-generated text.

Contrary to the AI-detection program, our findings from blinded human reviewers’ detection of abstracts provided valuable insights into the writing of scientific abstracts. Because spine specialists were not able to effectively distinguish between human- and AI-generated abstracts, the use of AI programs may pose ethical challenges. Therefore, improvement of AI programs and AI detectors is needed to avoid ethical problems.

Májovský et al [22] described that LLMs can generate highly convincing fraudulent papers that mimic genuine scientific papers, from word usage to sentence structure. Our comprehensive analysis further confirms that LLMs’ ability to generate abstracts has evolved to a level that even experts find difficult to distinguish from authentic work. This implies that using LLMs carries the potential risk of producing completely fake papers. Therefore, authors of scientific papers should carefully weigh the risks and benefits of using LLMs. Moreover, within the scientific community, there is growing pressure to overhaul the peer review and publishing processes [23]. Researchers, including Májovský et al [24], who study the ability of AI to create scientific papers, have suggested several strategies to reduce the risk of fraudulent papers: the provision of source data sets publicly, a meticulous review process, strict ethical regulations at the level of publishers and academic institutions, and penalties for researchers who commit ethical misconduct. Given our findings on the capabilities of LLMs, the need to establish ethical standards will become increasingly essential, both for researchers (such as the notation of references about the use of ChatGPT) and the scientific community (such as the meticulous peer review process).

### Limitations

This study has several limitations. We evaluated a relatively small sample size and employed a few human reviewers. However, we collected as many abstracts as possible from representative journals and involved reviewers with vast experience in the relevant field. For the evaluation of human reviewers’ assessments, human reviewers might have been familiar with real abstracts, and this could have biased the results as a potential limitation. In addition, recent advances in the LLMs on which both ChatGPT and Bard are based were not incorporated into this study. Although those 2 models are currently in the development phase, our study provided important conclusions, including the reproducibility of scientific abstracts in the field of spinal surgery and the differences between ChatGPT and Bard. Our study was unable to capture the sensitive characteristics of the 2 representative AI models for the prompts. Because ChatGPT and Bard offer different outputs in response to slight changes in prompts, future evaluation of these findings is necessary. Last, the broad-scope prompt may affect the failure of the generation of 6 Bard-generated abstracts, despite the significant correlation between human- and AI-generated abstracts. Furthermore, the LLMs for generating abstracts are used after analyzing the main results of the studies. In other words, the main use of LLMs is to create abstracts based on the finished initial version of the manuscript. Instead of the title-based prompt or replacing the URLs with guideline instructions, alternative prompts may yield better results. Considering the importance of the concrete prompt before using LLMs, it should be addressed regarding the proper prompt for generating scientific abstracts in the future.

### Conclusions

Both ChatGPT and Bard can be used to help write scientific abstracts, but most AI-generated abstracts are currently considered unethical products based on high plagiarism and AI-detection rates. In terms of meeting journal formatting requirements, ChatGPT-generated abstracts appear to be superior to their Bard-generated counterparts. Because human reviewers are often unable to distinguish human writing from AI products, the use of AI programs to write abstracts within ethical boundaries requires careful consideration and should be evaluated through various approaches.

## Appendix

Multimedia Appendix 1 The outputs for generating abstracts based on prompt “Please write a scientific abstract for the article [title] in the style of [journal] at [link]” and human-written abstracts.

## Abbreviations

AI: artificial intelligence
AUC: area under the curve
LLM: large language model
ROC: receiver operative characteristics

## Data Availability

All data generated or analyzed during this study are included in this published paper (and in Multimedia Appendix 1).
