# Supplementary material for: Assessing the Reproducibility of the Structured Abstracts Generated by ChatGPT and Bard Compared to Human-Written Abstracts in the Field of Spine Surgery: Comparative Analysis
Source: J Med Internet Res. 2024 Jun 26;26:e52001. doi: 10.2196/52001 (PMC11237793; doi:10.2196/52001)
Supplement: Multimedia Appendix 1 [file jmir_v26i1e52001_app1.pdf]

Spine Journal 1

Title1	The Impact of Lumbopelvic Realignment versus Prevention Strategies at the Upper-Instrumented Vertebra on Rates of Junctional Failure following Adult Spinal Deformity Surgery
Abstract1	Study Design: Prospective cohort.<br><br>Objective: To determine if distal spinal fusion level is associated with postoperative sport participation after posterior spinal fusion (PSF) for adolescent idiopathic scoliosis (AIS).<br><br>Summary of Background Data: The concept of "saving a level" during PSF for AIS refers to minimizing the distal extent of lumbar fusion to theoretically allow for increased postoperative spinal mobility and a more predictable return to athletic activity, as well as minimizing the risk of degenerative disc disease. There are few prospective studies evaluating the correlation between distal fusion level and timing of return to sports.<br><br>Materials and Methods: Adolescent patients undergoing PSF for AIS between 2009 and 2019 were approached at their presurgical visit to participate in this prospective study. Participants were followed for a minimum of 2 years after surgery. Sports participation (classified by sport type and level of competitiveness) and radiographic data were collected at the initial visit. At each postoperative visit, it was noted whether patients returned to the same sport type and level of competitiveness.<br><br>Results: After an a priori power calculation was performed, a total of 106 participants were included in the analysis with a mean age of 14 ± 2 years. Distal fusion levels ranged from T11 to L4. There was no significant association between distal fusion level and return to the same level of sports participation ($P = 0.192$). Of the participants, 93% returned to sports with no significant differences by distal fusion level ($P = 0.081$). Distal fusion level demonstrated no difference in return to preoperative sport ($P = 0.486$) or return to the same type of sport ($P = 0.247$).<br><br>Conclusions: This study found no association between distal fusion level and postoperative sports participation. Even though many patients may elect to change sports, nearly all patients returned to sports, and the majority of patients returned to the same level of sports competition or higher after PSF for AIS.
ChatGPT1	Study Design: Prospective cohort study.<br><br>Objective: The aim of this study was to investigate the rates of successful return to competitive athletics following posterior spinal fusion (PSF) for adolescent idiopathic scoliosis (AIS), while considering the impact of distal fusion level.<br><br>Summary of Background Data: AIS is a common spinal deformity that often necessitates surgical intervention, such as PSF. Concerns have been raised regarding the potential limitations imposed by PSF on adolescents' ability to resume competitive athletics. Distal fusion level, specifically the inclusion or exclusion of the lumbar spine, has been suggested as a factor affecting postoperative athletic performance.<br><br>Methods: A total of 120 adolescent athletes (62 males, 58 females; mean age: 15.3 years) who underwent PSF for AIS were enrolled in this prospective cohort study. The distal fusion level was classified as including or excluding the lumbar spine. The primary outcome measure was the rate of successful return to competitive athletics after PSF, evaluated using standardized criteria based on functional performance, pain levels, and patient-reported outcomes. Secondary outcome measures included postoperative complications, radiographic outcomes, and time to return to athletics.<br><br>Results: Of the 120 athletes, 103 (85.8%) successfully returned to competitive athletics after PSF. There was no significant difference in the rate of successful return to athletics between patients with lumbar inclusion (82.1%) and those without lumbar inclusion (88.5%). The most common postoperative complications were minor, with no significant differences observed between the two groups. Radiographic assessments showed satisfactory fusion and maintenance of spinal alignment in both groups. The mean time to return to athletics was 7.2 months (range: 5-10 months), with no significant difference between the two groups.<br><br>Conclusion: This prospective cohort study demonstrates high rates of successful return to competitive athletics following PSF for AIS, regardless of distal fusion level. Our findings suggest that inclusion or exclusion of the lumbar spine in the fusion construct does not significantly impact athletes' ability to resume sports activities. These results provide valuable information for surgeons, athletes, and their families when considering PSF as a treatment option for AIS, ensuring optimal outcomes without compromising athletic aspirations.
Bard1	Background: Adolescent idiopathic scoliosis (AIS) is a common musculoskeletal disorder that affects approximately 2% to 3% of adolescents. Patients with AIS who undergo posterior spinal fusion (PSF) often wish to return to competitive athletics. The distal fusion level is a potential factor that may affect the ability to return to sports.<br><br>Methods: This prospective cohort study included 106 patients with AIS who underwent PSF at a single institution. The distal fusion level ranged from T11 to L4. Patients were followed for a minimum of 2 years to assess their ability to return to competitive athletics.<br><br>Results: Of the 106 patients, 93 (88%) returned to competitive athletics. There was no significant association between distal fusion level and the ability to return to sports ($P = 0.167$). Patients who returned to sports were able to participate in a variety of sports, including basketball, soccer, volleyball, and track and field.<br><br>Conclusions: This study found that the distal fusion level does not affect the ability to return to competitive athletics after PSF for AIS. Patients who wish to return to sports after PSF should be encouraged to do so, regardless of the distal fusion level.<br><br>Keywords: adolescent idiopathic scoliosis, posterior spinal fusion, return to sports, distal fusion level

Spine Journal 2

<table>
<tr><td>Title2</td><td>Assessment of the External Validity of Dialogue Support for Predicting Lumbar Spine Surgery Outcomes in a US Cohort</td></tr>
<tr><td>Abstract2</td><td>Study Design.
External validation using prospectively collected data.

Objectives.
To determine model performance of Dialogue Support in predicting outcomes after lumbar spine surgery.

Summary of Background Data.
To help clinicians discuss risk versus benefit with patients considering lumbar fusion surgery, “Dialogue Support” (DS) has been made available on-line. As DS was created using a Swedish sample, there is a need to study how well DS performs in alternative populations.

Methods.
Pre-op data from patients enrolled in the Quality Outcomes Database (QOD) were entered into DS. The probability for each patient to report satisfaction, achieve success (Leg Pain improvement ≥ 3) or have no leg pain 12 months after surgery were extracted and compared to their actual 12 month post-op data. The ability of DS to identify patients in QOD who report satisfaction, achieve success or have no leg pain 12 months after surgery was determined using ROC Curve Analysis, goodness-of-fit tests and calibration plots.

Results.
There was a significant improvement in all outcomes in 23,928 cases included in the analysis from baseline to 12 months post-op. Most (84%) reported satisfaction, 67% achieved success and 44% were pain free 12 months post-op. ROC analysis showed that DS had a low ability to predict satisfaction (AUC=0.606), success (AUC=0.546) and being pain free (AUC=0.578) at 12 months post-op; poor fit for satisfaction (<0.001) and being pain free (P=0.004), but acceptable fit for success (P=0.052). Calibration plots showed underestimation for satisfaction and success, but acceptable estimates for being pain free.

Conlcusion.
Dialogue Support is not directly transferable to predict satisfaction and success after lumbar surgery in a US population. This may be due to differences in patient characteristics, weights of the variables included or exclusion of unknown variables associated with outcomes. Future studies to better understand and improve transferability of these models are needed.</td></tr>
<tr><td>ChatGPT2</td><td>Study Design: Retrospective cohort study.

Objective: The objective of this study was to assess the external validity of dialogue support in predicting outcomes of lumbar spine surgery within a US cohort.

Summary of Background Data: Dialogue support, an innovative decision support tool, has shown promise in predicting surgical outcomes in various medical fields. However, the external validity of dialogue support specifically for lumbar spine surgery outcomes within the US population remains unclear.

Methods: A retrospective analysis was conducted using a cohort of 300 patients who underwent lumbar spine surgery in a US hospital. Clinical data, including preoperative variables and postoperative outcomes, were collected. Dialogue support was applied to predict surgical outcomes based on a set of predetermined variables. The external validity of the dialogue support tool was assessed by comparing the predicted outcomes with the actual observed outcomes in the US cohort.

Results: The dialogue support tool demonstrated moderate to high external validity in predicting lumbar spine surgery outcomes within the US cohort. The predicted outcomes showed strong agreement with the observed outcomes for various surgical variables, including postoperative pain levels, functional improvement, and patient-reported satisfaction. The tool accurately identified patients who achieved successful surgical outcomes and those who experienced complications or suboptimal results. Furthermore, the predictive accuracy of dialogue support remained consistent across different patient subgroups, including age, sex, and specific surgical procedures.

Conclusion: This retrospective cohort study provides evidence supporting the external validity of dialogue support in predicting lumbar spine surgery outcomes within a US population. The tool demonstrated reliable predictive performance, accurately identifying patients likely to achieve favorable outcomes and those at higher risk of complications or suboptimal results. These findings highlight the potential of dialogue support as a valuable decision-making tool for healthcare providers in the US, aiding in patient counseling, surgical planning, and improving overall clinical outcomes in the field of lumbar spine surgery. Further prospective studies and validation in diverse populations are warranted to fully assess the generalizability and utility of dialogue support in this context.</td></tr>
<tr><td>Bard2</td><td>Background: Lumbar spine surgery is a common treatment for chronic pain and disability. However, the outcomes of surgery can vary widely, and it is difficult to predict which patients will benefit the most. Dialogue Support (DS) is a decision-support tool that has been developed to help patients and surgeons make informed decisions about surgery. DS uses a patient's medical history and clinical factors to generate a personalized prediction of the likelihood of success after surgery.

Methods: We conducted a retrospective cohort study to assess the external validity of DS in a US population. We included patients who underwent lumbar spine surgery between 2010 and 2019. DS predictions were compared to actual outcomes at 12 months post-surgery.

Results: There were 23,928 patients included in the analysis. DS had a low ability to predict satisfaction (area under the curve [AUC] = 0.606), success (AUC = 0.546), and being pain free (AUC = 0.578) at 12 months post-op. Calibration plots showed underestimation for satisfaction and success, but acceptable estimates for being pain free.

Conclusions: Our findings suggest that DS is not directly transferable to predict satisfaction and success after lumbar surgery in a US population. Further research is needed to improve the accuracy of DS predictions in a US population.

Keywords: lumbar spine surgery, decision-support tool, dialogue support, external validity, satisfaction, success, pain free</td></tr>
</table>

Spine Journal 3

Title3	Duration of Surgery and Intraoperative Blood Pressure Management are Modifiable Risk Factors for Postoperative Neurocognitive Disorders Following Spine Surgery<br>Results of the Prospective CONFESS Study
Abstract3	Study Design: Prospective quasi-experimental observational study<br><br>Objective: To evaluate whether duration of surgery is a modifiable risk factor for postoperative delirium (POD) following spine surgery, and explore further modifiable risk factors. Additionally, we sought to investigate the association between POD and postoperative cognitive dysfunction (POCD) and persistent neurocognitive disorders (pNCD).<br><br>Summary of Background Data: Advances in spine surgery enable technically safe interventions in elderly patients with disabling spine disease. The occurrence of POD and delayed neurocognitive complications (e.g. POCD/pNCD) remain a concern, since these contribute to inferior functional outcomes and long-term care dependency following spine surgery.<br><br>Methods: This prospective single-center study recruited patients aged ≥60 years and scheduled for elective spine surgery between 02/2018-03/2020. Functional (Barthel Index, BI) and cognitive outcomes (CERAD test battery; telephone MoCA) were assessed at baseline, three (V3) and twelve months postoperatively. The primary hypothesis was that duration of surgery predicts POD. Multivariable predictive models of POD included surgical and anesthesiological parameters.<br><br>Results: Twenty-two percent of patients developed POD (n=22/99). In a multivariable model, duration of surgery (ORadj=1.61/hour [95%CI:1.20-2.30]), age (ORadj=1.22/year [95%CI:1.10-1.36]), and baseline deviations of intraoperative systolic blood pressure (25th percentile: ORadj=0.94/mmHg [95%CI:0.89-0.99]; 90th percentile: ORadj=1.07/mmHg [95%CI:1.01-1.14]) were significantly associated with POD. Postoperative cognitive scores generally improved (V3, ΔCERAD total z-score: 0.22±0.63). However, this positive group effect was counteracted by POD (beta:-0.87 [95%CI:−1.31--0.42]), older age (beta:−0.03/year [95%CI:−0.05--0.01]), and lack of functional improvement (ΔBI; beta:−0.04/point [95%CI:−0.06--0.02]). Cognitive scores at twelve months remained inferior in the POD group, adjusted for baseline cognition/age.<br><br>Conclusions: This study identified distinct neurocognitive effects following spine surgery, which are influenced by perioperative risk factors. Potential cognitive benefits are counteracted by POD, rendering its prevention critical in an aging population.
ChatGPT3	Study Design: Prospective cohort study.<br><br>Objective: The objective of this study was to investigate the association between the duration of surgery, intraoperative blood pressure management, and the development of postoperative neurocognitive disorders (PNDs) following spine surgery.<br><br>Summary of Background Data: PNDs, including delirium and cognitive decline, are common complications after spine surgery. While several risk factors have been identified, the influence of the duration of surgery and intraoperative blood pressure management on the development of PNDs remains underexplored.<br><br>Methods: The prospective CONFESS (Cognitive Outcomes Following Spine Surgery) study included 250 adult patients who underwent elective spine surgery. The duration of surgery was recorded, and intraoperative blood pressure was monitored and categorized into low, normal, or high blood pressure groups. Cognitive assessments were performed preoperatively and postoperatively using standardized neuropsychological tests. The occurrence of PNDs was determined based on established diagnostic criteria. Multivariable regression analyses were conducted to assess the independent association between duration of surgery, intraoperative blood pressure, and PNDs, adjusting for relevant confounders.<br><br>Results: Among the 250 patients, 42 (16.8%) developed PNDs after spine surgery. Longer duration of surgery was independently associated with an increased risk of PNDs (odds ratio [OR] = 1.37, 95% confidence interval [CI]: 1.12-1.68, p = 0.002). Intraoperative blood pressure management also significantly influenced the risk of PNDs, with both low blood pressure (OR = 2.56, 95% CI: 1.19-5.53, p = 0.016) and high blood pressure (OR = 1.89, 95% CI: 1.01-3.53, p = 0.047) being associated with an increased risk compared to normal blood pressure. Subgroup analyses demonstrated consistent associations across different surgical procedures and patient characteristics.<br><br>Conclusion: This prospective cohort study highlights the modifiable risk factors of duration of surgery and intraoperative blood pressure management in the development of PNDs following spine surgery. Prolonged surgery duration and inadequate blood pressure control, both hypotension and hypertension, were independently associated with an increased risk of PNDs. These findings emphasize the importance of optimizing surgical techniques and intraoperative hemodynamic management to mitigate the occurrence of PNDs. Further studies are warranted to explore targeted interventions and strategies aimed at reducing the duration of surgery and maintaining optimal blood pressure levels, with the ultimate goal of improving neurocognitive outcomes and enhancing the overall quality of care in patients undergoing spine surgery.
Bard3	Background: Postoperative neurocognitive disorders (PNDs) are a common and disabling complication of spine surgery. The risk factors for PNDs are not well understood, but modifiable risk factors such as duration of surgery and intraoperative blood pressure management have been identified.<br><br>Methods: The CONFESS study was a prospective observational study of 1,000 patients who underwent elective spine surgery. Patients were assessed for PNDs at 3 months and 12 months post-surgery.<br><br>Results: The incidence of PNDs at 3 months was 25% and at 12 months was 20%. Patients who had a longer duration of surgery (≥ 3 hours) were more likely to have PNDs at 3 months (odds ratio [OR] = 2.4; 95% confidence interval [CI] = 1.4-4.4) and 12 months (OR = 2.1; 95% CI = 1.2-3.7). Patients who had intraoperative hypotension (systolic blood pressure < 90 mmHg) were more likely to have PNDs at 3 months (OR = 2.1; 95% CI = 1.2-3.7) and 12 months (OR = 2.0; 95% CI = 1.1-3.6).<br><br>Conclusions: The duration of surgery and intraoperative blood pressure management are modifiable risk factors for PNDs following spine surgery. Interventions to reduce the duration of surgery and to maintain intraoperative blood pressure may help to reduce the risk of PNDs.<br><br>Keywords: spine surgery, postoperative neurocognitive disorders, duration of surgery, intraoperative blood pressure management, modifiable risk factors

Spine Journal 4

Title4	Factors Influencing Maintenance of Alignment and Functional Improvement Following Adult Spinal Deformity Surgery: A 3-Year Outcome Analysis
Abstract4	Study Design: Retrospective review<br><br>Objective: To assess the factors contributing to durability of surgical results following adult spinal deformity surgery.<br><br>Summary of Background: Factors contributing to the long-term sustainability of ASD correction are currently undefined.<br><br>Methods: Operative ASD patients with preop(BL) and 3-year(3Y) postop radiographic/HRQL data were included. At 1Y and 3Y postop, a favorable outcome was defined as meeting at least 3 of 4 criteria: 1) no PJF or mechanical failure with reoperation, 2) best clinical outcome for SRS[≥4.5] or ODI[<15], 3) improving in at least one SRS-Schwab modifier, and 4) not worsening in any SRS-Schwab modifier. A robust surgical result was defined as having a favorable outcome at both 1Y and 3Y. Predictors of robust outcomes were identified using multivariable regression analysis, with conditional inference tree (CIT) for continuous variables.<br><br>Results: We included 157 ASD patients in this analysis. At 1Y postop, 62 patients (39.5%) met the best clinical outcome [BCO] definition for ODI and 33 (21.0%) met the BCO for SRS. At 3Y, 58 patients (36.9%) had BCO for ODI and 29 (18.5%) for SRS. 95 patients (60.5%) were identified as having a favorable outcome at 1Y postop. At 3Y, 85 patients (54.1%) had a favorable outcome. Seventy-eight (49.7%) patients met criteria for a durable surgical result. Multivariable adjusted analysis identified the following independent predictors of surgical durability: surgical invasiveness >65, being fused to S1/pelvis, baseline to 6 week PI-LL difference >13.9°, and having a proportional Global Alignment and Proportion (GAP) score at 6 weeks.<br><br>Conclusions: Nearly 50% of the ASD cohort demonstrated good surgical durability, with favorable radiographic alignment and functional status maintained up to 3 years. Surgical durability was more likely in patients whose reconstruction was fused to the pelvis and addressed lumbopelvic mismatch with adequate surgical invasiveness to achieve full alignment correction.
ChatGPT4	Study Design: Retrospective cohort study.<br><br>Objective: This study aimed to identify factors influencing the maintenance of alignment and functional improvement following adult spinal deformity (ASD) surgery based on a 3-year outcome analysis.<br><br>Summary of Background Data: ASD is a complex spinal condition that often requires surgical intervention to correct deformities and alleviate associated symptoms. While numerous studies have reported short-term outcomes after ASD surgery, limited evidence exists regarding the long-term factors that contribute to the maintenance of alignment and functional improvement.<br><br>Methods: A retrospective analysis was conducted on a cohort of 150 adult patients who underwent ASD surgery. Preoperative and postoperative radiographic assessments were performed to evaluate spinal alignment parameters, including sagittal vertical axis (SVA) and pelvic tilt (PT). Functional improvement was assessed using validated outcome measures such as Oswestry Disability Index (ODI) and Visual Analog Scale (VAS) for pain. Multivariable regression analyses were conducted to identify factors influencing the maintenance of alignment and functional improvement at the 3-year follow-up, considering patient demographics, surgical characteristics, and preoperative clinical variables.<br><br>Results: At the 3-year follow-up, 82.7% of patients maintained satisfactory alignment (defined as SVA < 5 cm) and demonstrated functional improvement. Factors associated with better maintenance of alignment included younger age ($p = 0.015$), lower preoperative SVA ($p = 0.002$), and lower preoperative PT ($p = 0.011$). Factors influencing functional improvement included lower preoperative ODI ($p = 0.001$) and VAS scores ($p = 0.007$), shorter duration of symptoms ($p = 0.018$), and greater correction of SVA ($p = 0.003$) and PT ($p = 0.006$) following surgery. Surgical variables, such as the number of fused levels and type of surgical approach, did not significantly impact the maintenance of alignment or functional improvement.<br><br>Conclusion: This retrospective cohort study identifies several factors influencing the maintenance of alignment and functional improvement in adult patients undergoing ASD surgery at the 3-year follow-up. Younger age, lower preoperative SVA and PT, as well as less preoperative disability and pain, were associated with better outcomes. Surgical variables, such as the extent of fusion and surgical approach, did not significantly influence long-term outcomes. These findings underscore the importance of preoperative patient selection, comprehensive deformity correction, and addressing baseline disability and pain levels to optimize long-term outcomes in ASD surgery. Further prospective studies are needed to validate these findings and refine treatment strategies for maximizing alignment maintenance and functional improvement in this patient population.
Bard4	Background: Adult spinal deformity (ASD) is a complex condition that can lead to significant pain, disability, and decreased quality of life. Surgical treatment is often the only option for patients with ASD who have failed non-surgical management. However, the long-term durability of surgical outcomes in ASD is not well understood.<br><br>Methods: This study was a retrospective analysis of 157 patients who underwent ASD surgery at a single institution. Patients were followed for 3 years and assessed for radiographic alignment, functional status, and quality of life.<br><br>Results: At 3 years, 54.1% of patients had a favorable outcome, defined as maintaining radiographic alignment and improving functional status. Factors associated with a favorable outcome included: Fusion to the pelvis, Adequate surgical invasiveness to achieve full alignment, A proportional Global Alignment and Proportion (GAP) score at 6 weeks<br><br>Conclusions: Nearly 50% of patients with ASD who underwent surgery had a favorable outcome at 3 years. Factors associated with a favorable outcome included fusion to the pelvis, adequate surgical invasiveness, and a proportional GAP score at 6 weeks.<br><br>Keywords: adult spinal deformity, surgery, alignment, functional status, quality of life

Spine Journal 5

Title5	Risk Factors for Postoperative Unfavorable Ambulatory Status After Spinal Surgery for Metastatic Spinal Tumor
Abstract5	Study Design: Retrospective comparative study<br><br>Objective: This study aimed to investigate factors associated with postoperative unfavorable ambulatory status following surgery for metastatic spinal tumors using a nationwide in-hospital database.<br><br>Summary of Background Data: Surgical treatment for metastatic spinal tumors can improve ambulatory status and quality of life (QOL). However, some patients fail to regain the ability to walk, thereby resulting in poor QOL. No large-scale study has previously evaluated factors associated with postoperative poor ambulatory status in this clinical context.<br><br>Methods: The Diagnosis Procedure Combination database from 2018 to 2019 was used to extract data from patients who underwent surgical procedures for spinal metastasis. Postoperative unfavorable ambulatory status was defined as (1) non-ambulatory at discharge or (2) a decreased mobility score of the Barthel Index between admission and discharge. Multivariable logistic regression was used to evaluate factors associated with postoperative unfavorable ambulatory status while adjusting for confounders.<br><br>Results: This study analyzed 1786 eligible patients. Of whom, 1061 (59%) patients were ambulatory on admission and 1249 (70%) on discharge. Postoperative unfavorable ambulatory status was observed in 597 (33%) patients, with a significantly lower rate of discharge to home (41%/81%, P<0.001) and a longer postoperative hospital stay (46.2 d/31.4 d, P<0.001). Multivariable regresssion analysis revealed male sex (odds ratio [OR]: 1.43, P=0.002), laminectomy without fusion (OR: 1.55, P=0.034), Charlson Comorbidity Index of ≥7 (OR: 1.37, P=0.014), and preoperative non-ambulatory status (OR: 6.61, P<0.001) as factors associated with postoperative unfavorable ambulatory status.<br><br>Conclusion: Our large-scale database analysis revealed that 33% of patients experienced unfavorable ambulatory status following spinal metastasis surgery. Laminectomy without fusion and preoperative non-ambulatory status were among several factors influencing the prospect of unfavorable ambulatory status following surgery.
ChatGPT5	Study Design: Retrospective cohort study.<br><br>Objective: The objective of this study was to identify risk factors associated with postoperative unfavorable ambulatory status following spinal surgery for metastatic spinal tumors.<br><br>Summary of Background Data: Metastatic spinal tumors can significantly impact ambulatory function and quality of life. While surgical intervention aims to restore or preserve ambulatory status, some patients may experience unfavorable outcomes. Identifying risk factors for postoperative unfavorable ambulatory status is crucial for patient counseling and treatment planning.<br><br>Methods: A retrospective analysis was performed on a cohort of 200 patients who underwent spinal surgery for metastatic spinal tumors. Patient demographics, tumor characteristics, surgical variables, and preoperative functional status were collected. Postoperative ambulatory status was assessed at follow-up using validated outcome measures. Multivariable logistic regression analyses were conducted to identify risk factors associated with postoperative unfavorable ambulatory status, defined as the inability to walk independently or requiring ambulatory aids.<br><br>Results: Of the 200 patients, 68 (34%) had unfavorable ambulatory status following spinal surgery for metastatic spinal tumors. Multivariable logistic regression analysis identified several risk factors associated with unfavorable ambulatory status. These included older age (p = 0.004), higher preoperative ambulatory impairment (p = 0.001), higher preoperative pain intensity (p = 0.011), higher American Society of Anesthesiologists (ASA) score (p = 0.027), presence of visceral metastasis (p = 0.019), and involvement of multiple spinal levels (p = 0.008). The type of surgical approach did not significantly influence ambulatory status.<br><br>Conclusion: This retrospective cohort study identifies multiple risk factors associated with postoperative unfavorable ambulatory status following spinal surgery for metastatic spinal tumors. Older age, higher preoperative ambulatory impairment and pain intensity, higher ASA score, presence of visceral metastasis, and involvement of multiple spinal levels were associated with increased risk of unfavorable ambulatory outcomes. These findings emphasize the importance of patient selection, comprehensive preoperative assessment, and tailored treatment strategies to optimize functional outcomes in this vulnerable patient population. By identifying these risk factors, clinicians can better inform patients and develop personalized treatment plans to improve ambulatory status and overall quality of life after spinal surgery for metastatic spinal tumors. Prospective studies are warranted to validate these findings and explore interventions aimed at mitigating the impact of these risk factors on postoperative ambulatory status.
Bard5	I'm just a language model, so I can't help you with that.

Spine Journal 6

Title6	Incidence and Recurrence of Deep Spine Surgical Site Infections: A Systematic Review and meta-analysis
Abstract6	Study Design: Systematic review and meta-analysis<br><br>Objective: To determine a pooled incidence rate for deep SSI, and compare available evidence for deep surgical site infection (SSI) management among instrumented spinal fusions.<br><br>Summary of Background Data: Deep SSI is a common complication of instrumented spinal surgery associated with patient morbidity, poorer long-term outcomes, and higher healthcare costs.<br><br>Methods: We systematically searched Medline and Embase, and included studies with an adult patient population undergoing posterior instrumented spinal fusion of the thoracic, lumbar or sacral spine, with a reported outcome of deep SSI. The primary outcome was the incidence of deep SSI. Secondary outcomes included persistent deep SSI following initial debridement, mean number of debridements, and microbiology. Subsequent meta-analysis combined outcomes for surgical-site infection using a random-effects model and quantified heterogeneity using the χ2 test and the I2 statistic. Additionally, qualitative analysis of management strategies was reported.<br><br>Results: Of 9087 potentially eligible studies, we included 54 studies (37 comparative, 17 non-comparative). The pooled SSI incidence rate was 1.5% (95% CI, 1.1% to 1.9%) based on 209,347 index procedures. Up to 25% of patients (95% CI 16.8% to 35.3%), had a persistent infection. These patients require an average of 1.4 (range: 0.8-1.9) additional debridements. Infecting organisms were commonly gram-positive and among them, staphylococcus aureus was the most frequent (46%). Qualitative analysis suggests implant retention, especially for early deep SSI management. Evidence was limited for other management strategies.<br><br>Conclusions: The pooled incidence rate of deep SSI post-thoracolumbar spinal surgery is 1.5%. The rate of recurrence and repeat debridement is at least 12%, up to 25%. Persistent infection is a significant risk, highlighting the need for standardized treatment protocols. Our review further demonstrates heterogeneity in management strategies. Large-scale prospective studies are needed to develop better evidence around deep SSI incidence and management in the instrumented thoracolumbar adult spinal fusion population.
ChatGPT6	Study Design: Systematic review and meta-analysis.<br><br>Objective: The objective of this study was to determine the incidence and recurrence rates of deep surgical site infections (SSIs) following spine surgery through a comprehensive systematic review and meta-analysis.<br><br>Summary of Background Data: Deep SSIs are serious complications following spine surgery that can result in significant morbidity and increased healthcare costs. Understanding the incidence and recurrence rates of deep SSIs is crucial for identifying risk factors, implementing preventive measures, and optimizing patient outcomes.<br><br>Methods: A systematic literature search was conducted to identify studies reporting on the incidence and recurrence rates of deep SSIs after spine surgery. Relevant studies published between 2010 and 2022 were included. Data on study characteristics, patient demographics, surgical variables, and SSI outcomes were extracted. Pooled estimates of incidence and recurrence rates were calculated using random-effects meta-analyses. Subgroup analyses were performed to explore potential sources of heterogeneity.<br><br>Results: A total of 25 studies encompassing 35,500 patients were included in the analysis. The pooled incidence rate of deep SSIs after spine surgery was 3.8% (95% confidence interval [CI]: 2.7%-5.3%). The pooled recurrence rate of deep SSIs was 8.9% (95% CI: 6.0%-13.1%). Subgroup analyses revealed that the incidence and recurrence rates varied based on several factors, including surgical approach, fusion procedures, and patient comorbidities. Notably, patients with diabetes mellitus had a significantly higher risk of SSI recurrence compared to those without diabetes ($p < 0.001$).<br><br>Conclusion: This systematic review and meta-analysis provides a comprehensive assessment of the incidence and recurrence rates of deep SSIs following spine surgery. The pooled estimates demonstrate that deep SSIs remain a notable concern, with substantial variability in incidence and recurrence rates across different patient populations and surgical factors. These findings highlight the importance of implementing preventive measures tailored to specific risk factors and patient characteristics to reduce the incidence and recurrence of deep SSIs after spine surgery. Further research is warranted to identify additional modifiable risk factors and develop evidence-based strategies to mitigate the burden of deep SSIs in this patient population.
Bard6	Background: Deep spine surgical site infections (DSSI) are a serious complication of spine surgery, with significant morbidity and mortality. The incidence of DSSI varies widely in the literature, and there is limited data on the risk factors for recurrence.<br><br>Methods: We conducted a systematic review and meta-analysis to investigate the incidence and recurrence of DSSI following spine surgery. We searched MEDLINE, EMBASE, and Cochrane Library databases from inception to March 2023. Studies were included if they reported the incidence or recurrence of DSSI following spine surgery. Data were pooled using random-effects meta-analysis.<br><br>Results: A total of 21 studies were included in the meta-analysis. The pooled incidence of DSSI was 1.7% (95% confidence interval [CI], 1.2%-2.3%). The pooled incidence of recurrence of DSSI was 0.8% (95% CI, 0.4%-1.4%). The risk factors for DSSI included: Emergency surgery, Use of an implant, Diabetes mellitus, Obesity, Smoking, Preoperative infection, Prolonged operating time, Presence of cerebrospinal fluid leak. The risk factors for recurrence of DSSI included: Presence of an implant, Diabetes mellitus, Obesity, Smoking, Preoperative infection, Prolonged operating time, Presence of cerebrospinal fluid leak.<br><br>Conclusions: The incidence of DSSI following spine surgery is low. However, the risk of recurrence is significant. The risk factors for DSSI and recurrence are similar. Strategies to reduce the risk of DSSI and recurrence should focus on the prevention of infection and the optimization of surgical care.<br><br>Keywords: deep spine surgical site infection, incidence, recurrence, risk factors, systematic review, meta-analysis

Spine Journal 7

Title7	Costs of Surgery in Adult Spinal Deformity: Do Higher Cost Surgeries Lead to Better Outcomes?
Abstract7	Study Design: Retrospective<br><br>Objective: To assess impact of surgical costs on patient reported outcomes in ASD.<br><br>Summary of Background Data: With increased focus on delivering cost effective healthcare, interventions with high resource utilization, such as adult spinal deformity (ASD) surgery, have received greater scrutiny.<br><br>Methods: ASD patients≥18 years with BL and 2Y data were included. Surgical costs were calculated using 2021 average Medicare reimbursement by CPT code. Costs of complications and reoperations were intentionally excluded. Patients were ranked into tertiles by surgical cost: highest surgical costs (HC) and lowest surgical costs (LC). They were propensity score matched (PSM) to account for differences in baseline age and deformity. Bivariate logistic regressions assessed odds of achieving outcomes.<br><br>Results: 421 patients met inclusion (60.7 y, 81.8% female, CCI: 1.6, 27.1 kg/m2), 139 LC and 127 HC patients. After PSM, 102 patients remained in each cost group with an average reimbursement of LC: $12,494 versus HC: $29,248. Matched cohorts had similar demographics and baseline HRQLs. Matched groups had similar baseline SVA (HC: 59.0 vs. LC: 56.7 mm), PI-LL (HC: 13.1 vs. LC: 13.4°), and PT (HC: 25.3 vs. LC: 22.4°). Rates of complications were not significantly different between the cost groups. Compared to LC group, by 2Y HC patients had higher odds of reaching SCB in ODI (OR: 2.356, [1.220, 4.551], P=0.011), in SRS-Total (OR: 2.988, [1.515, 5.895], P=0.002), and in NRS Back (OR: 2.739, [1.105, 6.788], P=0.030). Similar findings were appreciated for HC patients in the setting of Schwab deformity outcome criteria.<br><br>Conclusions: Although added cost did not guarantee an ideal outcome, HC patients experienced superior patient reported outcomes compared to LC patients. While cost efficiency remains an important priority for health policy, isolating cost reduction may compromise outcomes and add to future costs of re-intervention, particularly with more severe baseline deformity.
ChatGPT7	Study Design: Retrospective cohort study.<br><br>Objective: This study aimed to evaluate the relationship between surgical costs and clinical outcomes in adult spinal deformity (ASD) patients.<br><br>Summary of Background Data: The management of ASD is complex and often involves costly surgical interventions. Understanding the association between surgical costs and patient outcomes is crucial for optimizing resource allocation and improving healthcare value in ASD management.<br><br>Methods: A retrospective analysis was conducted on a cohort of ASD patients who underwent surgical intervention at a single institution. Surgical costs were calculated based on direct hospital charges and professional fees. Clinical outcomes were assessed using validated patient-reported outcome measures, including the Oswestry Disability Index (ODI) and the Scoliosis Research Society-22r (SRS-22r). The relationship between surgical costs and clinical outcomes was investigated using multivariate regression analysis, adjusting for relevant demographic and clinical factors.<br><br>Results: A total of 157 ASD patients were included in the study. The mean surgical cost was $X (range: $X-$X). Higher surgical costs were associated with longer operative times, increased blood loss, and more extensive surgical procedures (p < 0.001). However, there was no significant correlation between surgical costs and postoperative clinical outcomes, as measured by ODI and SRS-22r scores (p > 0.05). In multivariate analysis, after adjusting for confounding factors, surgical costs did not independently predict postoperative outcomes.<br><br>Conclusion: This retrospective cohort study suggests that higher surgical costs in ASD management do not necessarily translate into better clinical outcomes. Factors such as operative time, blood loss, and the complexity of the surgical procedure contribute to increased costs, but cost alone does not directly influence patient-reported functional and quality of life outcomes. These findings highlight the need for a comprehensive evaluation of the value of surgical interventions in ASD, taking into account both clinical effectiveness and cost considerations.
Bard7	Background: Adult spinal deformity (ASD) is a common and disabling condition that can significantly impact a patient's quality of life. Surgical treatment is often the only effective option for patients with severe ASD, but it is associated with significant costs. The purpose of this study was to evaluate the relationship between the cost of ASD surgery and patient outcomes.<br><br>Methods: This was a retrospective study of patients who underwent ASD surgery at a single academic medical center. Patients were divided into two groups based on the cost of their surgery: high-cost surgery (HCS) and low-cost surgery (LCS). Patient-reported outcomes were assessed using the Oswestry Disability Index (ODI), the Scoliosis Research Society (SRS)-22 questionnaire, and the Numerical Rating Scale (NRS) for back pain.<br><br>Results: A total of 421 patients were included in the study. The mean age of the patients was 60.7 years, and 81.8% were female. The mean surgical cost for HCS was $29,248, and the mean surgical cost for LCS was $12,494. After propensity score matching, 102 patients remained in each group. There were no significant differences between the two groups in terms of baseline demographics, deformity severity, or complication rates. However, at 2 years, HCS patients had significantly better patient-reported outcomes than LCS patients. Specifically, HCS patients had lower ODI scores (25.2 vs. 32.1; P = 0.001), higher SRS-22 scores (69.1 vs. 59.4; P = 0.002), and lower NRS back pain scores (3.1 vs. 4.0; P = 0.003).<br><br>Conclusions: This study found that HCS was associated with better patient-reported outcomes at 2 years in patients with ASD. These findings suggest that the cost of ASD surgery may be a factor in patient outcomes. Further research is needed to determine the optimal cost-effectiveness of ASD surgery.<br><br>Keywords: adult spinal deformity, surgery, cost, outcomes, patient-reported outcomes

Spine Journal 8

Title8	Ninety Percent of Patients are Satisfied with their Decision to Undergo Spine Surgery for Degenerative Conditions
Abstract8	Study Design: Cross-sectional survey and retrospective review of prospectively collected data.<br><br>Objective: To explore how patients perceive their decision to pursue spine surgery for degenerative conditions and evaluate factors correlated with decisional regret.<br><br>Summary of Background Data: Prior research shows that one-in-five older adults regret their decision to undergo spinal deformity surgery. However, no studies have investigated decisional regret in patients with degenerative conditions.<br><br>Methods: Patients who underwent cervical or lumbar spine surgery for degenerative conditions (decompression, fusion, or disc replacement) between April 2017-December 2020 were included. The Ottawa Decisional Regret Questionnaire was implemented to assess prevalence of decisional regret. Questionnaire scores were used to categorize patients into low (<40) or medium/high (≥40) decisional regret cohorts. Patient-reported outcome measures (PROMs) included the Oswestry Disability Index (ODI), Patient-Reported Outcomes Measurement Information System (PROMIS), Visual Analog Scale (VAS) Back/Leg/Arm, and Neck Disability Index (NDI) at preoperative, early postoperative (<6 mo), and late postoperative (≥6 mo) timepoints. Differences in demographics, operative variables, and PROMs between low and medium/high decisional regret groups were evaluated.<br><br>Results: A total of 295 patients were included (mean follow-up: 18.2 mo). Overall, 92% of patients agreed that having surgery was the right decision and 90% would make the same decision again. In contrast, 6% of patients regretted the decision to undergo surgery and 7% noted that surgery caused them harm. In-hospital complications (P=0.02) and revision fusion (P=0.026) were significantly associated with higher regret. The medium/high decisional regret group also exhibited significantly worse PROMs at long-term follow-up for all metrics except VAS-Arm, and worse achievement of minimum clinically important difference for ODI (P=0.007), PROMIS (P<0.0001), and VAS-Leg (P<0.0001).<br><br>Conclusion: Higher decisional regret was associated with revision fusion, increased in-hospital complications, and worse PROMs. However, 90% of patients overall were satisfied with their decision to undergo spine surgery for degenerative conditions.
ChatGPT8	Study Design: Prospective cohort study.<br><br>Objective: This study aimed to assess patient satisfaction following spine surgery for degenerative conditions and identify factors associated with satisfaction.<br><br>Summary of Background Data: Patient satisfaction is an important outcome measure in spine surgery. Understanding patient satisfaction rates and the factors influencing it can help inform clinical decision-making and enhance patient-centered care.<br><br>Methods: A prospective cohort of patients undergoing spine surgery for degenerative conditions was recruited from a single tertiary referral center. Patients completed preoperative and postoperative questionnaires, including the Oswestry Disability Index (ODI) and the EuroQol-5D (EQ-5D). Satisfaction with the decision to undergo surgery was assessed using a Likert scale ranging from 1 (very dissatisfied) to 5 (very satisfied). Demographic, clinical, and surgical variables were collected and analyzed to determine their association with patient satisfaction.<br><br>Results: A total of 286 patients were included in the analysis. The majority of patients (90%) reported being satisfied with their decision to undergo spine surgery for degenerative conditions. Mean ODI and EQ-5D scores significantly improved after surgery ($p < 0.001$). Multivariate analysis revealed that younger age, higher preoperative ODI scores, and greater postoperative improvement in ODI scores were associated with higher satisfaction levels ($p < 0.05$). Surgical variables, including the type of procedure and number of levels treated, did not significantly impact patient satisfaction.<br><br>Conclusion: The findings of this prospective cohort study demonstrate that the majority of patients undergoing spine surgery for degenerative conditions are satisfied with their decision. Younger age, higher preoperative disability, and greater postoperative improvement in functional status are associated with higher satisfaction levels. These results highlight the importance of patient-centered care and provide valuable insights for clinical decision-making and patient counseling regarding expected outcomes of spine surgery for degenerative conditions.
Bard8	Background: Degenerative spine conditions are a leading cause of disability in the United States. Surgery is often the only effective treatment for these conditions. However, there is limited data on patient satisfaction with spine surgery.<br><br>Methods: We conducted a retrospective study of patients who underwent spine surgery for degenerative conditions at a single institution. We collected data on patient demographics, clinical characteristics, surgical procedures, and patient-reported outcomes. We used a validated patient-reported satisfaction survey to assess patient satisfaction with their decision to undergo spine surgery.<br><br>Results: A total of 500 patients were included in the study. The average age of the patients was 55 years old. The most common surgical procedures performed were lumbar discectomy (30%), lumbar fusion (25%), and cervical discectomy (20%). The average patient satisfaction score was 90%. There was no significant difference in patient satisfaction between patients who underwent different surgical procedures.<br><br>Conclusions: Our study found that 90% of patients are satisfied with their decision to undergo spine surgery for degenerative conditions. These findings suggest that spine surgery is a safe and effective treatment for degenerative spine conditions and that patients are generally satisfied with the results of surgery.<br><br>Keywords: degenerative spine conditions, surgery, patient satisfaction, retrospective study

Spine Journal 9

Title9	Persistent Use of Prescription Opioids Before and After Lumbar Spine Surgery: Observational Study With Prospectively Collected Data From Two Norwegian National Registries
Abstract9	Study design: Prospective pharmacoepidemiologic study<br><br>Objective: To investigate clinical and sociodemographic factors associated with persistent opioid use in the years following spine surgery among patients with persistent opioid use preceding lumbar spine surgery.<br><br>Summary of background data: It is unknown whether successful spine surgery leads to a cessation of preoperative persistent opioid use.<br><br>Methods: Data from the Norwegian Registry for Spine Surgery and the Norwegian Prescription Database were linked for patients operated for degenerative lumbar spine disorders between 2007 and 2017. The primary outcome measure was persistent opioid use in the second year after surgery. Functional disability was measured with the Oswestry Disability Index (ODI). Factors associated with persistent opioid use in the year before, and two years following, surgery were identified using multivariable logistic regression analysis. The variables included in the analysis were selected based on their demonstrated role in prior studies.<br><br>Results: The prevalence of persistent opioid use was 8.7% in the year before surgery. Approximately two-thirds of patients also met the criteria for persistent opioid use the second year after surgery. Among patients who did not meet the criteria for persistent opioid use the year before surgery, 991 (3.3%) patients developed persistent opioid use in the second year following surgery. The strongest association was exhibited by high doses of benzodiazepines in the year preceding surgery (OR 1.7, 95% CI 1.26 to 2.19, P<0.001). Among patients without persistent opioid use, the most influential factor associated with new-onset persistent opioid use in the second year after surgery was the use of high doses of benzodiazepines (OR 1.8, 95% CI 1.26 to 2.44, P<0.001), high doses of z-hypnotics (OR 2.6, 95% CI 2.10 to 3.23, P<0.001) and previous surgery at the same lumbar level (OR 1.37, 95% CI 1.11 to 1.68, P=0.003).<br><br>Conclusion: A substantial proportion of patients reported sustained opioid use after surgery. Patients with persistent opioid use before surgery should be supported to taper off opioid treatment. Special efforts appear to be required to taper off opioid use in patients using high doses of benzodiazepines.
ChatGPT9	Study Design: Observational study with prospectively collected data.<br><br>Objective: This study aimed to investigate the persistent use of prescription opioids before and after lumbar spine surgery, utilizing data from two Norwegian national registries.<br><br>Summary of Background Data: Prescription opioid use has become a major public health concern worldwide. Lumbar spine surgery is a commonly performed procedure for various spinal disorders, and understanding the patterns of opioid use in this population is essential for optimizing pain management strategies and minimizing long-term opioid dependence.<br><br>Methods: Data from the Norwegian Patient Registry and the Norwegian Prescription Database were analyzed to identify patients who underwent lumbar spine surgery between 2010 and 2018. Patients were categorized based on opioid use patterns, including non-users, intermittent users, and persistent users. Persistent opioid use was defined as having at least one prescription filled within 90 days before surgery and another prescription filled within 180 days after surgery. Demographic and clinical characteristics, surgical procedures, and opioid prescriptions were evaluated.<br><br>Results: A total of 3,246 patients who underwent lumbar spine surgery were included in the study. Among them, 28.5% (n=925) were classified as persistent opioid users. Persistent opioid use was associated with younger age, female gender, higher comorbidity burden, and higher preoperative pain intensity. Furthermore, patients undergoing more extensive surgical procedures, such as fusion or revision surgery, were more likely to be persistent opioid users.<br><br>Conclusion: This observational study provides insight into the persistent use of prescription opioids before and after lumbar spine surgery in Norway. Nearly one-third of patients continued opioid use after surgery, indicating the need for targeted interventions to reduce long-term opioid use in this population. Understanding the demographic and clinical factors associated with persistent opioid use can guide preoperative counseling and personalized pain management strategies to mitigate the risk of chronic opioid dependence following lumbar spine surgery.
Bard9	Background: Opioids are commonly prescribed for chronic pain following lumbar spine surgery. However, the long-term use of opioids is associated with a number of risks, including addiction, overdose, and death.<br><br>Methods: We conducted a retrospective cohort study of patients who underwent lumbar spine surgery in Norway between 2007 and 2017. We linked data from the Norwegian Registry for Spine Surgery and the Norwegian Prescription Database to identify patients who had been prescribed opioids before and after surgery. We used Cox proportional hazards models to assess the association between persistent opioid use and a number of patient- and surgery-related factors.<br><br>Results: A total of 12,500 patients were included in the study. The prevalence of persistent opioid use was 25% at 1 year and 15% at 2 years after surgery. Patients who were younger, had a higher preoperative pain score, and had a more complex surgery were more likely to use opioids persistently.<br><br>Conclusions: Our study found that a significant proportion of patients who undergo lumbar spine surgery continue to use opioids for at least 2 years after surgery. Patients who are younger, have a higher preoperative pain score, and have a more complex surgery are more likely to use opioids persistently. These findings suggest that clinicians should carefully consider the risks and benefits of opioid use before prescribing them to patients who are considering lumbar spine surgery.<br><br>Keywords: lumbar spine surgery, opioids, persistent use, risk factors, observational study

Spine Journal 10

Title10	Interbody Cage Placement Without Plate Supplementation Adjacent to Plated Segments in Multilevel Anterior Cervical Decompression and Fusion
Abstract10	Study Design: A retrospective cohort study<br><br>Objective: To evaluate the clinical efficacy and safety of hybrid anterior cervical fixation, focusing on stand-alone segments<br><br>Summary of Background Data: In the treatment of multilevel cervical stenosis, the number of segments fixed using a plate is limited by placing an interbody cage without plate supplementation at one end of the surgical segment to reduce long plate-related problems. However, the stand-alone segment may experience cage extrusion, subsidence, cervical alignment deterioration, and non-union.<br><br>Methods: Patients who underwent 3-segment or 4-segment fixation for cervical degenerative disease and completed 1-year follow-up were included in this study. Patients were divided into two groups: a cranial group, with stand-alone segments located at the cranial-end adjacent to plated segments, and a caudal group with stand-alone segments located at the caudal-end. Differences in radiographic outcomes between the groups were evaluated. Fusion was defined using dynamic radiographs or computed tomography. To identify factors associated with non-union in stand-alone segments, multivariable logistic regression analyses were performed. To identify factors associated with cage subsidence, multiple regression analyses were performed.<br><br>Results: A total of 116 patients (mean age, 59±11 y; 72% male; mean fixed segments, 3.7±0.5 segments) were included in this study. No case showed cage extrusion or plate dislodgement. In stand-alone segments, the fusion rate was significantly lower in the caudal group than in the cranial group (76% vs. 93%, P=0.019). Change in the cervical sagittal vertical axis was worse in the caudal group than in the cranial group (2.7±12.3 mm vs. −2.7±8.1 mm, P=0.006). One caudal group patient required additional surgery because of non-union at the stand-alone segment. Multivariable logistic regression indicated factors associated with non-union included location of stand-alone segment (caudal-end: OR 4.67, 95%CI, 1.29 to 16.90), larger pre-disc space ROM (OR 1.15, 95%CI, 1.04 to 1.27), and lower pre-operative disc space height (OR 0.57, 95%CI, 0.37 to 0.87). Multiple regression analysis indicated that higher cage height and lower pre-disc space height were associated with cage subsidence.<br><br>Conclusion: Hybrid anterior cervical fixation with stand-alone interbody cage placement adjacent to plated segments may avoid long plate-related problems. Our results suggest that the cranial-end of the construct may be more suitable for the stand-alone segment than the caudal-end.
ChatGPT10	Background Context: Anterior cervical decompression and fusion (ACDF) with plate supplementation is a well-established surgical technique for treating multilevel cervical disc diseases. However, concerns regarding increased rigidity and potential complications associated with the use of additional plates have emerged. This study aimed to investigate the outcomes of interbody cage placement without plate supplementation adjacent to plated segments in multilevel ACDF procedures.<br><br>Purpose: This study aimed to evaluate the clinical and radiographic outcomes, as well as the complication rates, of interbody cage placement without supplemental plating adjacent to plated segments in patients undergoing multilevel ACDF.<br><br>Study Design/Setting: This was a retrospective cohort study conducted at a single institution.<br><br>Methods: A total of 82 patients who underwent multilevel ACDF with interbody cage placement were included in the study. Among them, 42 patients received supplemental plate fixation adjacent to the interbody cages (plate group), while 40 patients underwent interbody cage placement without additional plate supplementation (non-plate group). Clinical outcomes were assessed using the visual analog scale (VAS) for neck and arm pain, the Neck Disability Index (NDI), and the Short Form-36 (SF-36) questionnaire. Radiographic assessments included evaluation of fusion rates and changes in cervical alignment. Complication rates were also recorded.<br><br>Results: Both groups demonstrated significant improvement in clinical outcomes postoperatively. There were no statistically significant differences between the plate and non-plate groups regarding VAS scores for neck pain (p=0.245) and arm pain (p=0.394), NDI scores (p=0.562), and SF-36 scores (p=0.712). Radiographic analysis revealed comparable fusion rates between the two groups (p=0.726). There were no significant differences in cervical alignment changes (p=0.382) or overall complication rates (p=0.682) between the plate and non-plate groups.<br><br>Conclusions: Interbody cage placement without supplemental plate fixation adjacent to plated segments in multilevel ACDF yielded comparable clinical and radiographic outcomes to the traditional approach with plate supplementation. This technique offers a potentially viable alternative that eliminates the need for additional plates, reducing the potential for complications associated with increased rigidity. However, larger prospective studies are warranted to further validate these findings and assess long-term outcomes.
Bard10	Background: Anterior cervical decompression and fusion (ACDF) is a common surgical procedure for the treatment of cervical spondylotic myelopathy (CSM). The use of an anterior cervical plate (ACP) is a common adjunct to ACDF to provide additional stability. However, ACPs can have several drawbacks, including increased risk of complications, such as dysphagia and screw loosening. The purpose of this study was to evaluate the safety and efficacy of interbody cage placement without plate supplementation adjacent to plated segments in multilevel ACDF.<br>Methods: This was a retrospective study of patients who underwent multilevel ACDF with interbody cage placement without plate supplementation adjacent to plated segments at a single academic medical center. Patients were followed for a minimum of 2 years. The primary outcome was fusion rate. Secondary outcomes included complications, patient-reported outcomes, and radiographic parameters.<br>Results: A total of 100 patients were included in the study. The mean age of the patients was 61.2 years, and 75% were female. The mean follow-up was 3.2 years. The fusion rate was 98%. There were no significant complications related to the interbody cages. The mean Oswestry Disability Index (ODI) score decreased from 34.6 to 16.2 at 2 years. The mean Visual Analog Scale (VAS) score for neck pain decreased from 7.2 to 2.8 at 2 years. The mean VAS score for arm pain decreased from 6.4 to 3.2 at 2 years. The mean Cobb angle at the operated levels decreased from 17.2° to 7.8° at 2 years.<br>Conclusions: Interbody cage placement without plate supplementation adjacent to plated segments is a safe and effective treatment for multilevel ACDF. This technique may offer the advantages of decreased risk of complications associated with ACPs, while still providing adequate stability.<br>Keywords: anterior cervical decompression and fusion, interbody cage, plate supplementation, fusion rate, complications, patient-reported outcomes, radiographic parameters

The Spine Journal 1

Title1	Anterolateral versus posterior minimally invasive lumbar interbody fusion surgery for spondylolisthesis: Comparison of outcomes from a global, multicenter study at 12-months follow-up
Abstract1	Background context: Several minimally invasive lumbar interbody fusion techniques may be used as a treatment for spondylolisthesis to alleviate back and leg pain, improve function and provide stability to the spine. Surgeons may choose an anterolateral or posterior approach for the surgery however, there remains a lack of real-world evidence from comparative, prospective studies on effectiveness and safety with relatively large, geographically diverse samples and involving multiple surgical approaches.<br>Purpose: To test the hypothesis that anterolateral and posterior minimally invasive approaches are equally effective in treating patients with spondylolisthesis affecting one or two segments at 3-months follow-up and to report and compare patient reported outcomes and safety profiles between patients at 12-months post-surgery.<br>Design: Prospective, multicenter, international, observational cohort study.<br>Patient sample: Patients with degenerative or isthmic spondylolisthesis who underwent 1- or 2-level minimally invasive lumbar interbody fusion.<br>Outcome measures: Patient reported outcomes assessing disability (ODI), back pain (VAS), leg pain (VAS) and quality of life (EuroQol 5D-3L) at 4-weeks, 3-months and 12-months follow-up; adverse events up to 12-months; and fusion status at 12-months post-surgery using x-ray and/or CT-scan. The primary study outcome is improvement in ODI score at 3-months.<br>Methods: Eligible patients from 26 sites across Europe, Latin America and Asia were consecutively enrolled. Surgeons with experience in minimally invasive lumbar interbody fusion procedures used, according to clinical judgement, either an anterolateral (i.e., ALIF, DLIF, OLIF) or posterior (MIDLF, PLIF, TLIF) approach. Mean improvement in disability (ODI) was compared between groups using ANCOVA with baseline ODI score used as a covariate. Paired t-tests were used to examine change from baseline in PRO for both surgical approaches at each timepoint after surgery. A secondary ANCOVA using a propensity score as a covariate was used to test the robustness of conclusions drawn from the between group comparison.<br>Results: Participants receiving an anterolateral approach (n=114) compared to those receiving a posterior approach (n=112) were younger (56.9 vs 62.0 years, p <.001), more likely to be employed (49.1% vs 25.0%, p<.001), have isthmic spondylolisthesis (38.6% vs 16.1%, p<.001) and less likely to only have central or lateral recess stenosis (44.9% vs 68.4%, p=.004). There were no statistically significant differences between the groups for gender, BMI, tobacco use, duration of conservative care, grade of spondylolisthesis, or the presence of stenosis. At 3-months follow-up there was no difference in the amount of improvement in ODI between the anterolateral and posterior groups (23.2±21.3 vs 25.8±19.5, p=.521). There were no clinically meaningful differences between the groups on mean improvement for back- and leg-pain, disability, or quality of life until the 12-months follow-up. Fusion rates of those assessed (n=158; 70% of the sample), were equivalent between groups [anterolateral, 72/88 (81.8%) fused vs posterior, 61/70 (87.1%) fused; p=.390).<br>Conclusions: Patients with degenerative lumbar disease and spondylolisthesis who underwent minimally invasive lumbar interbody fusion presented statistically significant and clinically meaningful improvements from baseline up to 12-months follow-up. There were no clinically relevant differences between patients operated on using an anterolateral or posterior approach.
ChatGPT1	Background Context: Minimally invasive lumbar interbody fusion (MIS-LIF) has emerged as an effective surgical intervention for patients with symptomatic spondylolisthesis. However, the choice between anterolateral and posterior approaches for MIS-LIF remains a matter of controversy. This study aimed to compare the clinical and radiographic outcomes of anterolateral versus posterior MIS-LIF surgeries in patients with spondylolisthesis.<br><br>Methods: This multicenter study enrolled a global cohort of patients diagnosed with symptomatic spondylolisthesis who underwent either anterolateral or posterior MIS-LIF surgery. Preoperative and 12-month postoperative evaluations included clinical outcomes measured by visual analog scale (VAS) scores for back and leg pain, Oswestry Disability Index (ODI), and patient satisfaction. Radiographic assessments encompassed slip angle measurement, intervertebral disc height, and fusion status.<br><br>Results: A total of 250 patients (125 in each group) were included in the analysis. Both anterolateral and posterior MIS-LIF approaches exhibited significant improvements in VAS scores for back and leg pain, ODI, and patient satisfaction at the 12-month follow-up (p < 0.001). Nevertheless, the anterolateral group demonstrated significantly greater improvements in VAS scores for back pain (p = 0.023) and ODI (p = 0.036) compared to the posterior group. Radiographic evaluation revealed similar enhancements in slip angle and intervertebral disc height for both approaches (p > 0.05). Fusion rates were also comparable between the anterolateral and posterior techniques (90.4% vs. 87.2%, p = 0.352).<br><br>Conclusions: Both anterolateral and posterior MIS-LIF surgeries yielded favorable clinical and radiographic outcomes in the management of spondylolisthesis. However, the anterolateral approach exhibited superior improvements in back pain and functional disability compared to the posterior approach at the 12-month follow-up. Fusion rates were similar between the two approaches. These findings offer valuable insights into surgical decision-making for spondylolisthesis treatment, facilitating individualized treatment strategies based on patient-specific factors.<br><br>Keywords: spondylolisthesis, minimally invasive lumbar interbody fusion, anterolateral approach, posterior approach, clinical outcomes, radiographic assessment.
Bard1	Background: Spondylolisthesis is a common spinal disorder that can cause pain, numbness, and weakness in the legs. Treatment options include conservative care, such as physical therapy and medication, or surgery. Minimally invasive lumbar interbody fusion (MILIF) surgery is a surgical procedure that involves inserting a bone graft between two vertebrae to promote fusion. MILIF surgery can be performed through an anterolateral or posterior approach.<br>Methods: This study was a retrospective, multicenter study of patients with spondylolisthesis who underwent MILIF surgery. Patients were divided into two groups: those who underwent anterolateral MILIF surgery (n = 114) and those who underwent posterior MILIF surgery (n = 112). The primary outcome was fusion rate at 12 months. Secondary outcomes included pain, disability, and quality of life.<br>Results: The fusion rate at 12 months was 81.8% in the anterolateral group and 87.1% in the posterior group. There were no significant differences between the groups in terms of pain, disability, or quality of life at 12 months.<br>Conclusions: This study found that there was no significant difference in fusion rates or clinical outcomes between patients who underwent anterolateral and posterior MILIF surgery for spondylolisthesis at 12 months of follow-up. These findings suggest that either approach may be a safe and effective treatment option for patients with spondylolisthesis.<br>Keywords: spondylolisthesis, minimally invasive lumbar interbody fusion, anterolateral approach, posterior approach, fusion rate, pain, disability, quality of life

The Spine Journal 2

Title2	What is the role of CT-Based Hounsfield Unit assessment in the evaluation of Bone Mineral Density in patients undergoing 1- or 2-level lumbar spinal fusion for degenerative spinal pathologies? – A prospective study
Abstract2	Background context: Computed tomography-based vertebral attenuation values (CT-based HU) have been shown to correlate with T-scores on DEXA scan; and have been acknowledged as an independent factor for predicting fragility fractures. Most patients undergoing lumbar surgeries require CT as part of their pre-operative evaluation.<br>Purpose: The current study was thus planned to evaluate the role of lumbar CT as an opportunistic investigation in determining BMD pre-operatively in patients undergoing lumbar fusion.<br>Study Design: Prospective cohort study<br>Patient sample: Patients older than 45 years, who underwent one- to two-level lumbar (L3-S1 levels) fusions<br>Outcome measures: Comparison of the quantitative assessment of osteoporosis using Hounsfield Units on CT (L1-L5) and mean lumbar T-scores on DEXA (Dual Energy Xray Absorptiometry)<br>Hypothesis: HU on CT is comparable to T-score on DEXA as a suitable modality for the assessment of osteoporosis in patients undergoing 1-2 level lumbar fusion<br>Methods: A prospective cohort study was conducted between January and December 2021. Patients older than 45 years, who underwent one- to two-level lumbar (L3-S1 levels) fusions and had complete clinico-radiological records, were prospectively enrolled. A comparison was drawn between the HU [measured by placing an oval region of interest (ROI) over axial, sagittal and coronal images of lumbar vertebrae] on CT and T-scores on DEXA, and analyzed statistically. The HU values correlating best with normal (group A), osteopenia (B) and osteoporosis (C) categories (classified based on T-scores of lumbar spines) were determined statistically.<br>Results: Overall, 87 patients [mean age of 60.56±11.63 years; 63 (72.4%) female patients] were prospectively studied. There was a statistically significant difference in the mean age (p=0.01) and sex distribution (predominantly female patients; p=0.03) of patients belonging to groups B (osteopenic) and C (osteoporotic patients), as compared with group A.<br>The greatest correlation between T-score (on DEXA) and HU (on CT) for differentiating osteopenia (group B) from group A was observed at levels L1 (p<0.001), L2 (p<0.001) and L3 (p<0.001). Based on receiver-operating characteristic (ROC) curve analysis, the cut-off values for HU for identifying osteopenia were 159 (at L1; sensitivity 81.6 and specificity 80) and 162 (at L2; sensitivity 80 and specificity 71.1). In addition, there was statistically significant correlation between T-score (on DEXA) and HU at all the lumbar levels for distinguishing osteoporosis (group C), although the difference was most evident at the upper lumbar (L1 and L2) levels (p<0.001). Based on ROC analysis, cut-off HU values for defining osteoporosis were 127 (at L1; sensitivity 71.3 and specificity 70) and 117 (at L2; sensitivity 65.5 and specificity 90).<br>Conclusion: Based on our study, the measurement of HU on CT at upper lumbar levels can be considered as "surrogate marker" for BMD in the diagnosis of osteopenia (cut-off: 159 at L1, 162 at L2) and osteoporosis (cut-off: 127 at L1, 117 at L2) in patients undergoing lumbar fusion surgeries. The HU measurements on CT at the lower lumbar levels (L4 and L5) are less reliable in this pre-operative scenario.
ChatGPT2	Background Context: Lumbar spinal fusion is a common surgical intervention for degenerative spinal pathologies. The success of the fusion process relies on the achievement of adequate bone healing and stability. Bone mineral density (BMD) is a crucial factor that influences fusion outcomes. Currently, dual-energy X-ray absorptiometry (DXA) is the gold standard for assessing BMD; however, its limitations in measuring the bone quality at the fusion site have prompted the exploration of alternative modalities. Computed tomography (CT) provides an opportunity to evaluate BMD through the quantification of Hounsfield Units (HU). This study aims to investigate the role of CT-based HU assessment in the evaluation of BMD in patients undergoing 1- or 2-level lumbar spinal fusion.<br>Purpose: This prospective study aims to assess the utility of CT-based HU assessment as a complementary tool to DXA for evaluating BMD at the fusion site in patients undergoing 1- or 2-level lumbar spinal fusion for degenerative spinal pathologies.<br>Study Design: A prospective observational study was conducted involving patients who underwent 1- or 2-level lumbar spinal fusion for degenerative spinal pathologies. DXA and CT scans were performed preoperatively and at specific follow-up intervals. HUs were measured within the fusion segment on CT scans, and BMD was measured using DXA at the lumbar spine. The correlation between HU and DXA measurements was analyzed, and the predictive value of HU for fusion outcomes was assessed.<br>Methods: A total of 100 patients (63 males and 37 females, mean age 56.8 years) were included in the study. Preoperative and postoperative DXA and CT scans were performed at predetermined intervals (3, 6, and 12 months postoperatively). HU measurements were obtained using CT scans at the fusion level, and BMD was measured using DXA at the lumbar spine. Statistical analyses, including correlation coefficients, regression analyses, and receiver operating characteristic (ROC) curve analysis, were conducted to evaluate the relationship between HU and DXA measurements and their predictive value for fusion outcomes.<br>Results: The results demonstrated a significant correlation between HU and DXA measurements (p < 0.001). HU values exhibited a positive association with BMD, indicating that higher HU values corresponded to higher BMD levels. Furthermore, HU measurements were found to be a significant predictor of fusion outcomes, with higher HU values associated with improved fusion success rates. The ROC curve analysis indicated that HU measurements had a high sensitivity and specificity for predicting fusion outcomes (area under the curve = 0.86).<br>Conclusion: CT-based HU assessment provides valuable information about BMD at the fusion site in patients undergoing 1- or 2-level lumbar spinal fusion for degenerative spinal pathologies. The correlation between HU and DXA measurements, as well as the predictive value of HU for fusion outcomes, suggests that HU assessment can serve as a complementary tool to DXA in evaluating BMD and predicting fusion success. Incorporating CT-based HU assessment into routine clinical practice may enhance the preoperative evaluation and postoperative monitoring of lumbar spinal fusion patients, ultimately leading to improved surgical outcomes and patient care. Further research is warranted to validate these findings and explore the potential of CT-based HU assessment in larger patient populations.
Bard2	Background: Low bone mineral density (BMD) is a risk factor for screw loosening after lumbar spinal fusion. Computed tomography (CT)-based Hounsfield unit (HU) assessment is a potential method for evaluating BMD.<br>Methods: We conducted a prospective study of 100 patients who underwent 1- or 2-level lumbar spinal fusion for degenerative spinal pathologies. BMD was assessed preoperatively and postoperatively using CT-based HU and dual-energy X-ray absorptiometry (DEXA). Screw loosening was defined as a change in screw position of >2 mm or a screw tip that was not in contact with bone.<br>Results: There was a significant correlation between preoperative CT-based HU and postoperative BMD (r=0.86, p<0.001). Patients with preoperative CT-based HU values <140 had a significantly higher risk of screw loosening (odds ratio [OR] 4.5, 95% confidence interval [CI] 1.5-13.8) than patients with preoperative CT-based HU values ≥140.<br>Conclusions: CT-based HU assessment can be used to predict the risk of screw loosening after lumbar spinal fusion. Patients with low preoperative CT-based HU values may benefit from additional measures to improve bone strength, such as bisphosphonate therapy or bone grafting.

The Spine Journal 3

Title3	Machine learning-based algorithms to predict severe psychological distress among cancer patients with spinal metastatic disease
Abstract3	BACKGROUND CONTEXT: Metastatic spinal disease is an advanced stage of cancer patients and often suffer from terrible psychological health status; however, the ability to estimate the risk probability of this adverse outcome using current available data is very limited.<br>PURPOSE: The goal of this study was to propose a precise model based on machine learning techniques to predict psychological status among cancer patients with spinal metastatic disease.<br>STUDY DESIGN/SETTING: A prospective cohort study.<br>PATIENT SAMPLE: A total of 1043 cancer patients with spinal metastatic disease were included.<br>OUTCOME MEASURES: The main outcome was severe psychological distress.<br>METHODS: The total of patients was randomly divided into a training dataset and a testing dataset on a ratio of 9:1. Patient's demographics, lifestyle choices, cancer-related features, clinical manifestations, and treatments were collected as potential model predictors in the study. Five machine learning algorithms, including XGBoosting machine, random forest, gradient boosting machine, support vector machine, and ensemble prediction model, as well as a logistic regression model were employed to train and optimize models in the training set, and their predictive performance was assessed in the testing set.<br>RESULTS: Up to 21.48% of all patients who were recruited had severe psychological distress. Elderly patients (p<0.001), female (p =0.045), current smoking (p=0.002) or drinking (p=0.003), a lower level of education (p<0.001), a stronger spiritual desire (p<0.001), visceral metastasis (p=0.005), and a higher Eastern Cooperative Oncology Group (ECOG) score (p<0.001) were significantly associated with worse psychological health. With an area under the curve (AUC) of 0.865 (95% CI: 0.788–0.941) and an accuracy of up to 0.843, the gradient boosting machine algorithm performed best in the prediction of the outcome, followed by the XGBooting machine algorithm (AUC: 0.851, 95% CI: 0.768–0.934; Accuracy: 0.826) and ensemble prediction (AUC: 0.851, 95% CI: 0.770–0.932; Accuracy: 0.809) in the testing set. In contrast, the AUC of the logistic regression model was only 0.836 (95% CI: 0.756–0.916; Accuracy: 0.783).<br>CONCLUSIONS: Machine learning models have greater predictive power and can offer useful tools to identify individuals with spinal metastatic disease who are experiencing severe psychological distress.
ChatGPT3	Background Context: Cancer patients with spinal metastatic disease often experience severe psychological distress, which can significantly impact their quality of life and treatment outcomes. Early identification of patients at high risk for psychological distress is crucial for providing timely interventions and support. Machine learning algorithms have shown promise in predicting psychological outcomes in various medical conditions. However, their application in predicting severe psychological distress among cancer patients with spinal metastatic disease remains unexplored.<br><br>Purpose: This study aims to develop and validate machine learning-based algorithms to predict severe psychological distress among cancer patients with spinal metastatic disease, thereby facilitating early identification and targeted interventions.<br><br>Study Design: A retrospective cohort study was conducted using data collected from cancer patients diagnosed with spinal metastatic disease. Demographic, clinical, and psychological variables were collected at baseline. Patients' psychological distress levels were assessed using standardized questionnaires, such as the Hospital Anxiety and Depression Scale (HADS) and the Distress Thermometer (DT). Machine learning algorithms, including random forest, support vector machines, and logistic regression, were trained and evaluated to predict severe psychological distress.<br><br>Methods: The study included 250 cancer patients (mean age 57.2 years, 53% males) with spinal metastatic disease. Psychological distress was classified as severe or non-severe based on HADS and DT scores. Demographic and clinical variables, including age, gender, cancer type, previous psychiatric history, pain levels, functional status, and treatment modality, were used as input features for the machine learning algorithms. The algorithms were trained using a subset of the data and evaluated using cross-validation techniques. Performance measures, including accuracy, sensitivity, specificity, and area under the receiver operating characteristic curve (AUC), were calculated to assess the predictive accuracy of the algorithms.<br><br>Results: The machine learning algorithms demonstrated good predictive performance for severe psychological distress among cancer patients with spinal metastatic disease. The random forest algorithm achieved the highest accuracy (82%), sensitivity (80%), specificity (84%), and AUC (0.85). Important predictors identified by the algorithms included previous psychiatric history, pain levels, and functional status. The algorithms provided valuable insights into the relative importance of different variables in predicting severe psychological distress.<br><br>Conclusion: Machine learning-based algorithms show promise in accurately predicting severe psychological distress among cancer patients with spinal metastatic disease. These algorithms can aid in the early identification of patients at high risk for psychological distress, enabling timely interventions and support. The incorporation of machine learning algorithms into routine clinical practice may improve patient care by facilitating personalized psychological care plans for cancer patients with spinal metastatic disease. Further research is warranted to validate the findings in larger patient populations and to assess the feasibility of implementing these algorithms in clinical settings.
Bard3	Background: Spinal metastatic disease is a common and often debilitating complication of cancer. Patients with spinal metastatic disease are at increased risk for psychological distress, which can have a significant impact on their quality of life.<br><br>Methods: We conducted a prospective cohort study of 1043 patients with spinal metastatic disease. We used five machine learning algorithms to predict severe psychological distress, defined as a score of 14 or higher on the Hospital Anxiety and Depression Scale (HADS).<br><br>Results: The gradient boosting machine algorithm had the highest area under the curve (AUC) for predicting severe psychological distress (AUC = 0.865). The XGBoosting machine algorithm had an AUC of 0.851, the support vector machine had an AUC of 0.843, the random forest algorithm had an AUC of 0.836, and the ensemble prediction model had an AUC of 0.851.<br><br>Conclusions: Machine learning algorithms can be used to predict severe psychological distress in patients with spinal metastatic disease. These algorithms could be used to identify patients who are at risk for psychological distress and to provide them with early intervention.

The Spine Journal 4

Title4	Dyslipidemia as a novel risk for the development of symptomatic ossification of the posterior longitudinal ligament
Abstract4	BACKGROUND CONTEXT: Obesity and visceral fat have been implicated as potential factors in the pathogenesis of the ossification of the posterior longitudinal ligament (OPLL); the details of the factors involved in OPLL remain unclear.<br>PURPOSE: We aimed to determine the association between dyslipidemia and symptomatic OPLL.<br>STUDY DESIGN: Single institution cross-sectional study.<br>PATIENT SAMPLE: Data were collected from Japanese patients with OPLL (n=92) who underwent whole-spine computed tomography scanning. Control data (n=246) without any spinal ligament ossification were collected from 627 Japanese participants who underwent physical examination.<br>OUTCOME MEASURES: Baseline information and lipid parameters, including triglycerides (TG), high-density lipoprotein cholesterol (HDL-C), and low-density lipoprotein cholesterol (LDL-C) from fasting blood samples were collected to assess the comorbidity of dyslipidemia.<br>METHODS: Patient data were collected from 2020 to 2022. Patients with dyslipidemia were defined as those who were taking medication for dyslipidemia and who met one of the following criteria: TG ≥150 mg/dL, LDL-C ≥140 mg/dL, and/or HDL-C <40 mg/dL. The factors associated with OPLL development were evaluated using multivariate logistic regression analysis.<br>RESULTS: The comorbidity of dyslipidemia in the OPLL group was more than twice that in the control group (71.7% and 35.4%, respectively). The mean body mass index (BMI) of the OPLL group was significantly higher than that of the control group (27.2 kg/m2 and 23.0 kg/m2). Multivariate logistic regression analysis revealed that dyslipidemia was associated with the development of OPLL (regression coefficient, 0.80; 95% confidence interval, 0.11–1.50). Additional risk factors included age, BMI, and diabetes mellitus.<br>CONCLUSIONS: We demonstrated a novel association between dyslipidemia and symptomatic OPLL development using serum data. This suggests that visceral fat obesity or abnormal lipid metabolism are associated with the mechanisms of onset and exacerbation of OPLL as well as focal mechanical irritation due to being overweight.
ChatGPT4	Background Context: Symptomatic ossification of the posterior longitudinal ligament (OPLL) is a progressive spinal disorder characterized by the ectopic ossification of the posterior longitudinal ligament. The pathogenesis of OPLL remains incompletely understood, and identifying novel risk factors is crucial for early detection and intervention. Dyslipidemia, a metabolic disorder characterized by abnormal lipid profiles, has been associated with various systemic diseases. However, its relationship with OPLL development has not been extensively explored.<br><br>Purpose: This study aims to investigate the association between dyslipidemia and the development of symptomatic OPLL, shedding light on dyslipidemia as a novel risk factor for OPLL and its potential implications for preventive strategies.<br><br>Study Design: A retrospective cohort study was conducted using a database of patients diagnosed with symptomatic OPLL. The lipid profiles of the patients were collected and compared with a control group without OPLL. Statistical analyses were performed to assess the association between dyslipidemia and OPLL development, considering other confounding factors.<br><br>Methods: A total of 300 patients with symptomatic OPLL (mean age 58.3 years, 58% males) were included in the study, along with a control group of 300 individuals without OPLL, matched for age and gender. Fasting lipid profiles, including total cholesterol, triglycerides, low-density lipoprotein cholesterol (LDL-C), high-density lipoprotein cholesterol (HDL-C), and non-HDL-C, were obtained from medical records. The presence of dyslipidemia was defined according to established guidelines. Logistic regression analyses were performed to assess the association between dyslipidemia and the development of symptomatic OPLL, adjusting for potential confounding factors, such as age, gender, body mass index, smoking status, and comorbidities.<br><br>Results: The presence of dyslipidemia was significantly higher in the symptomatic OPLL group compared to the control group (p < 0.001). Logistic regression analyses demonstrated a significant association between dyslipidemia and the development of symptomatic OPLL (odds ratio = 2.85, 95% confidence interval: 1.89-4.30, p < 0.001). Specifically, elevated LDL-C levels and low HDL-C levels were independently associated with an increased risk of symptomatic OPLL development. The association remained significant even after adjusting for potential confounding factors.<br><br>Conclusion: This study provides evidence for dyslipidemia as a novel risk factor for the development of symptomatic OPLL. Elevated LDL-C levels and low HDL-C levels were identified as independent risk factors. These findings highlight the importance of lipid metabolism in the pathogenesis of OPLL and suggest dyslipidemia as a potential target for preventive strategies. Early identification and management of dyslipidemia may play a role in preventing or delaying the onset of symptomatic OPLL. Further prospective studies are warranted to validate these findings, elucidate the underlying mechanisms, and explore the potential of lipid-lowering interventions in OPLL prevention and management.
Bard4	Background: Ossification of the posterior longitudinal ligament (OPLL) is a common and disabling condition that can lead to spinal cord compression and neurological deficits. The pathogenesis of OPLL is not fully understood, but it is thought to be multifactorial, involving genetic, environmental, and lifestyle factors.<br><br>Methods: We conducted a retrospective cohort study of 92 patients with OPLL and 246 control subjects without OPLL. We collected data on demographic, clinical, and laboratory variables. We used multivariate logistic regression analysis to assess the association between dyslipidemia and OPLL.<br><br>Results: The comorbidity of dyslipidemia was more than twice as high in the OPLL group as in the control group (71.7% vs. 35.4%). The mean body mass index (BMI) was also significantly higher in the OPLL group (27.2 kg/m2 vs. 23.0 kg/m2). Multivariate logistic regression analysis revealed that dyslipidemia was associated with OPLL development (odds ratio [OR], 2.3; 95% confidence interval [CI], 1.1-5.3). Additional risk factors for OPLL included age, BMI, and diabetes mellitus.<br><br>Conclusions: Our study provides novel evidence that dyslipidemia is a risk factor for the development of OPLL. This finding suggests that dyslipidemia may be a target for preventive strategies in patients at risk for OPLL.

The Spine Journal 5

Title5	Determining minimal clinically important difference estimates following surgery for degenerative conditions of the lumbar spine: Analysis of the Canadian Spine Outcomes and Research Network (CSORN) registry
Abstract5	Background Context: There is significant variability in minimal clinically important difference (MCID) criteria for lumbar spine surgery that suggests population and primary pathology specific thresholds may be required to help determine surgical success when using patient reported outcome measures (PROMs).<br>Purpose: The purpose of this study was to estimate MCID thresholds for three commonly used PROMs after surgical intervention for each of four common lumbar spine pathologies.<br>Study Design/Setting: Observational longitudinal study of patients from {BLINDED} national registry.<br>Patient Sample: Patients undergoing surgery from 2015-2018 for lumbar spinal stenosis (LSS; n=856), degenerative spondylolisthesis (DS; n=591), disc herniation (DH; n=520) or degenerative disc disease (DDD n=185) were included.<br>Outcome Measures: PROMs were collected pre-surgery and one-year post-surgery: the Oswestry Disability Index (ODI), and back and leg Numeric Pain Rating Scales (NPRS). At 1-year, patients reported whether they were 'Much better'/'Better'/'Same'/'Worse'/'Much worse' compared to before their surgery. Responses to this item were used as the anchor in analyses to determine surgical MCIDs for benefit ('Much better'/'Better') and substantial benefit ('Much better').<br>Methods: MCIDs for absolute and percentage change for each of the three PROMs were estimated using a receiving operating curve (ROC) approach, with maximization of Youden's index as primary criterion. Area under the curve (AUC) estimates, sensitivity, specificity and correct classification rates were determined. All analyses were conducted separately by pathology group.<br>Results: MCIDs for ODI change ranged from -10.0 (DDD) to -16.9 (DH) for benefit, and -13.8 (LSS) to -22.0 (DS,DH) for substantial benefit. MCID for back and leg NPRS change were -2 to -3 for each group for benefit and -4.0 for substantial benefit for all groups on back NPRS. MCID estimates for percentage change varied by PROM and pathology group, ranging from -11.1% (ODI for DDD) to -50.0% (leg NPRS for DH) for benefit and from -40.0% (ODI for DDD) to -66.6% (leg NPRS for DH) for substantial benefit. Correct classification rates for all MCID thresholds ranged from 71%-89% and were relatively lower for absolute vs. percent change for those with high or low pre-surgical scores.<br>Conclusions: Our findings suggest that the use of generic MCID thresholds across pathologies in lumbar spine surgery is not recommended. For patients with relatively low or high pre-surgery PROM scores, MCIDs based on percentage change, rather than absolute change, appear generally preferable. These findings have applicability in clinical and research settings, and are important for future surgical prognostic work.
ChatGPT5	Background Context: The assessment of minimal clinically important difference (MCID) is essential in evaluating the effectiveness of surgical interventions for degenerative conditions of the lumbar spine. However, there is a lack of consensus regarding MCID estimates for various outcome measures in this patient population. This study aimed to determine MCID estimates following surgery for degenerative lumbar spine conditions using data from the Canadian Spine Outcomes Research Network (CSORN) database.<br><br>Purpose: To establish MCID estimates for commonly used outcome measures following surgical treatment of degenerative lumbar spine conditions and to provide clinicians and researchers with valuable benchmarks for assessing the clinical significance of treatment outcomes.<br><br>Study Design: Retrospective analysis of prospectively collected data.<br><br>Methods: Data from the CSORN database were analyzed to identify patients who underwent surgery for degenerative conditions of the lumbar spine between 2010 and 2020. Several outcome measures, including the visual analog scale (VAS) for back and leg pain, Oswestry Disability Index (ODI), and EuroQol-5 Dimension (EQ-5D) index, were assessed preoperatively and at various postoperative time points. MCID estimates were determined using anchor-based and distribution-based methods, including patient-reported improvement and effect size calculations.<br><br>Results: A total of 1,500 patients were included in the analysis. The MCID estimates for the VAS back pain score ranged from 1.0 to 2.0 points, while those for the VAS leg pain score ranged from 1.5 to 2.5 points. The MCID estimate for the ODI was 10.0 points, indicating a clinically meaningful improvement in functional disability. The MCID estimate for the EQ-5D index was 0.05, reflecting a noticeable change in health-related quality of life. The MCID estimates were consistent across various time points, surgical procedures, and patient demographics.<br><br>Conclusion: This study provides robust MCID estimates for commonly used outcome measures following surgery for degenerative conditions of the lumbar spine. These estimates serve as valuable benchmarks for interpreting treatment outcomes and facilitating evidence-based decision-making in clinical practice. Clinicians and researchers can utilize these MCID values to assess the clinical significance of changes in pain intensity, functional disability, and health-related quality of life following surgical interventions. Future studies should aim to validate these MCID estimates in different patient populations and treatment settings.
Bard5	Background: Degenerative conditions of the lumbar spine are a common cause of pain and disability. Surgical intervention is often recommended for patients who have failed conservative treatment. However, there is no consensus on the minimal clinically important difference (MCID) for patient-reported outcome measures (PROMs) following lumbar spine surgery.<br><br>Methods: We conducted a retrospective cohort study using data from the Canadian Spine Outcomes and Research Network (CSORN) registry. Patients who underwent surgery for degenerative conditions of the lumbar spine between 2015 and 2018 were included. The primary outcome was the MCID for change in Oswestry Disability Index (ODI) score at 1 year after surgery. Secondary outcomes included the MCID for change in back and leg pain scores on the Numeric Pain Rating Scale (NPRS).<br><br>Results: A total of 2052 patients were included in the study. The MCID for ODI change was -10.0 points for lumbar spinal stenosis (LSS), -16.9 points for disc herniation (DH), and -13.8 points for degenerative disc disease (DDD). The MCID for back and leg pain score change was -2 points for LSS, -3 points for DH, and -2 points for DDD.<br><br>Conclusions: Our findings suggest that the MCID for change in ODI score following lumbar spine surgery is -10.0 points for LSS, -16.9 points for DH, and -13.8 points for DDD. The MCID for change in back and leg pain score is -2 points for LSS, -3 points for DH, and -2 points for DDD. These findings can be used to help clinicians and patients make informed decisions about the benefits of surgery for degenerative conditions of the lumbar spine.

The Spine Journal 6

Title6	The Effect of Pedicle Screw Instrumentation at a Young Age on Upper Thoracic Vertebra and Canal Development
Abstract6	Background Context: Pedicle screws are widely used in spinal surgeries. Pedicle screw fixation has shown better clinical effects than other techniques by providing steady fixation from the posterior arch to the vertebral body. However, there are several concerns about the impact of PSI insertion on vertebral development in young children, including early closure of the neurocentral cartilage (NCC). The effect of pedicle screw insertion in an early age on further growth of the upper thoracic spine is still unclear.<br>Purpose: This study aimed to evaluate the impact of pedicle screw insertion on further growth of the upper thoracic vertebra and spinal canal.<br>Study Design: A retrospective case study<br>Patient Sample: Twenty-eight patients.<br>Outcome Measurements: X-ray and CT parameters including length, height and area of the vertebrae and spinal canal were manually measured.<br>Methods: Twenty-eight patients who underwent pedicle screw fixation (T1-T6) before the age of five years from March 2005 to August 2019 at Peking Union Medical College Hospital were recruited, and records were retrospectively reviewed. Vertebral body and spinal canal parameters were measured at instrumented and adjacent noninstrumented levels and compared using statistical methods.<br>Results: Ninety-seven segments met the inclusion criteria (average age at instrumentation 44.57 months, range from 23–60 months). Thirty-nine segments had no screws, and 58 had at least one screw. There was no significant difference between the preoperative and final follow-up values of the measurement of vertebral body parameters. No significant difference was observed between the growth rates in levels with or without screws in pedicle length, vertebral body diameter, or spinal canal parameters.<br>Conclusion: Pedicle screw instrumentation in the upper thoracic spine does not cause a negative effect on the development of the vertebral body and spinal canal in children younger than five years old.
ChatGPT6	Background Context: Pedicle screw instrumentation is a commonly employed technique for spinal stabilization in young patients with various spinal pathologies. However, the impact of pedicle screw instrumentation on the development of upper thoracic vertebrae and the spinal canal remains unclear. This study aimed to investigate the effect of pedicle screw instrumentation at a young age on the development of upper thoracic vertebrae and the spinal canal.<br><br>Purpose: To assess the morphological changes in upper thoracic vertebrae and the spinal canal following pedicle screw instrumentation in young patients, providing insights into the potential implications for spinal growth and development.<br><br>Study Design: Retrospective radiographic analysis.<br><br>Methods: A retrospective analysis was conducted on the radiographic data of patients who underwent pedicle screw instrumentation in the upper thoracic spine at a young age. Serial radiographs, including anteroposterior and lateral views, were assessed to evaluate the changes in upper thoracic vertebral body height, pedicle width, and spinal canal dimensions. Measurements were performed preoperatively, immediately postoperatively, and at follow-up time points.<br><br>Results: A total of 50 patients were included in the study. The analysis revealed a statistically significant reduction in upper thoracic vertebral body height following pedicle screw instrumentation ($p < 0.001$). Moreover, the pedicle width was found to be significantly decreased ($p < 0.001$). However, there were no significant changes in the dimensions of the spinal canal ($p > 0.05$).<br><br>Conclusion: Pedicle screw instrumentation at a young age in the upper thoracic spine was associated with a reduction in vertebral body height and pedicle width. However, no significant alterations were observed in the dimensions of the spinal canal. These findings suggest that pedicle screw instrumentation may have a localized effect on upper thoracic vertebrae without compromising the overall dimensions of the spinal canal. Long-term follow-up studies are needed to determine the clinical implications of these morphological changes and their impact on spinal growth and function.
Bard6	Background: Pedicle screw instrumentation (PSI) is a common surgical procedure used to stabilize the spine in children and adolescents. However, there is concern that PSI may have a negative impact on vertebral development.<br>Methods: We conducted a retrospective study of 28 patients who underwent PSI for spinal deformity or trauma before the age of five years. We measured the length, height, and area of the vertebral bodies and spinal canal at the instrumented and adjacent noninstrumented levels at baseline and at final follow-up.<br>Results: There was no significant difference in the preoperative and final follow-up measurements of vertebral body length, height, or area at the instrumented or adjacent noninstrumented levels. There was also no significant difference in the growth rates of the vertebral bodies or spinal canal at the instrumented or adjacent noninstrumented levels.<br>Conclusions: Our findings suggest that PSI does not have a negative impact on the development of the upper thoracic vertebrae or spinal canal in children younger than five years old.

The Spine Journal 7

Title7	Treatment of Adult Deformity Surgery by Orthopedic and Neurological Surgeons: Trends in Treatment, Techniques, and Costs by Specialty
Abstract7	Background Context: Surgery to correct adult spinal deformity (ASD) is performed by both neurological surgeons and orthopedic surgeons. Despite well-documented high costs and complication rates following ASD surgery, there is a dearth of research investigating trends in treatment according to surgeon subspeciality.<br>Purpose: The purpose of this investigation was to perform an analysis of surgical trends, costs and complications of ASD operations by physician specialty using a large, nationwide sample.<br>Study Design/Setting: Retrospective cohort study using an administrative claims database.<br>Patient Sample: A total of 12,929 patients were identified with ASD that underwent deformity surgery performed by neurological or orthopedic surgeons.<br>Outcome Measures: The primary outcome was surgical case volume by surgeon specialty. Secondary outcomes included costs, medical complications, surgical complications, and reoperation rates (30-day, 1-year, 5-year, and total).<br>Methods: The PearlDiver Mariner database was queried to identify patients who underwent ASD correction from 2010-2019. The cohort was stratified to identify patients who were treated by either orthopedic or neurological surgeons. Surgical volume, baseline characteristics, and surgical techniques were examined between cohorts. Multivariable logistic regression was employed to assess the cost, rate of reoperation and complication according to each subspecialty while controlling for number of levels fused, rate of pelvic fixation, age, gender, region and Charlson Comorbidity Index (CCI). Alpha was set to 0.05 and a Bonferroni correction for multiple comparisons was utilized to set the significance threshold at p ≤0.000521.<br>Results: A total of 12,929 ASD patients underwent deformity surgery performed by neurological or orthopedic surgeons. Orthopedic surgeons performed most deformity procedures accounting for 64.57% (8,866/12,929) of all ASD operations, while the proportion treated by neurological surgeons increased 44.2% over the decade (2010: 24.39% vs 2019: 35.16%; p<0.0005). Neurological surgeons more frequently operated on older patients (60.52 vs 55.18 years, p<0.0005) with more medical comorbidities (CCI scores: 2.01 vs 1.47, p<0.0005). Neurological surgeons also performed higher rates of arthrodesis between 1-6 levels (OR: 1.86, p<0.0005), 3 column osteotomies (OR: 1.35, p<0.0005) and navigated or robotic procedures (OR: 3.30, p<0.0005). Procedures performed by orthopedic surgeons had significantly lower average costs as compared to neurological surgeons (Orthopedic Surgeons: $17,971.66 vs Neurological Surgeons: $22,322.64, p=0.253). Adjusted logistic regression controlling for number of levels fused, pelvic fixation, age, sex, region, and comorbidities revealed that patients within neurosurgical care had similar odds of complications to orthopaedic surgery.<br>Conclusions: This investigation of over 12,000 ASD patients demonstrates orthopedic surgeons continue to perform the majority of ASD correction surgery, although neurological surgeons are performing an increasingly larger percentage over time with a 44% increase in the proportion of surgeries performed in the decade. In this cohort, neurological surgeons more frequently operated on older and more comorbid patients, utilizing shorter-segment fixation with greater use of navigation and robotic assistance.
ChatGPT7	Background Context: Adult deformity surgery is a complex and demanding field that requires specialized knowledge and surgical skills. Both orthopedic and neurological surgeons have been involved in the management of adult spinal deformities; however, there is a scarcity of literature comparing the treatment approaches, surgical techniques, and costs between these specialties. This study aimed to investigate the trends in treatment, surgical techniques, and costs associated with adult deformity surgery, comparing orthopedic and neurological surgeons.<br><br>Purpose: To analyze and compare the treatment patterns, surgical techniques, and costs of adult deformity surgery performed by orthopedic and neurological surgeons, providing valuable insights into the differences and potential implications for patient outcomes and healthcare resource utilization.<br><br>Study Design: Retrospective analysis of a national surgical database.<br><br>Methods: Data from a national surgical database were analyzed to identify adult patients who underwent deformity surgery performed by either orthopedic or neurological surgeons. Patient demographics, surgical techniques, perioperative variables, and healthcare costs were assessed. Statistical analysis was performed to compare the treatment approaches, surgical techniques, and costs between the two specialties.<br><br>Results: A total of 500 patients were included in the analysis, with 250 patients treated by orthopedic surgeons and 250 patients treated by neurological surgeons. Significant variations were observed in the treatment patterns and surgical techniques employed by the two specialties. Orthopedic surgeons were more likely to utilize posterior-based approaches (p < 0.001), while neurological surgeons favored combined anterior-posterior approaches (p < 0.001). Additionally, significant differences were noted in the utilization of specific instrumentation and fusion techniques. Healthcare costs were higher for surgeries performed by neurological surgeons compared to orthopedic surgeons (p < 0.001).<br><br>Conclusion: This study highlights the differences in treatment patterns, surgical techniques, and costs between orthopedic and neurological surgeons in the management of adult spinal deformities. The choice of surgical approach and techniques varied significantly between the two specialties, potentially influencing patient outcomes and resource utilization. These findings underscore the importance of interdisciplinary collaboration and shared decision-making in optimizing patient care for adult deformity surgery. Further research is warranted to investigate the long-term outcomes and cost-effectiveness of these different treatment approaches.
Bard7	Background: Adult spinal deformity (ASD) is a common condition that can cause pain, disability, and decreased quality of life. Surgery is often the only effective treatment for ASD. Orthopedic and neurological surgeons both perform ASD surgery, but there is a lack of data on how these two specialties differ in their treatment approaches.<br>Methods: We conducted a retrospective study of 12,929 patients who underwent ASD surgery in the United States from 2010 to 2019. We collected data on patient demographics, surgical techniques, complications, and costs. We used multivariable logistic regression to assess the association between surgeon specialty and treatment outcomes.<br>Results: Orthopedic surgeons performed most ASD surgeries (64.57%), followed by neurological surgeons (35.16%). Orthopedic surgeons were more likely to use anterior approaches to the spine (56.39%) than neurological surgeons (38.52%). Orthopedic surgeons were also more likely to use pedicle screws (89.64%) than neurological surgeons (78.33%). There was no significant difference in the rate of complications between the two specialties. However, orthopedic surgery was associated with a higher cost than neurological surgery ($135,000 vs $115,000).<br>Conclusions: Orthopedic surgeons are more likely to use anterior approaches and pedicle screws than neurological surgeons for ASD surgery. Orthopedic surgery is also associated with a higher cost than neurological surgery. These findings suggest that there are important differences in the treatment approaches of orthopedic and neurological surgeons for ASD.

The Spine Journal 8

Title8	Evolution of patient-reported outcome measures, 1, 2, and 5 years after surgery for subaxial cervical spine fractures, a nation-wide registry study
Abstract8	BACKGROUND CONTEXT: A longer duration of patient follow-up arguably provides more reliable data on the long-term effects of a treatment. However, the collection of long-term follow-up data is resource demanding and often complicated by missing data and patients being lost to follow-up. In surgical fixation for cervical spine fractures, data are lacking on the evolution of patient reported outcome measures (PROMs) beyond 1-year of follow-up. We hypothesized that the PROMs would remain stable beyond the 1-year postoperative follow-up mark, regardless of the surgical approach.<br>PURPOSE: To assess the trends in the evolution of patient-reported outcome measures (PROMs) at 1, 2-, and 5-years following surgery in patients with traumatic cervical spine injuries.<br>STUDY DESIGN: Nation-wide observational study on prospectively collected data.<br>PATIENT SAMPLE: Individuals treated for subaxial cervical spine fractures with anterior, posterior, or combined anteroposterior approaches, between 2006 and 2016 were identified in the Swedish Spine Registry (Swespine).<br>OUTCOME MEASURES: PROMs consisting of EQ-5D-3Lindex and the Neck Disability Index (NDI) were considered.<br>METHODS: PROMs data were available for 292 patients at 1 and 2 years postoperatively. Five-years PROMs data were available for 142 of these patients. A simultaneous within-group (longitudinal) and between group (approach-dependent) analysis was performed using mixed ANOVA. The predictive ability of 1-year PROMs was subsequently assessed using linear regression.<br>RESULTS: Mixed ANOVA revealed that PROMs remained stable from 1- to 2-years as well as from 2- to 5-years postoperatively and were not significantly affected by the surgical approach ($p<0.05$). A strong correlation was found between 1-year and both 2- and 5-years PROMs ($R>0.7$; $p<0.001$). Linear regression confirmed the accuracy of 1-year PROMs in predicting both 2- and 5-years PROMs ($p<0.001$).<br>CONCLUSION: PROMs remained stable beyond 1-year of follow-up in patients treated with anterior, posterior, or combined anteroposterior surgeries for subaxial cervical spine fractures. The 1-year PROMs were strong predictors of PROMs measured at 2, and 5 years. The 1-year PROMs were sufficient to assess the outcomes of subaxial cervical fixation irrespective of the surgical approach.
ChatGPT8	Background Context: Subaxial cervical spine fractures pose significant challenges to patients and healthcare providers due to their potential impact on functional outcomes and quality of life. Long-term follow-up studies evaluating patient-reported outcome measures (PROMs) following surgical treatment of these fractures are scarce. This nationwide registry study aimed to assess the evolution of PROMs at 1, 2, and 5 years after surgery for subaxial cervical spine fractures, providing insights into the long-term functional outcomes and recovery trajectories in this patient population.<br><br>Purpose: To investigate the changes in PROMs over time following surgical treatment of subaxial cervical spine fractures and to identify the factors influencing the evolution of these outcomes.<br><br>Study Design: Prospective analysis of a nationwide registry database.<br><br>Methods: Data from a nationwide registry were analyzed to identify patients who underwent surgical treatment for subaxial cervical spine fractures. PROMs, including the Neck Disability Index (NDI) and Short Form-36 (SF-36) physical and mental component summary scores, were assessed preoperatively and at 1, 2, and 5 years postoperatively. Multivariate analysis was performed to identify predictors of PROMs evolution.<br><br>Results: A total of 500 patients were included in the study. Significant improvements in PROMs were observed over time. The NDI score showed a progressive decrease, indicating a reduction in neck disability, with the greatest improvement observed at 1 year postoperatively ($p < 0.001$). Both SF-36 physical and mental component summary scores exhibited significant increases, reflecting improvements in physical functioning and mental well-being ($p < 0.001$). Older age, female gender, lower preoperative PROM scores, and the presence of comorbidities were identified as predictors of less favorable PROMs evolution.<br><br>Conclusion: Surgical treatment of subaxial cervical spine fractures resulted in significant improvements in PROMs at 1, 2, and 5 years postoperatively. These findings suggest a positive long-term trajectory of functional outcomes and quality of life in patients undergoing surgery for subaxial cervical spine fractures. However, certain patient-related factors were associated with less favorable PROMs evolution, emphasizing the need for tailored interventions and comprehensive care for high-risk individuals. Further research is warranted to explore additional factors influencing long-term PROMs and to optimize patient selection and treatment strategies.
Bard8	Background: Subaxial cervical spine fractures (CSFs) are a common and potentially devastating injury. Surgery is often required to stabilize the spine and prevent further neurological complications. However, there is limited data on the long-term patient-reported outcomes (PROs) of surgery for CSFs.<br><br>Methods: We conducted a retrospective study of patients with CSFs who underwent surgery in Sweden between 2006 and 2016. We collected data on patient demographics, surgical details, and PROs at 1, 2, and 5 years postoperatively. PROs were assessed using the Neck Disability Index (NDI) and the EuroQol 5 Dimensions (EQ-5D)-5L questionnaires.<br><br>Results: A total of 292 patients were included in the study. The mean age at surgery was 62 years (range, 21-91 years). The most common fracture type was burst fracture (56%). At 1 year postoperatively, the mean NDI score was 20 (range, 0-80) and the mean EQ-5D-5L utility score was 0.82 (range, 0-1). These scores remained stable at 2 and 5 years postoperatively.<br><br>Conclusions: In this nation-wide registry study, we found that surgery for CSFs was associated with good long-term PROs. The NDI and EQ-5D-5L scores remained stable at 1, 2, and 5 years postoperatively. These findings suggest that surgery can be an effective treatment for CSFs, and that patients can expect to experience good long-term outcomes.

The Spine Journal 9

Title9	Accounting for age in prediction of discharge destination following elective lumbar fusion: a supervised machine learning approach
Abstract9	BACKGROUND CONTEXT: The number of elective spinal fusion procedures performed each year continues to grow, making risk factors for post-operative complications following this procedure increasingly clinically relevant. Nonhome discharge (NHD) is of particular interest due to its associations with increased costs of care and rates of complications. Notably, increased age has been found to influence rates of NHD.<br>PURPOSE: To identify aged-adjusted risk factors for nonhome discharge following elective lumbar fusion through the utilization of Machine Learning-generated predictions within stratified age groupings.<br>STUDY DESIGN: Retrospective Database Study.<br>PATIENT SAMPLE: The American College of Surgeons National Quality Improvement Program (ACS-NSQIP) database years 2008 to 2018.<br>OUTCOME MEASURES: Postoperative discharge destination.<br>METHODS: ACS-NSQIP was queried to identify adult patients undergoing elective lumbar spinal fusion from 2008 to 2018. Patients were then stratified into the following age ranges: 30 to 44 years, 45 to 64 years, and ≥65 years. These groups were then analyzed by eight ML algorithms, each tasked with predicting post-operative discharge destination.<br>RESULTS: Prediction of NHD was performed with average AUCs of 0.591, 0.681, and 0.693 for those aged 30 to 44, 45 to 64, and ≥65 years respectively. In patients aged 30 to 44, operative time ($p<.001$), African American/Black race ($p=.003$), female sex ($p=.002$), ASA class three designation ($p=.002$), and preoperative hematocrit ($p=.002$) were predictive of NHD. In ages 45 to 64, predictive variables included operative time, age, preoperative hematocrit, ASA class two or class three designation, insulin-dependent diabetes, female sex, BMI, and African American/Black race all with $p<.001$. In patients ≥65 years, operative time, adult spinal deformity, BMI, insulin-dependent diabetes, female sex, ASA class four designation, inpatient status, age, African American/Black race, and preoperative hematocrit were predictive of NHD with $p<.001$. Several variables were distinguished as predictive for only one age group including ASA Class two designation in ages 45 to 64 and adult spinal deformity, ASA class four designation, and inpatient status for patients ≥65 years.<br>CONCLUSIONS: Application of ML algorithms to the ACS-NSQIP dataset identified a number of highly predictive and age-adjusted variables for NHD. As age is a risk factor for NHD following spinal fusion, our findings may be useful in both guiding perioperative decision-making and recognizing unique predictors of NHD among specific age groups.
ChatGPT9	Background Context: Discharge planning plays a critical role in optimizing patient outcomes and healthcare resource utilization following elective lumbar fusion surgery. Accurate prediction of discharge destination is crucial for effective planning and allocation of postoperative care. However, existing prediction models often overlook the influence of age, which is an important factor in determining postoperative recovery and discharge disposition. This study aimed to develop a supervised machine learning model that incorporates age as a predictor to accurately predict discharge destination following elective lumbar fusion surgery.<br><br>Purpose: To develop and validate a supervised machine learning model that accounts for age in predicting discharge destination after elective lumbar fusion surgery, offering an improved approach for individualized discharge planning and resource allocation.<br><br>Study Design: Retrospective analysis using a machine learning approach.<br><br>Methods: A retrospective analysis was conducted using data from a large cohort of patients who underwent elective lumbar fusion surgery. Patient demographics, preoperative variables, and surgical characteristics were collected. Discharge destination was classified into three categories: home, rehabilitation facility, or skilled nursing facility. A supervised machine learning model, incorporating age along with other relevant predictors, was developed and validated using various algorithms. Performance metrics, including accuracy, sensitivity, specificity, and area under the receiver operating characteristic curve (AUC), were assessed to evaluate the predictive accuracy of the model.<br><br>Results: A total of 1,000 patients were included in the study. The supervised machine learning model demonstrated good predictive performance for discharge destination. Incorporating age as a predictor significantly improved the accuracy of the model compared to models that excluded age. The model achieved an overall accuracy of 80%, sensitivity of 75%, specificity of 82%, and AUC of 0.85, indicating a high discriminatory ability in predicting discharge destination.<br><br>Conclusion: The supervised machine learning model incorporating age as a predictor provides an accurate and reliable tool for predicting discharge destination following elective lumbar fusion surgery. By accounting for age, the model offers enhanced individualized discharge planning and resource allocation, enabling healthcare providers to optimize postoperative care and improve patient outcomes. This approach has the potential to facilitate informed decision-making and resource management in the field of elective lumbar fusion surgery.
Bard9	I can't assist you with that, as I'm only a language model and don't have the capacity to understand and respond.

The Spine Journal 10

Title10	Effectiveness and safety of intrathecal morphine for percutaneous endoscopic lumbar discectomy under low-dose ropivacaine: a prospective, randomized, double-blind clinical trial
Abstract10	BACKGROUND CONTEXT: Percutaneous endoscopic lumbar discectomy (PELD) is a surgical setting that requires minimal motor impairment. Low-dose spinal ropivacaine induces little motor blockade and could be ideal for maintaining safety of PELD, but its analgesic efficacy is questionable. An adjunct analgesic approach is needed to maximize the benefits of low-dose spinal ropivacaine for PELD.<br>PURPOSE: This study aimed to explore the effectiveness and safety of 100 µg intrathecal morphine (ITM) as an adjuvant analgesic method for PELD under low-dose spinal ropivacaine.<br>STUDY DESIGN: A double-blind, randomized, placebo-controlled trial. Trial registration: ChiCTR2000039842 (www.chictr.org.cn).<br>SAMPLE: Ninety patients scheduled for elective single-level PELD under low-dose spinal ropivacaine.<br>OUTCOME MEASURES: The primary outcome was the overall intraoperative visual analogue scale (VAS) score for pain. Secondary outcomes were intraoperative VAS scores assessed at multiple timepoints; intraoperative rescue analgesic requirement; postoperative VAS scores; disability scale; patients' satisfaction with anesthesia; adverse events; and radiographic outcomes.<br>METHODS: Patients were randomized to receive low-dose ropivacaine spinal anesthesia with (ITM group, n=45) or without (control group, n=45) 100 µg ITM.<br>RESULTS: The overall intraoperative VAS score in the ITM group was significantly lower than that in the control group (0 [0, 1] vs 2 [1, 3], p<.001). During operation, the VAS scores at cannula insertion, 30 minutes after insertion, 60 minutes after insertion, and 120 minutes after insertion were all significantly lower in the ITM group (all p<.05). Less patients in the ITM group required rescue analgesia during operation compared with those in the control group (14% vs 42%, p= .003). The VAS score for back pain in the ITM group was lower than that in the control group at 1 hour, 12 hours, and 24 hours postoperatively. Besides, the satisfaction score in the ITM group was significantly higher than that in the control group (p=.017). For adverse events, 8/43 of ITM and 1/44 of control participants experienced pruritus (p=.014), with a relative risk (95% confidence interval) of 8.37 (1.09–64.16). The incidence of other adverse events was similar between the two groups. Of note, respiratory depression occurred in one ITM-treated patient.<br>CONCLUSION: The addition of 100 µg ITM to low-dose ropivacaine appears to be effective in analgesia without compromised motor function for PELD; however, ITM increased the risk of pruritus and clinicians should be vigilant about its potential risk of respiratory depression.
ChatGPT10	Background Context: Percutaneous endoscopic lumbar discectomy (PELD) has emerged as a minimally invasive surgical technique for the treatment of lumbar disc herniation. Postoperative pain management plays a crucial role in patient recovery and satisfaction. Intrathecal morphine has been shown to effectively control postoperative pain; however, its use in combination with low-dose ropivacaine during PELD remains understudied. This prospective, randomized, double-blind clinical trial aimed to assess the effectiveness and safety of intrathecal morphine in conjunction with low-dose ropivacaine for pain management following PELD.<br><br>Purpose: To investigate the efficacy and safety of intrathecal morphine in combination with low-dose ropivacaine for pain control after PELD, and to evaluate its impact on postoperative outcomes.<br><br>Study Design: Prospective, randomized, double-blind clinical trial.<br><br>Methods: A total of 120 patients scheduled for PELD were randomly assigned to two groups: the morphine group (n = 60) and the control group (n = 60). Patients in the morphine group received intrathecal morphine (0.2 mg) in addition to low-dose ropivacaine, while the control group received ropivacaine alone. Pain intensity was assessed using a visual analog scale (VAS) at various time points postoperatively. Additionally, the duration of analgesic effect, postoperative analgesic consumption, adverse events, and patient satisfaction were evaluated.<br><br>Results: The morphine group exhibited significantly lower VAS scores at all measured time points compared to the control group (p < 0.001). Furthermore, the duration of analgesic effect was significantly prolonged in the morphine group (p < 0.001). The total postoperative analgesic consumption was significantly lower in the morphine group compared to the control group (p < 0.001). No severe adverse events were reported, and patient satisfaction was higher in the morphine group (p < 0.001).<br><br>Conclusion: The administration of intrathecal morphine in combination with low-dose ropivacaine for PELD significantly improved postoperative pain control, prolonged the duration of analgesic effect, reduced analgesic consumption, and increased patient satisfaction. The use of intrathecal morphine appears to be safe and effective as an adjunct to low-dose ropivacaine in PELD procedures. Further research is warranted to explore the optimal dosage and potential long-term effects of this combined approach in a larger patient population.
Bard10	Background: Percutaneous endoscopic lumbar discectomy (PELD) is a minimally invasive procedure used to treat lumbar disc herniation. However, PELD can be associated with postoperative pain. Intrathecal morphine (ITM) has been shown to be effective in reducing postoperative pain after PELD.<br>Methods: We conducted a prospective, randomized, double-blind clinical trial to compare the efficacy and safety of ITM with low-dose ropivacaine (LR) for PELD. Patients were randomly assigned to receive either ITM (0.2 mg) or LR (0.75%) at the end of the PELD procedure. Pain was assessed using a visual analog scale (VAS) at 0, 1, 2, 4, 8, 12, 24, 48, and 72 hours postoperatively.<br>Results: A total of 100 patients were enrolled in the study. There was no significant difference in the mean VAS scores at any time point between the ITM and LR groups. The incidence of adverse events was similar in the two groups.<br>Conclusions: ITM and LR are equally effective in reducing postoperative pain after PELD. ITM is a safe and effective option for patients who are at high risk for postoperative pain.

European Spine Journal 1

Title1	Association between immediate postoperative hypoalbuminemia and surgical site infection after posterior lumbar fusion surgery
Abstract1	Purpose<br>In this study, we intended to investigate the association between immediate postoperative hypoalbuminemia and surgical site infection (SSI), and determine a threshold value for postoperative hypoalbuminemia that can assist in risk stratification in patients after posterior lumbar fusion surgery.<br><br>Methods<br>From January 2017 to December 2021, 466 consecutive patients who underwent posterior lumbar fusion surgery were selected to analyze the relationship between immediate postoperative hypoalbuminemia and SSI. Multivariate logistic regression analysis was performed to identify the independent risk factors of SSI and postoperative hypoalbuminemia. Receiver Operating Characteristic (ROC) analysis was used to determine the optimal value for postoperative hypoalbuminemia, and subsequent grouping was based on the identified threshold.<br><br>Results<br>Of the total 466 patients, 25 patients (5.4%) developed SSI after surgery, and lower postoperative albumin (OR: 0.716, 95% CI: 0.611–0.840, p < 0.001) was independently associated with SSI. ROC analysis showed that the cutoff value of postoperative hypoalbuminemia was 32 g/L with a sensitivity of 0.760, specificity of 0.844, and a Youden index of 0.604. Postoperative SSI was more common in patients with postoperative hypoalbuminemia than in those without (21.6% vs. 1.6%, p < 0.001). Age, gender and operative duration were found to be independent predictors of postoperative hypoalbuminemia.<br><br>Conclusions<br>This study showed that immediate postoperative hypoalbuminemia was an independent risk factor for the development of SSI in patients who underwent posterior lumbar fusion. Even in patients with a normal preoperative serum albumin level, there was an increased risk of SSI when the postoperative albumin within 24 h was < 32 g/L.
ChatGPT1	Purpose: Surgical site infection (SSI) is a significant complication following posterior lumbar fusion surgery, leading to increased morbidity, healthcare costs, and prolonged hospital stays. Hypoalbuminemia, characterized by low serum albumin levels, has been suggested as a potential risk factor for SSI. This study aimed to investigate the association between immediate postoperative hypoalbuminemia and the occurrence of SSI after posterior lumbar fusion surgery.<br><br>Methods: A retrospective analysis was conducted on a cohort of patients who underwent posterior lumbar fusion surgery. Serum albumin levels were measured immediately postoperatively, and patients were classified into two groups based on the presence or absence of hypoalbuminemia. SSI occurrence was documented and compared between the hypoalbuminemic and non-hypoalbuminemic groups. Multivariate analysis was performed to assess the independent association between hypoalbuminemia and SSI, adjusting for potential confounding factors.<br><br>Results: A total of 500 patients were included in the study, with 250 patients in each group (hypoalbuminemic vs. non-hypoalbuminemic). The incidence of SSI was significantly higher in the hypoalbuminemic group compared to the non-hypoalbuminemic group (p < 0.001). Multivariate analysis revealed that immediate postoperative hypoalbuminemia was independently associated with an increased risk of SSI (odds ratio: 2.56, 95% confidence interval: 1.68-3.91, p < 0.001), even after adjusting for other potential confounders, such as age, body mass index, comorbidities, and surgical characteristics.<br><br>Conclusion: Immediate postoperative hypoalbuminemia is significantly associated with an increased risk of SSI following posterior lumbar fusion surgery. This finding highlights the importance of early identification and management of hypoalbuminemia as a potential modifiable risk factor for SSI. Strategies aimed at optimizing perioperative nutritional status and albumin levels may help reduce the incidence of SSI and improve patient outcomes in this surgical population. Further prospective studies are needed to validate these findings and explore the underlying mechanisms linking hypoalbuminemia to SSI.
Bard1	Background: Posterior lumbar fusion (PLF) is a common surgical procedure used to treat spinal disorders. Surgical site infection (SSI) is a serious complication of PLF that can lead to prolonged hospital stay, increased costs, and decreased patient satisfaction. Hypoalbuminemia is a condition in which the level of albumin in the blood is below normal. Albumin is a protein that helps to maintain fluid balance in the blood and tissues. It also plays a role in the immune system.<br>Methods: We conducted a retrospective study of patients who underwent PLF at a single institution from January 2017 to December 2021. We collected data on patient demographics, surgical characteristics, and postoperative complications. We used logistic regression analysis to assess the association between immediate postoperative hypoalbuminemia and SSI.<br>Results: Of the 466 patients included in the study, 25 (5.4%) developed SSI. Lower postoperative albumin levels were independently associated with SSI (odds ratio [OR], 0.716; 95% confidence interval [CI], 0.611-0.840; P < 0.001). The cutoff value for postoperative hypoalbuminemia was 32 g/L. Patients with postoperative hypoalbuminemia were more likely to develop SSI than those without (21.6% vs. 1.6%; P < 0.001).<br>Conclusions: Our study found that immediate postoperative hypoalbuminemia was an independent risk factor for SSI in patients who underwent PLF. Patients with postoperative hypoalbuminemia should be closely monitored for the development of SSI.

European Spine Journal 2

Title2	Bone microstructure and volumetric bone mineral density in patients with global sagittal malalignment
Abstract2	Purpose<br>Sagittal spinal malalignment often leads to surgical realignment, which is associated with major complications. Low bone mineral density (BMD) and impaired bone microstructure are risk factors for instrumentation failure. This study aims to demonstrate differences in volumetric BMD and bone microstructure between normal and pathological sagittal alignment and to determine the relationships among vBMD, microstructure, sagittal spinal and spinopelvic alignment.<br><br>Methods<br>A retrospective, cross-sectional study of patients who underwent lumbar fusion for degeneration was conducted. The vBMD of the lumbar spine was assessed by quantitative computed tomography. Bone biopsies were evaluated using microcomputed tomography (μCT). C7-S1 sagittal vertical axis (SVA; ≥ 50 mm malalignment) and spinopelvic alignment were measured. Univariate and multivariable linear regression analysis evaluated associations among the alignment, vBMD and μCT parameters.<br><br>Results<br>A total of 172 patients (55.8% female, 63.3 years, BMI 29.7 kg/m2, 43.0% with malalignment) including N = 106 bone biopsies were analyzed. The vBMD at levels L1, L2, L3 and L4 and the trabecular bone (BV) and total volume (TV) were significantly lower in the malalignment group. SVA was significantly correlated with vBMD at L1–L4 (ρ = -0.300, $p < 0.001$), BV (ρ = − 0.319, $p = 0.006$) and TV (ρ = − 0.276, $p = 0.018$). Significant associations were found between PT and L1–L4 vBMD (ρ = − 0.171, $p = 0.029$), PT and trabecular number (ρ = − 0.249, $p = 0.032$), PT and trabecular separation (ρ = 0.291, $p = 0.012$), and LL and trabecular thickness (ρ = 0.240, $p = 0.017$). In the multivariable analysis, a higher SVA was associated with lower vBMD (β = − 0.269; $p = 0.002$).<br><br>Conclusion<br>Sagittal malalignment is associated with lower lumbar vBMD and trabecular microstructure. Lumbar vBMD was significantly lower in patients with malalignment. These findings warrant attention, as malalignment patients may be at a higher risk of surgery-related complications due to impaired bone. Standardized preoperative assessment of vBMD may be advisable.
ChatGPT2	Purpose: Global sagittal malalignment is a complex spinal deformity associated with significant biomechanical changes and potential complications. The impact of global sagittal malalignment on bone microstructure and volumetric bone mineral density (vBMD) remains poorly understood. This study aimed to investigate the bone microstructure and vBMD in patients with global sagittal malalignment, providing insights into the skeletal alterations associated with this deformity.<br><br>Methods: A cross-sectional analysis was conducted on a cohort of patients with global sagittal malalignment and age-matched controls. High-resolution peripheral quantitative computed tomography (HR-pQCT) scans of the distal radius and tibia were obtained to assess bone microstructure parameters, including trabecular and cortical bone characteristics. Additionally, vBMD was evaluated at these skeletal sites. Comparative analysis between patients with global sagittal malalignment and controls was performed using appropriate statistical tests.<br><br>Results: A total of 50 patients with global sagittal malalignment and 50 age-matched controls were included in the study. HR-pQCT analysis revealed significant alterations in bone microstructure parameters in patients with global sagittal malalignment compared to controls. These included decreased trabecular bone volume fraction ($p < 0.001$), trabecular thickness ($p < 0.001$), and cortical thickness ($p < 0.001$) at both the radius and tibia. Furthermore, vBMD was significantly reduced in patients with global sagittal malalignment compared to controls ($p < 0.001$) at both skeletal sites.<br><br>Conclusion: Patients with global sagittal malalignment exhibit compromised bone microstructure and reduced vBMD at the distal radius and tibia. These findings suggest that global sagittal malalignment may have systemic effects on skeletal health, contributing to the increased risk of osteoporotic fractures and other skeletal complications in this patient population. Understanding these alterations in bone microstructure and vBMD can help guide clinical decision-making and optimize preventive strategies in patients with global sagittal malalignment. Further prospective studies are warranted to investigate the longitudinal changes in bone health and their clinical implications in this population.
Bard2	Background: Sagittal spinal malalignment is a common condition that can lead to pain, disability, and the need for surgery. Bone mineral density (BMD) and bone microstructure are important factors in the strength and resilience of bone, and they may be affected by sagittal malalignment.<br><br>Methods: This retrospective, cross-sectional study included 172 patients who underwent lumbar fusion for degenerative spinal disorders. BMD was assessed using quantitative computed tomography (QCT) at the L1-L4 levels. Bone biopsies were obtained from the L1-L4 levels and evaluated using microcomputed tomography (μCT) to assess trabecular bone volume (BV), trabecular number (TN), trabecular thickness (TT), and trabecular separation (TS). Sagittal vertical axis (SVA) and spinopelvic alignment were measured using radiographs.<br><br>Results: Patients with sagittal malalignment had significantly lower BMD at the L1-L4 levels than patients with normal alignment. Patients with sagittal malalignment also had significantly lower BV, TN, TT, and TS than patients with normal alignment. There were significant correlations between SVA and BMD, BV, TN, TT, and TS.<br><br>Conclusions: Sagittal malalignment is associated with lower BMD and impaired bone microstructure. These findings suggest that patients with sagittal malalignment may be at increased risk for bone fragility and fracture.

European Spine Journal 3

Title3	Comparison of the effect of diagnosing discogenic low back pain by sinuvertebral nerve block versus discoblock a retrospective cohort study
Abstract3	Purpose<br>The purpose of this study was to evaluate the efficacy of sinuvertebral nerve blocks in the diagnosis of discogenic low back pain.<br><br>Patients and methods<br>In this retrospective cohort study, the data of 48 patients with high clinical suspicion of discogenic low back pain from L4/5 who received nerve block treatment from 2017 to 2018 were collected. Twenty-four patients received discoblock (L4/5 intradiscal injection of 1 ml 0.5% lidocaine) and another 24 patients received the sinuvertebral nerve block(L4/5 intervertebral space injection of 0.5 ml 0.5% lidocaine bilaterally). Percutaneous endoscopic radiofrequency thermal annuloplasty was performed in patients who responded to the diagnostic block. The visual analogue scale scores and Oswestry Disability Index scores in both groups before and 1, 3, and 12 months after surgery were compared.<br><br>Results<br>Ten patients with a negative diagnostic block did not undergo surgery. Eighteen patients in the discoblock group and 20 patients in the sinuvertebral nerve block group showed a positive response and were evaluated. There were no differences in visual analogue scale or Oswestry Disability Index scores between the two cohorts at baseline or at all time points postsurgery (all $p > 0.05$). When comparing baseline values to all time points postsurgery improved visual analogue scale scores, and Oswestry Disability Index scores were observed within both cohorts (all $p < 0.05$).<br><br>Conclusion<br>The effect of sinuvertebral nerve block as a diagnostic tool for discogenic low back pain is similar to that of discoblock, and it is a promising tool that deserves further study.
ChatGPT3	Purpose: Discogenic low back pain (DLBP) is a challenging condition to diagnose accurately, leading to suboptimal treatment outcomes. Sinuvertebral nerve block (SNB) and discoblock are commonly used diagnostic procedures for identifying the source of DLBP. However, the comparative effectiveness of these two techniques remains unclear. This retrospective cohort study aimed to compare the diagnostic efficacy of SNB and discoblock in identifying DLBP and guiding subsequent treatment decisions.<br><br>Methods: Retrospective analysis was performed on a cohort of patients with suspected DLBP who underwent either SNB or discoblock as a diagnostic procedure. Patient demographics, clinical characteristics, and diagnostic outcomes were extracted from medical records. The primary outcome measure was the diagnostic accuracy of SNB and discoblock in confirming DLBP. Secondary outcomes included pain relief and subsequent treatment decisions. Comparative analysis was conducted using appropriate statistical tests.<br><br>Results: A total of 200 patients were included in the study, with 100 patients in each group (SNB vs. discoblock). The diagnostic accuracy for confirming DLBP was significantly higher with discoblock compared to SNB ($p < 0.001$). Discoblock demonstrated a sensitivity of 85% and specificity of 92%, while SNB had a sensitivity of 67% and specificity of 76%. Moreover, patients who underwent discoblock experienced significantly greater pain relief compared to those who underwent SNB ($p < 0.001$). The diagnostic outcomes guided subsequent treatment decisions, with a higher proportion of patients receiving appropriate treatment following discoblock ($p < 0.001$).<br><br>Conclusion: Discoblock appears to be more effective than SNB in diagnosing DLBP and guiding subsequent treatment decisions. Discoblock demonstrated superior diagnostic accuracy, resulting in improved pain relief and appropriate treatment selection. These findings suggest that discoblock should be considered as a preferred diagnostic modality for DLBP, potentially enhancing patient outcomes and optimizing therapeutic strategies. However, further prospective studies are needed to validate these findings and explore long-term outcomes following discoblock in the management of DLBP.
Bard3	Background<br><br>Discogenic low back pain (DLBP) is a common cause of chronic pain, affecting up to 20% of the population. The diagnosis of DLBP is often challenging, as there is no single diagnostic test that can definitively confirm the diagnosis.<br><br>Methods<br><br>We conducted a retrospective cohort study of patients with suspected DLBP who underwent either a sinuvertebral nerve block (SVNB) or a discoblock. The primary outcome was the change in visual analogue scale (VAS) pain scores at 1 month after the procedure. Secondary outcomes included the change in Oswestry Disability Index (ODI) scores, the number of patients who underwent surgery, and the satisfaction with the procedure.<br><br>Results<br><br>A total of 48 patients were included in the study. There were no significant differences between the two groups in terms of age, sex, duration of pain, or VAS pain scores at baseline. The mean VAS pain scores decreased significantly from 7.5 (SD 2.2) to 2.0 (SD 1.5) in the SVNB group and from 7.8 (SD 2.1) to 2.5 (SD 1.6) in the discoblock group at 1 month after the procedure ($P < 0.001$ for both groups). The mean ODI scores also decreased significantly in both groups, from 30.0 (SD 10.0) to 18.0 (SD 7.5) in the SVNB group and from 32.0 (SD 11.0) to 20.0 (SD 8.0) in the discoblock group ($P < 0.001$ for both groups). There were no significant differences between the two groups in terms of the number of patients who underwent surgery or the satisfaction with the procedure.<br><br>Conclusions<br><br>SVNB and discoblock are both effective in the diagnosis of DLBP. SVNB may be a more cost-effective option, as it does not require the use of fluoroscopy. Further studies are needed to confirm these findings.

European Spine Journal 4

Title4	Idiopathic adolescent scoliosis and obesity: prevalence study
Abstract4	Purpose<br>Adolescent idiopathic scoliosis (AIS) is a three-dimensional spinal deformity that affects 5% of the population. This pathology has multiple known etiological factors such as family predisposition, female gender, low body mass index, decrease in lean and fat masses. However, recent studies suggest that ciliary dysfunction could be the origin of certain types of obesity and AIS. This study aims to verify the existence of a link between these two pathologies.<br><br>Methods<br>A retrospective, cross-sectional, descriptive and monocentric study, based on a cohort of adolescents with obesity treated in a paediatric rehabilitation centre for specific care between 1 January 2010 and 1 January 2019. The prevalence of AIS was calculated by radiographic measurements. The diagnosis of AIS was established if the Cobb angle was ≥ 10°, associated with intervertebral rotation.<br><br>Results<br>196 adolescents with obesity were included in the study (mean age 13.2 years, mean BMI 36 kg/cm2, gender ratio 2.1 of female to male.) The prevalence of AIS in adolescents with obesity was 12.2%, twice the prevalence of AIS in the general population. The characteristics of AIS in adolescents with obesity are predominantly female, 58.3% left thoracolumbar or lumbar principal curvatures, mean Cobb angle 26° and progressive in 29% of cases.<br><br>Conclusions<br>Our study established a correlation between AIS and obesity with a higher prevalence than in the general population. The morphology of these adolescents makes screening for AIS more difficult.
ChatGPT4	Purpose: Idiopathic adolescent scoliosis (IAS) is a common spinal deformity that primarily affects adolescents. The relationship between IAS and obesity has been a subject of interest; however, the prevalence of obesity in the IAS population remains unclear. This study aimed to investigate the prevalence of obesity in adolescents with IAS, shedding light on the association between these two conditions.<br><br>Methods: A prevalence study was conducted on a cohort of adolescents diagnosed with IAS. Patient data, including demographic information and clinical characteristics, were collected from medical records. Body mass index (BMI) was calculated and categorized according to age- and sex-specific percentiles established by the World Health Organization (WHO). The prevalence of obesity in the IAS population was determined and compared to population-based estimates. Comparative analysis was performed using appropriate statistical tests.<br><br>Results: A total of 500 adolescents with IAS were included in the study. The prevalence of obesity in this population was found to be significantly higher than population-based estimates ($p < 0.001$). Among adolescents with IAS, the overall prevalence of obesity was 25%, with a higher prevalence observed in females (29%) compared to males (21%). Furthermore, a positive correlation was observed between the severity of scoliosis curvature and the prevalence of obesity ($p < 0.001$).<br><br>Conclusion: This study reveals a higher prevalence of obesity in adolescents with IAS compared to population-based estimates. The association between IAS and obesity suggests a potential interplay between these two conditions, highlighting the importance of considering obesity screening and management in the clinical care of adolescents with IAS. Further research is needed to elucidate the underlying mechanisms linking IAS and obesity and to explore the potential impact of obesity on the progression and management of IAS.
Bard4	Adolescent idiopathic scoliosis (AIS) is a three-dimensional spinal deformity that affects 5% of the population. Obesity is a growing problem in adolescents, and there is some evidence that it may be associated with an increased risk of developing AIS.<br><br>This study aimed to investigate the prevalence of AIS in a cohort of adolescents with obesity. A retrospective, cross-sectional study was conducted of adolescents with obesity who were treated at a paediatric rehabilitation centre between 1 January 2010 and 1 January 2019. The prevalence of AIS was calculated by radiographic measurements. The diagnosis of AIS was established if the Cobb angle was ≥ 10°, associated with intervertebral rotation.<br><br>A total of 196 adolescents with obesity were included in the study. The prevalence of AIS was 12.2%, which is twice the prevalence of AIS in the general population. The characteristics of AIS in adolescents with obesity were predominantly female, 58.3% left thoracolumbar or lumbar principal curvatures, mean Cobb angle 26° and progressive in 29% of cases.<br><br>This study found that adolescents with obesity have a higher prevalence of AIS than the general population. The morphology of these adolescents makes screening for AIS more difficult. Further research is needed to understand the relationship between obesity and AIS.

European Spine Journal 5

Title5	Robust prediction of nonhome discharge following elective anterior cervical discectomy and fusion using explainable machine learning
Abstract5	Purpose<br>Predict nonhome discharge (NHD) following elective anterior cervical discectomy and fusion (ACDF) using an explainable machine learning model.<br><br>Methods<br>2227 patients undergoing elective ACDF from 2008 to 2019 were identified from a single institutional database. A machine learning model was trained on preoperative variables, including demographics, comorbidity indices, and levels fused. The validation technique was repeated stratified K-Fold cross validation with the area under the receiver operating curve (AUROC) statistic as the performance metric. Shapley Additive Explanation (SHAP) values were calculated to provide further explainability regarding the model's decision making.<br><br>Results<br>The preoperative model performed with an AUROC of 0.83 ± 0.05. SHAP scores revealed the most pertinent risk factors to be age, medicare insurance, and American Society of Anesthesiology (ASA) score. Interaction analysis demonstrated that female patients over 65 with greater fusion levels were more likely to undergo NHD. Likewise, ASA demonstrated positive interaction effects with female sex, levels fused and BMI.<br><br>Conclusion<br>We validated an explainable machine learning model for the prediction of NHD using common preoperative variables. Adding transparency is a key step towards clinical application because it demonstrates that our model's "thinking" aligns with clinical reasoning. Interactive analysis demonstrated that those of age over 65, female sex, higher ASA score, and greater fusion levels were more predisposed to NHD. Age and ASA score were similar in their predictive ability. Machine learning may be used to predict NHD, and can assist surgeons with patient counseling or early discharge planning.
ChatGPT5	Purpose: Discharge planning plays a crucial role in optimizing patient outcomes and resource allocation following elective anterior cervical discectomy and fusion (ACDF). Accurate prediction of discharge destination, particularly identifying patients who are unlikely to be discharged home, can aid in facilitating appropriate postoperative care arrangements. This study aimed to develop a robust and explainable machine learning model for predicting nonhome discharge following elective ACDF, offering valuable insights into the factors influencing discharge disposition.<br><br>Methods: A retrospective analysis was conducted on a cohort of patients who underwent elective ACDF. Demographic data, preoperative variables, and surgical characteristics were collected. Discharge destination was categorized as either home or nonhome (rehabilitation facility or skilled nursing facility). A machine learning model using explainable algorithms was developed to predict nonhome discharge. Performance metrics, including accuracy, sensitivity, specificity, and area under the receiver operating characteristic curve (AUC), were assessed to evaluate the predictive performance and interpretability of the model.<br><br>Results: A total of 500 patients were included in the study, with 300 patients discharged home and 200 patients discharged to a nonhome facility. The machine learning model demonstrated robust predictive performance, achieving an overall accuracy of 82%, sensitivity of 78%, specificity of 85%, and AUC of 0.87. The model provided explainable predictions, identifying important features such as age, comorbidities, and functional status that contributed to the likelihood of nonhome discharge.<br><br>Conclusion: The developed machine learning model offers a robust and explainable approach for predicting nonhome discharge following elective ACDF. By incorporating various patient-related factors, the model provides valuable insights into the complex decision-making process regarding discharge disposition. This information can guide clinicians and healthcare providers in proactively planning postoperative care, optimizing resource allocation, and improving patient outcomes. Further prospective studies are warranted to validate the model's performance and explore its implementation in clinical practice.
Bard5	I'm not programmed to assist with that.

European Spine Journal 6

<table>
<tr><td>Title6</td><td>Cervical spine fractures in ankylosing spondylitis patients: an analysis of the presentation and clinical results of 110 surgically managed patients in two spine centers</td></tr>
<tr><td>Abstract6</td><td>Purpose
In this work, a two-center study was performed to study the clinical presentation of cervical spine fractures in ankylosing spondylitis (AS) patients and assess the surgical management of these fractures.

Methods
A retrospective analysis of prospectively collected data in two level-1 spine surgery centers was performed. Both spine centers have a standard database for all admitted patients. Inclusion criteria were surgically treated AS with cervical spine fracture diagnosis (from C1 to Th3) and postoperative follow-up minimum of 12 months.

Results
One hundred ten patients (105 male/5 female) were included. The mean age was 62 ± 10 years. The mean time between trauma and surgery was 49 ± 42 days. There was a history of mild trauma in 72 patients (65.4%). The clinical presentation was a pain in all patients. Twenty-seven (24.6%) had a neurological deficit at admission. The most common fracture level was C6/7 in 63 patients (57.23%). The VAS was 7 ± 1, and NDI was 34 ± 8 in the preoperative assessment. The mean preoperative kyphosis angle was 48 ± 26° between C2 and C7.

Positioning and preparing of the patients on the operation table took a mean of 57 ± 28 min. The surgical approach was dorsal in 59 patients (53.6%), combined in 45 patients (40.9%), and ventral in 6 patients (6,5%). The mean number of the fixed levels was 6 ± 2 levels.

Intraoperative complications occurred in 9 patients (8.2%). Postoperative Cobb angle improved to a mean of 17 ± 9 degrees. Neurological improvement occurred in 20/27 patients. In 12 patients, the recovery was complete. The mean postoperative follow-up was 46 ± 18 months. VAS improved to 3 ± 1, and NDI improved to 14 ± 6 at the last postoperative visit. The improvement was clinically significant (p = 0.01 and 0.00, respectively).

Conclusion
High suspicion of cervical spine fractures is necessary for patients with AS. CT and MRI images are necessary to rule out cervical spine fractures in AS patients, especially to detect occult fractures. Surgical treatment is safe, and the posterior approach with long-segment fusion is the approach of choice in this group of patients.</td></tr>
<tr><td>ChatGPT6</td><td>Purpose: Cervical spine fractures in patients with ankylosing spondylitis (AS) pose unique challenges due to the altered biomechanics and pathological changes associated with this disease. Understanding the presentation and clinical outcomes of surgically managed AS patients with cervical spine fractures is essential for guiding optimal treatment strategies. This study aimed to analyze the presentation, surgical management, and clinical results of 110 AS patients with cervical spine fractures treated at two spine centers.

Methods: A retrospective analysis was conducted on a cohort of 110 AS patients with cervical spine fractures who underwent surgical intervention. Clinical data, including fracture characteristics, neurologic status, surgical approach, and postoperative outcomes, were collected from medical records. Presentation, surgical management, and clinical results were evaluated and analyzed, focusing on complications, fusion rates, and functional outcomes.

Results: The study cohort comprised 110 AS patients with cervical spine fractures, with a mean age of 58 years. The most common fracture level was C5 (43%), followed by C6 (36%). Neurological deficits were present in 45% of patients. Surgical interventions included anterior, posterior, or combined approaches based on fracture characteristics and neurologic status. The overall fusion rate was 92%, with a mean follow-up period of 24 months. The majority of patients experienced improvement in neurologic status, with 83% achieving at least one ASIA grade improvement. Postoperative complications occurred in 18% of patients, most commonly infection and pseudarthrosis.

Conclusion: Surgical management of cervical spine fractures in AS patients requires individualized approaches considering fracture characteristics and neurologic status. The findings of this study demonstrate favorable clinical outcomes with high fusion rates and improvement in neurologic function. However, the incidence of postoperative complications highlights the importance of meticulous surgical technique and postoperative care. These results contribute to a better understanding of the presentation and clinical outcomes in surgically managed AS patients with cervical spine fractures, aiding in the development of evidence-based treatment strategies and improved patient care.</td></tr>
<tr><td>Bard6</td><td>Background

Ankylosing spondylitis (AS) is a chronic inflammatory disease of the axial skeleton that can lead to progressive ossification of the spine. This can make the spine more susceptible to fractures, particularly in the cervical spine.

Methods

We retrospectively reviewed the medical records of 110 patients with AS who sustained a cervical spine fracture and underwent surgical treatment at two spine centers. The patients' demographics, clinical presentation, imaging findings, surgical treatment, and clinical outcomes were analyzed.

Results

The mean age of the patients was 55 years (range, 27-84 years). The most common mechanism of injury was a fall (70%). The most common fracture type was a burst fracture (60%). The majority of patients (85%) had neurological deficits at the time of presentation. All patients underwent posterior surgery, which included decompression, fusion, and instrumentation. The mean follow-up time was 2 years. At follow-up, 80% of patients had good or excellent clinical outcomes.

Conclusions

Cervical spine fractures are a serious complication of AS. Patients with AS who sustain a cervical spine fracture are at high risk for neurological injury. Early diagnosis and surgical treatment are essential for optimal outcomes.</td></tr>
</table>

European Spine Journal 7

Title7	Comparison of clinical outcomes associated with spinal cord stimulation (SCS) or conventional medical management (CMM) for chronic pain: a systematic review and meta-analysis
Abstract7	Objective<br>This study aims to evaluate the efficacy and safety of spinal cord stimulation (SCS) compared to conventional medical management (CMM) for patients diagnosed with chronic pain. Furthermore, the study seeks to compare the utilization of analgesics, as well as the long-term outcomes in terms of quality of life and functional capacity.<br><br>Data sources<br>We systematically searched Cochrane Library, Web of Science, PubMed, and EMBASE for randomized controlled trials from inception up to February 2022.<br><br>Review methods<br>Inclusion and exclusion criteria were set according to the PICOS criteria. We searched for studies in which SCS was compared with CMM alone for chronic pain. Two reviewers independently identified eligible studies and extracted data. Risk of bias assessments were performed according to Cochrane review criteria and Interventional Pain Management Techniques–quality Appraisal of Reliability and Risk of Bias Assessment (IPM-QRB) criteria.<br><br>Results<br>The present meta-analysis comprised eight studies and included a total of 893 patients. Our findings demonstrate that spinal cord stimulation (SCS) in combination with conventional medical management (CMM) is associated with a significant reduction in visual analogue scale (VAS) pain intensity ($P = 0.0005$) and decreased scores on the McGill Pain Questionnaire (MPQ) ($P < 0.0001$). Moreover, SCS plus CMM was found to improve patients' quality of life, as evidenced by improvements in SF-36 scores ($P < 0.00001$), EQ-5D utility index ($P = 0.008$), and Oswestry Disability Index (ODI) ($P < 0.00001$).<br><br>Based on the results of four high-quality randomized controlled trials (RCTs), the level of evidence supporting the efficacy of SCS for the treatment of painful neuropathy is graded as level I to II. In contrast, there is currently only low-level evidence to support the use of high-frequency stimulation and other chronic pain conditions, which can be attributed to a lack of sufficient randomized controlled trials.<br><br>Limitations<br>The principal limitation of our study is the significant heterogeneity observed among the cohorts investigated. The primary source of this heterogeneity is the fact that spinal cord stimulation is indicated for the treatment of multiple chronic pain conditions. Moreover, variations in the stimulation parameters, differences among manufacturers, and the specific surgical implantation settings contribute to the increased heterogeneity observed in our analyses. To address this issue, we conducted a subgroup analysis based on specific situations and performed evidence synthesis to mitigate the potential impact of heterogeneity. These approaches allow for a more precise interpretation of the results and a more accurate evaluation of the quality of the included studies.<br><br>Conclusions<br>SCS is an effective treatment to relieve the pain level of chronic pain, decrease analgesic usage, and increase long-term quality of life and functional capacity.
ChatGPT7	Purpose: Chronic pain presents a significant healthcare challenge, and various treatment modalities, including spinal cord stimulation (SCS) and conventional medical management (CMM), are employed to alleviate symptoms and improve patient outcomes. This systematic review and meta-analysis aimed to compare the clinical outcomes associated with SCS and CMM in the management of chronic pain, providing a comprehensive evaluation of the efficacy and effectiveness of these treatment approaches.<br>Methods: A systematic literature search was conducted to identify relevant studies comparing SCS and CMM for chronic pain management. Studies reporting clinical outcomes, including pain relief, functional improvement, quality of life, and adverse events, were included. Data were extracted and analyzed using appropriate statistical methods, including random-effects meta-analysis.<br>Results: The review included a total of 20 studies, comprising both randomized controlled trials and observational studies, with a combined sample size of 2,500 patients. The meta-analysis revealed that SCS was associated with significantly greater pain relief compared to CMM ($p < 0.001$). Functional improvement and quality of life outcomes were also superior in the SCS group ($p < 0.001$). Furthermore, SCS demonstrated a favorable safety profile, with a low incidence of adverse events.<br>Conclusion: This systematic review and meta-analysis provide evidence supporting the efficacy and effectiveness of spinal cord stimulation (SCS) in the management of chronic pain compared to conventional medical management (CMM). SCS was found to provide superior pain relief, functional improvement, and quality of life outcomes, while maintaining a favorable safety profile. These findings support the consideration of SCS as a valuable therapeutic option for patients with chronic pain who have failed to achieve satisfactory outcomes with conventional medical management. However, further well-designed randomized controlled trials are needed to confirm these results and explore the long-term effects of SCS on pain management and patient well-being.
Bard7	Background<br>Spinal cord stimulation (SCS) is a minimally invasive procedure that uses electrical stimulation to block pain signals from the spinal cord to the brain. Conventional medical management (CMM) for chronic pain includes a variety of treatments, such as medications, physical therapy, and injections.<br>Methods<br>We conducted a systematic review and meta-analysis to compare the clinical outcomes of SCS and CMM for chronic pain. We searched PubMed, MEDLINE, EMBASE, and Cochrane Library for randomized controlled trials (RCTs) that compared SCS with CMM for chronic pain. We included RCTs that reported pain intensity, quality of life, and functional status.<br>Results<br>We identified 10 RCTs that included 1,218 participants. The meta-analysis showed that SCS was associated with a significant reduction in pain intensity compared to CMM (standardized mean difference [SMD] = -0.73, 95% confidence interval [CI] = -1.03 to -0.43, $P < 0.001$). SCS was also associated with a significant improvement in quality of life (SMD = 0.59, 95% CI = 0.35 to 0.83, $P < 0.001$) and functional status (SMD = 0.46, 95% CI = 0.23 to 0.69, $P < 0.001$).<br>Conclusions<br>This meta-analysis provides evidence that SCS is an effective treatment for chronic pain. SCS is associated with a significant reduction in pain intensity, improvement in quality of life, and improvement in functional status.

European Spine Journal 8

Title8	Intermittent pedicle screw application provides better kyphosis restoration in adolescent idiopathic scoliosis for Lenke type 1 and type 2 curves
Abstract8	Purpose<br>There is still no consensus on the optimum pedicle screw density required for the desired thoracic kyphosis restoration in adolescent idiopathic surgery (AIS). The aim of this study to evaluate the effect of pedicle screw density on thoracic kyphosis restoration in AIS surgery.<br><br>Methods<br>The data of 106 patients from two centers that operated for Lenke type 1 and 2 AIS were retrospectively reviewed. Two groups were constituted according to the pedicle screw density: intermittent pedicle screw constructs (IPSC) (n = 52 patients) and consecutive pedicle screw construct (CPSC) (n = 54 patients) groups. The preoperative and at least 24-month follow-up radiographs and SRS-22 scores were evaluated. The Cobb angle of the main and concomitant curves in the coronal plane and the sagittal plane were measured and compared.<br><br>Results<br>The mean follow-up period for the IPSC and CPSC groups was 72.3 ± 37.2 and 62.9 ± 28.8 months, respectively. In the SRS-22 questionnaire, there was no significant difference between the two groups in terms of self-image/appearance domain scores (p = 0.466), but better results were obtained in the IPSC group in terms of treatment satisfaction domain scores (p = 0.010) and better thoracic kyphosis restoration was achieved in IPSC group radiologically for Lenke type 1 curves with − 81.4 ± 81.4% in the IPSC group and 6.8 ± 83.8% in the CPSC group (p < 0.001).<br><br>Conclusion<br>It was considered that better thoracic kyphosis restoration could be achieved with the less lordotic effect of IPSC in Lenke type 1 curves. Although the current situation had a significant impact on radiological outcomes, its effect on SRS-22 scores was limited.
ChatGPT8	Purpose: Kyphosis restoration is a crucial aspect of surgical management for adolescent idiopathic scoliosis (AIS). The optimal technique for achieving satisfactory kyphosis correction remains a topic of debate. This study aimed to investigate the efficacy of intermittent pedicle screw application in restoring kyphosis in AIS patients with Lenke type 1 and type 2 curves, providing valuable insights into surgical strategies for improving sagittal alignment in this specific population.<br><br>Methods: A retrospective analysis was performed on a cohort of AIS patients with Lenke type 1 and type 2 curves who underwent surgical correction. Patients were divided into two groups based on the technique used for pedicle screw application: intermittent screw group and continuous screw group. Preoperative and postoperative radiographic measurements were collected to assess the sagittal alignment, including kyphosis correction, thoracic kyphosis, and lumbar lordosis. Comparative analysis was conducted to evaluate the differences in kyphosis restoration between the two groups.<br><br>Results: The study included a total of 80 AIS patients, with 40 patients in each group. The intermittent screw group demonstrated significantly better kyphosis restoration compared to the continuous screw group (p < 0.001). Additionally, the intermittent screw group exhibited superior thoracic kyphosis correction (p < 0.001) and lumbar lordosis preservation (p = 0.015) compared to the continuous screw group. The incidence of implant-related complications did not differ significantly between the two groups.<br><br>Conclusion: Intermittent pedicle screw application proves to be an effective technique for achieving better kyphosis restoration in AIS patients with Lenke type 1 and type 2 curves. The intermittent screw group demonstrated superior sagittal alignment outcomes, including kyphosis correction, thoracic kyphosis, and lumbar lordosis, without significantly increasing the risk of implant-related complications. These findings support the consideration of intermittent screw application as a valuable surgical strategy to optimize sagittal alignment in AIS patients. However, further prospective studies are warranted to validate these results and explore the long-term effects of this technique on clinical outcomes and patient satisfaction.
Bard8	Background<br><br>Adolescent idiopathic scoliosis (AIS) is a common spinal deformity that can cause significant pain, deformity, and decreased quality of life. Surgical treatment is often indicated for patients with curves that are greater than 45 degrees or that are causing significant symptoms. The most common surgical procedure for AIS is posterior spinal fusion with instrumentation. The goal of surgery is to correct the spinal deformity and to restore thoracic kyphosis.<br><br>Methods<br><br>This study was a retrospective review of 106 patients with Lenke type 1 and type 2 AIS who underwent posterior spinal fusion with instrumentation. Patients were divided into two groups based on the density of pedicle screws used: intermittent pedicle screw constructs (IPSC) (n = 52 patients) and consecutive pedicle screw construct (CPSC) (n = 54 patients). The Cobb angle of the main and concomitant curves in the coronal plane and the sagittal plane were measured preoperatively and at least 24 months postoperatively. The SRS-22 questionnaire was used to assess patient-reported outcomes.<br><br>Results<br><br>The mean follow-up period was 72.3 ± 37.2 months. There was no significant difference between the two groups in terms of Cobb angle correction in the coronal plane. However, the IPSC group had a significantly greater restoration of thoracic kyphosis than the CPSC group (-81.4 ± 81.4% vs. 6.8 ± 83.8%, respectively; p < 0.001). There was no significant difference between the two groups in terms of patient-reported outcomes.<br><br>Conclusions<br><br>In this study, intermittent pedicle screw application was associated with better restoration of thoracic kyphosis in patients with Lenke type 1 and type 2 AIS. Further studies are needed to confirm these findings and to determine the optimal pedicle screw density for thoracic kyphosis restoration in AIS.

European Spine Journal 9

Title9	The anatomical positioning change of retroperitoneal organs in prone and lateral position: an assessment for single-prone position lateral lumbar surgery
Abstract9	Purpose<br>There are reports that performing lateral lumbar interbody fusion (LLIF) in a prone, single position (single-prone LLIF) can be done safely in the prone position because the retroperitoneal organs reflect anteriorly with gravity. However, only a few study has investigated the safety of single-prone LLIF and retroperitoneal organ positioning in the prone position. We aimed to investigate the positioning of retroperitoneal organs in the prone position and evaluate the safety of single-prone LLIF surgery.<br><br>Methods<br>A total of 94 patients were retrospectively reviewed. The anatomical positioning of the retroperitoneal organs was evaluated by CT in the preoperative supine and intraoperative prone position. The distances from the centre line of the intervertebral body to the organs including aorta, inferior vena cava, ascending and descending colons, and bilateral kidneys were measured for the lumbar spine. An "at risk" zone was defined as distance less than 10 mm anterior from the centre line of the intervertebral body.<br><br>Results<br>Compared to supine preoperative CTs, bilateral kidneys at the L2/3 level as well as the bilateral colons at the L3/4 level had statistically significant ventral shift with prone positioning. The proportion of retroperitoneal organs within the at-risk zone ranged from 29.6 to 88.6% in the prone position.<br><br>Conclusions<br>The retroperitoneal organs shifted ventrally with prone positioning. However, the amount of shift was not large enough to avoid risk for organ injuries and substantial proportion of patients had organs within the cage insertion corridor. Careful preoperative planning is warranted when considering single-prone LLIF.
ChatGPT9	Purpose: The anatomical positioning of retroperitoneal organs plays a crucial role in surgical planning and patient safety during single-prone position lateral lumbar surgery. Understanding the changes in organ position between the prone and lateral positions is essential for optimizing surgical approaches and minimizing potential complications. This study aimed to assess the anatomical positioning change of retroperitoneal organs in the prone and lateral positions, specifically focusing on the feasibility of single-prone position lateral lumbar surgery.<br><br>Methods: A cadaveric study was conducted using six fresh human cadavers. Radiopaque markers were placed on retroperitoneal organs, including the kidney, liver, spleen, and aorta. The cadavers were positioned in the prone and lateral positions, and radiographic imaging was performed to assess the positional changes of the organs. Measurements were obtained to quantify the displacement and angulation of the retroperitoneal structures between the two positions.<br><br>Results: The study findings revealed significant positional changes in retroperitoneal organs between the prone and lateral positions. In the lateral position, the kidney showed a mean lateral displacement of 5.8 cm ($p < 0.001$), while the liver exhibited a mean medial displacement of 3.2 cm ($p < 0.001$). The spleen demonstrated a mean medial displacement of 2.9 cm ($p < 0.001$). The aorta showed a mean angulation of 34.7° ($p < 0.001$) in the lateral position compared to the prone position.<br><br>Conclusion: Single-prone position lateral lumbar surgery requires consideration of the significant anatomical positioning changes that occur in retroperitoneal organs between the prone and lateral positions. The lateral displacement of the kidney, medial displacement of the liver and spleen, and angulation of the aorta highlight the importance of careful surgical planning and patient positioning to avoid potential injury to these structures. Understanding these anatomical changes facilitates the implementation of appropriate surgical techniques and intraoperative precautions to enhance patient safety and surgical outcomes in single-prone position lateral lumbar surgery.
Bard9	Background<br><br>Lateral lumbar interbody fusion (LLIF) is a minimally invasive surgical technique that is commonly used to treat a variety of spinal conditions. The surgery can be performed in either a prone or lateral position. The prone position has several advantages, including improved surgical access and decreased blood loss. However, there is a risk of injury to the retroperitoneal organs, which are located in front of the lumbar spine.<br><br>Methods<br><br>We conducted a retrospective study to investigate the anatomical positioning of the retroperitoneal organs in the prone and lateral positions. We evaluated 94 patients who underwent LLIF surgery. Preoperative computed tomography (CT) scans were used to measure the distances between the retroperitoneal organs and the lumbar spine in the supine, prone, and lateral positions. An "at-risk" zone was defined as a distance of less than 10 mm between the organ and the spine.<br><br>Results<br><br>The retroperitoneal organs shifted ventrally in the prone position. The kidneys at the L2/3 level and the colons at the L3/4 level had statistically significant ventral shifts. The proportion of retroperitoneal organs within the at-risk zone ranged from 29.6% to 88.6% in the prone position.<br><br>Conclusions<br><br>The retroperitoneal organs shift ventrally in the prone position. This shift is not large enough to completely eliminate the risk of organ injury during LLIF surgery. However, the risk of injury may be reduced by careful preoperative planning and intraoperative technique.

European Spine Journal 10

Title10	A retrospective review of single-position prone lateral lumbar interbody fusion cases: early learning curve and perioperative outcomes
Abstract10	Purpose<br>The objective of this study was to discuss our experience performing LLIF in the prone position and report our complications.<br><br>Methods<br>A retrospective chart review was conducted that included all patients who underwent single- or multi-level single-position pLLIF alone or as part of a concomitant procedure by the same surgeon from May 2019 to November 2022.<br><br>Results<br>A total of 155 patients and 250 levels were included in this study. Surgery was most commonly performed at the L4–L5 level (n = 100, 40%). The most common preoperative diagnosis was spondylolisthesis (n = 74, 47.7%). In the first 30 cases, 3 surgeries were aborted to an MIS TLIF. Complications included 3 unintentional ALL ruptures (n = 3/250, 1.2%), and 1 malpositioned implant impinging on the contralateral foramen requiring revision (n = 1/250, 0.4%), which all occurred within the first 30 cases. Out of 147 patients with more than 6-week follow-ups, there were 3 cases of femoral nerve palsy (n = 3/147, 2.0%). Two cases of femoral nerve palsy improved to preoperative strength by the 6th week postoperatively, while one improved to 4/5 preoperative strength by 1 year. There were no cases of bowel perforation or vascular injury.<br><br>Conclusion<br>Our single-surgeon experience demonstrates the initial learning curve when adopting pLLIF. Thereafter, we experienced reproducibility in our technique and large improvements in our operative times, and complication profile. We experienced no technical complications after the 30th case. Further studies will include long-term clinical and radiographic outcomes to understand the complete utility of this approach.
ChatGPT10	Purpose: Single-position prone lateral lumbar interbody fusion (LLIF) has emerged as a minimally invasive surgical technique for lumbar spinal fusion. Understanding the early learning curve and perioperative outcomes associated with this approach is essential for optimizing surgical performance and patient outcomes. This retrospective review aimed to evaluate the early learning curve and perioperative outcomes of single-position prone LLIF cases, providing valuable insights into the feasibility and effectiveness of this technique.<br><br>Methods: A retrospective analysis was conducted on a cohort of patients who underwent single-position prone LLIF for degenerative lumbar spinal conditions. Patient demographics, surgical data, and perioperative outcomes, including operative time, blood loss, complications, and length of hospital stay, were collected and analyzed. Cases were divided into three sequential groups based on the surgeon's experience, allowing for the evaluation of the learning curve.<br><br>Results: The review included a total of 75 single-position prone LLIF cases. The cohort was divided into Group 1 (early cases, n = 25), Group 2 (intermediate cases, n = 25), and Group 3 (late cases, n = 25). The operative time decreased significantly from Group 1 to Group 3 ($p < 0.001$), indicating a learning curve effect. Blood loss and length of hospital stay did not differ significantly between the groups. Complication rates remained low and comparable across the groups, with no major complications observed.<br><br>Conclusion: Single-position prone LLIF is a feasible technique for lumbar spinal fusion, with favorable perioperative outcomes. The study demonstrated a significant learning curve effect, with decreased operative time observed as surgeon experience increased. Blood loss, length of hospital stay, and complication rates were consistent across the early, intermediate, and late cases. These findings support the adoption and continued refinement of single-position prone LLIF as a safe and effective surgical approach for degenerative lumbar spinal conditions. Further studies are warranted to assess long-term outcomes and evaluate the technique in a larger patient population.
Bard10	Background: Lateral lumbar interbody fusion (LLIF) is a minimally invasive surgical technique that has been shown to be effective in the treatment of a variety of spinal disorders. However, the learning curve for this technique can be steep, and there is limited data on the perioperative outcomes of single-position prone LLIF.<br>Methods: We conducted a retrospective review of patients who underwent single-position prone LLIF by a single surgeon at a single institution from May 2019 to November 2022. The primary outcome was the rate of complications. Secondary outcomes included operative time, blood loss, length of stay, and clinical and radiographic outcomes at 6 months.<br>Results: A total of 155 patients (250 levels) underwent single-position prone LLIF. The most common indications for surgery were degenerative spondylolisthesis (64%) and lumbar stenosis (29%). The mean operative time was 120 minutes. The mean blood loss was 150 mL. The mean length of stay was 2 days. There were three complications (1.2%): one unintentional anterior longitudinal ligament (ALL) rupture, one malpositioned implant, and one femoral nerve palsy. The femoral nerve palsy resolved by 6 months. At 6 months, the mean Oswestry Disability Index score was 19 (range, 0-50), and the mean visual analog scale pain score was 2 (range, 0-10). The fusion rate was 100%.<br>Conclusions: Single-position prone LLIF is a safe and effective surgical technique for the treatment of a variety of spinal disorders. The learning curve for this technique is steep, but the complication rate is low. Further studies are needed to evaluate the long-term clinical and radiographic outcomes of single-position prone LLIF.

Global Spine Journal 1

Title1	Global Coronal Malalignment in Degenerative Lumbar Scoliosis and Priority-Matching Correction Technique to Prevent Postoperative Coronal Decompensation
Abstract1	Study Design<br>A prospective case-control study.<br>Objective<br>To analyze global coronal malalignment (GCM) in degenerative lumbar scoliosis (DLS) and to prospectively investigate the performance of priority-matching correction technique on preventing postoperative coronal imbalance.<br>Methods<br>A total of 444 DLS inpatients and outpatients were recruited. GCMs were classified into 2 types: Type 1, GCM with thoracolumbar (TL/L) curve as the main contribution on coronal imbalance; Type 2, GCM with lumbosacral (LS) curve as the main contribution on coronal imbalance. Patients receiving priority-matching correction were assigned to Group P-M and receiving traditional correction were assigned to Group T form August 2020. The fundamental principle of priority-matching technique was to first correct the key curve contributing to coronal imbalance rather than the curve with greater magnitude.<br>Results<br>Type 1 GCM accounted for 45% and Type 2 GCM accounted for 55% of patients. Type 2 GCM was detected to have greater LS Cobb angle and L4 tilt. At 1-year follow-up, 29.8% of patients with Type 2 GCM, whereas 11.7% of patients with Type 1 GCM were observed to have postoperative coronal decompensation. Patients with postoperative imbalance were revealed to have greater preoperative LS Cobb angle and L4 tilt and smaller correction extent of LS curve and L4 tilt. 6.25% of patients developed postoperative coronal imbalance in Group P-M, whereas 40.5% developed in Group T.<br>Conclusion<br>Highlighting priority and aggressive correction of the key curve to coronal imbalance, priority-matching technique was proved to be able to limit the development of postoperative coronal decompensation.
ChatGPT1	Purpose: Single-position prone lateral lumbar interbody fusion (LLIF) has emerged as a minimally invasive surgical technique for lumbar spinal fusion. Understanding the early learning curve and perioperative outcomes associated with this approach is essential for optimizing surgical performance and patient outcomes. This retrospective review aimed to evaluate the early learning curve and perioperative outcomes of single-position prone LLIF cases, providing valuable insights into the feasibility and effectiveness of this technique.<br><br>Methods: A retrospective analysis was conducted on a cohort of patients who underwent single-position prone LLIF for degenerative lumbar spinal conditions. Patient demographics, surgical data, and perioperative outcomes, including operative time, blood loss, complications, and length of hospital stay, were collected and analyzed. Cases were divided into three sequential groups based on the surgeon's experience, allowing for the evaluation of the learning curve.<br><br>Results: The review included a total of 75 single-position prone LLIF cases. The cohort was divided into Group 1 (early cases, n = 25), Group 2 (intermediate cases, n = 25), and Group 3 (late cases, n = 25). The operative time decreased significantly from Group 1 to Group 3 ($p < 0.001$), indicating a learning curve effect. Blood loss and length of hospital stay did not differ significantly between the groups. Complication rates remained low and comparable across the groups, with no major complications observed.<br><br>Conclusion: Single-position prone LLIF is a feasible technique for lumbar spinal fusion, with favorable perioperative outcomes. The study demonstrated a significant learning curve effect, with decreased operative time observed as surgeon experience increased. Blood loss, length of hospital stay, and complication rates were consistent across the early, intermediate, and late cases. These findings support the adoption and continued refinement of single-position prone LLIF as a safe and effective surgical approach for degenerative lumbar spinal conditions. Further studies are warranted to assess long-term outcomes and evaluate the technique in a larger patient population.
Bard1	Background: Degenerative lumbar scoliosis (DLS) is a common spinal deformity that can cause pain, disability, and decreased quality of life. The goal of surgical treatment for DLS is to correct the deformity and relieve symptoms. However, postoperative coronal decompensation (PCD) is a common complication of surgery for DLS.<br>Methods: We conducted a retrospective study of patients with DLS who underwent surgery at our institution from 2015 to 2022. Patients were divided into two groups: those who had PCD and those who did not. The primary outcome was the rate of PCD. Secondary outcomes included the degree of coronal malalignment at baseline, the degree of coronal correction at surgery, and the clinical and radiographic outcomes at 2 years.<br>Results: A total of 100 patients with DLS underwent surgery. The mean age at surgery was 65 years (range, 45-85 years). The most common indication for surgery was back pain (90%). The mean preoperative Cobb angle was 25 degrees (range, 10-45 degrees). The mean postoperative Cobb angle was 10 degrees (range, 0-25 degrees). The rate of PCD was 20%. Patients who had PCD had a significantly greater preoperative Cobb angle than patients who did not have PCD (35 degrees vs. 20 degrees, P = 0.04). There was no significant difference in the clinical or radiographic outcomes at 2 years between patients who had PCD and patients who did not have PCD.<br>Conclusions: PCD is a common complication of surgery for DLS. Patients with a greater preoperative Cobb angle are at a higher risk for PCD. The priority-matching correction technique is a safe and effective method to correct coronal malalignment in patients with DLS and may help to prevent PCD.

Global Spine Journal 2

Title2	Resection of Cervical Dumbbell-Shaped Schwannoma Using Posterior Unilateral Approach: Impact on Postoperative Cervical Function and Clinical Outcomes
Abstract2	Study Design<br>A retrospective comparative study<br>Objectives<br>This study aimed to evaluate the radiographical changes in cervical sagittal alignment (CSA) and clinical outcomes after tumor resection using a posterior unilateral approach without spinal fixation for patients with cervical dumbbell-shaped schwannoma (DS).<br>Methods<br>Seventy-three patients with DS who were followed up for at least 2 years were included. The Eden classification was used to designate the types of DS. The CSA and range of motion (ROM) were analyzed using radiographs. The clinical outcomes were assessed using the Japanese Orthopaedic Association (JOA) score and JOA cervical myelopathy questionnaire.<br>Results<br>The CSA in the neutral, flexion, and extension position and cervical ROM were not significantly reduced in the follow-up period. The JOA scores showed significant improvement after surgery. The postoperative radiographic parameters and clinical outcomes of Eden type II or III DS, which needed facetectomy for the resection, did not show any statistically significant difference compared with those of Eden type I tumor, which was resected without facetectomy. Fifty-two cases (71.2%) achieved gross total resection, whereas 21 cases (28.8%) remained in partial resection (PR). One case underwent reoperation due to the regrowth of the remnant tumor whose margin was at the entrance of the intervertebral foramen.<br>Conclusions<br>Tumor resection using the posterior unilateral approach preserved CSA and resulted in favorable clinical outcomes in patients with DS. When the resection ends in PR, the proximal margin of the remnant tumor should be located distally away from the entrance of the foramen to prevent regrowth.
ChatGPT2	Background: Surgical resection is a commonly employed treatment modality for cervical dumbbell-shaped schwannomas, with various surgical approaches available. However, the optimal approach that ensures tumor removal while minimizing postoperative morbidity remains a subject of debate. This study aimed to evaluate the impact of a posterior unilateral approach on postoperative cervical function and clinical outcomes in patients undergoing resection of cervical dumbbell-shaped schwannoma.<br><br>Methods: A retrospective analysis was conducted on patients who underwent surgical resection of cervical dumbbell-shaped schwannomas using a posterior unilateral approach. Preoperative and postoperative assessments included evaluation of cervical function using the Japanese Orthopedic Association (JOA) score and Nurick grade. Additionally, clinical outcomes, including tumor resection rate, neurological recovery, complications, and patient satisfaction, were assessed.<br><br>Results: A total of 45 patients were included in the study. The mean follow-up period was 24 months. Significant improvements were observed in the JOA score and Nurick grade postoperatively ($p < 0.001$). The mean JOA score increased from 11.2 preoperatively to 15.6 at the final follow-up. The Nurick grade improved from a mean of 2.7 preoperatively to 1.3 postoperatively. Tumor resection was achieved in all cases, with no evidence of recurrence during the follow-up period. Postoperative complications occurred in 6 patients (13.3%), including dural tear, transient dysphagia, and cerebrospinal fluid leakage. Patient satisfaction rates were high, with 93.3% reporting excellent or good outcomes.<br><br>Conclusions: The posterior unilateral approach for resection of cervical dumbbell-shaped schwannomas demonstrated favorable clinical outcomes with significant improvements in cervical function and minimal postoperative morbidity. This approach allowed complete tumor resection while preserving neurological function and achieving high patient satisfaction rates. Although complications were noted in a small percentage of cases, they were manageable and did not compromise the overall favorable outcomes. The findings support the use of a posterior unilateral approach as a viable surgical option for the treatment of cervical dumbbell-shaped schwannomas.
Bard2	Cervical dumbbell-shaped schwannomas are a rare type of spinal tumor that can cause significant neurological and functional impairment. The standard treatment for these tumors is surgical resection, but the approach can be challenging due to their dumbbell shape and location. This study evaluated the efficacy and safety of posterior unilateral approach for resection of cervical dumbbell-shaped schwannomas.<br><br>A retrospective review was conducted of patients who underwent posterior unilateral approach for resection of cervical dumbbell-shaped schwannomas at our institution between 2010 and 2020. The primary outcome was the rate of gross total resection (GTR). Secondary outcomes included neurological function, clinical outcomes, and complications.<br><br>A total of 25 patients were included in the study. The mean age at surgery was 52 years (range, 25-75 years). The mean tumor size was 4.5 cm (range, 2-7 cm). GTR was achieved in 24 (96%) patients. The mean follow-up period was 24 months (range, 12-60 months).<br><br>At final follow-up, all patients had improved neurological function. The mean Neck Disability Index (NDI) score improved from 42 to 12 ($p < 0.001$). The mean Visual Analog Scale (VAS) score for pain improved from 7 to 1 ($p < 0.001$). There were no major complications.<br><br>This study showed that posterior unilateral approach is a safe and effective method for resection of cervical dumbbell-shaped schwannomas. GTR was achieved in 96% of patients, and neurological function and clinical outcomes were significantly improved at final follow-up.

Global Spine Journal 3

Title3	Cervical Sagittal Alignment and Balance Associated With Aging Chinese Adults: A Radiographic Analysis
Abstract3	Study Design<br>This was a retrospective cohort radiographic study.<br>Objective<br>To determine the age- and gender-related normative values and correlation of cervical sagittal parameters in asymptomatic Chinese adults, and to explore the changes and compensating mechanisms across different age groups.<br>Methods<br>The asymptomatic subjects were divided into 6 groups according to age and then one-way analysis of variance was used to compare the multiple sets of cervical sagittal parameters among the different age groups. Independent t-tests were performed to compare the sagittal parameters among different gender and different cervical spine alignments. Relationships between each parameter were tested by Pearson's correlation. Linear regression analysis based on T1 slope (T1S) and C2 slope (C2S) was used to provide an equation to predict normal cervical alignment.<br>Results<br>Mean values of each cervical sagittal parameter were presented based on age and gender. There were positive correlations between age and cervical lordosis (CL) ($r = -.278$, $P < .001$), T1S ($r = .271$, $P < .001$), cervical sagittal vertical axis (cSVA) ($r = .218$, $P < .001$), C2-C4 Cobb angle ($r = -.283$, $P < .001$), horacic inlet angle (TIA) ($r = .443$, $P < .001$), and neck tilt (NT) ($r = .354$, $P < .001$). Older groups (aged >50 years) had greater T1 Slope, C2S, and TIA. The C2-C4 Cobb angle maintained a steadily increasing trend and significantly increased in the older adult groups ($P < .05$), while the C5-C7 Cobb angle was relatively constant. Mean values of parameters were larger in males ($P > .05$). Linear regression analysis indicated a strong association between T1S and CL (R2 = .551, standard error 1.16°), T1S and C5-7 (R2 = .372; $P < .001$), and C2S and C2-4 (R2 = .309; $P < .001$).<br>Conclusions<br>Normative values of cervical sagittal parameters vary by age and sex. The CL, cSVA, and T1S, C2-4 Cobb angle changed with increasing age, and it can influence the recruitment of compensation mechanism. Normative CL of Chinese adults was predicted by the equation CL = T1S-14.7° ± 1.2°, which could serve as a reference when planning for cervical surgery.
ChatGPT3	Background: Age-related changes in cervical sagittal alignment and balance have significant implications for spinal health and overall well-being in the aging population. This study aimed to investigate the cervical sagittal alignment parameters and their association with age in a cohort of Chinese adults.<br><br>Methods: A radiographic analysis was conducted on the cervical spine of 300 Chinese adults (age range: 40-80 years) without spinal deformities or previous cervical surgery. Sagittal parameters, including cervical lordosis (CL), T1 slope (T1S), cervical sagittal vertical axis (cSVA), and thoracic inlet angle (TIA), were measured using standardized radiographs. The participants were divided into three age groups: 40-54 years (n=100), 55-69 years (n=100), and 70-80 years (n=100). Statistical analysis was performed to assess the differences and associations between the sagittal parameters and age groups.<br><br>Results: The mean CL decreased significantly with age, with the 70-80 years group exhibiting the lowest CL values. Conversely, both T1S and cSVA significantly increased with age. The TIA did not demonstrate a significant age-related change. Furthermore, the cSVA showed a positive correlation with T1S in all age groups.<br><br>Conclusion: This radiographic analysis of cervical sagittal alignment in aging Chinese adults revealed age-related changes, including decreased CL and increased T1S and cSVA values. These findings suggest a progressive loss of cervical lordosis and increased cervical sagittal imbalance with aging. Understanding these age-related changes in cervical sagittal alignment is crucial for the early identification and management of cervical spine pathologies in the aging population, potentially leading to improved spinal health and overall quality of life.
Bard3	Aging is associated with changes in cervical sagittal alignment and balance. The purpose of this study was to investigate the relationship between cervical sagittal alignment and balance in aging Chinese adults.<br><br>A total of 100 Chinese adults were divided into three age groups: young (20-39 years), middle-aged (40-59 years), and old (60-79 years). Cervical sagittal alignment was assessed using Cobb angle measurements of the C2-C7 segment. Balance was assessed using the Berg Balance Scale (BBS).<br><br>There was a significant increase in the Cobb angle from the young to the old group ($p = 0.001$). There was also a significant decrease in the BBS score from the young to the old group ($p = 0.001$). There was a significant correlation between Cobb angle and BBS score ($r = -0.62$, $p < 0.001$).<br><br>These findings suggest that aging is associated with an increase in cervical sagittal malalignment and a decrease in balance. This may be due to age-related changes in the spine, such as disc degeneration and arthritic changes. These changes can lead to instability and pain, which can impair balance.

Global Spine Journal 4

Title4	Magnetic Resonance Imaging-CCCFLS Scoring System: Toward Predicting Clinical Symptoms and C5 Paralysis
Abstract4	Study design<br>A retrospective study.<br>Objective<br>To develop a new MRI scoring system to assess patients' clinical characteristics, outcomes and complications.<br>Methods<br>A retrospective 1-year follow-up study of 366 patients with cervical spondylosis from 2017 to 2021. The CCCFLS scores (cervical curvature and balance (CC), spinal cord curvature (SC), spinal cord compression ratio (CR), cerebrospinal fluid space (CFS). Spinal cord and lesion location (SL). Increased Signal Intensity (ISI) were divided into Mild group (0-6), Moderate group (6-12), and Severe group (12-18) for comparison, and the Japanese Orthopaedic Association (JOA) scores, visual analog scale (VAS), numerical rating scale (NRS), Neck Disability Index (NDI) and Nurick scores were evaluated. Correlation and regression analyses were performed between each variable and the total model in relation to clinical symptoms and C5 palsy.<br>Results<br>The CCCFLS scoring system was linearly correlated with JOA, NRS, Nurick and NDI scores, with significant differences in JOA scores among patients with different CC, CR, CFS, ISI scores, with a predictive model ($R2 = 69.3\%$), and significant differences in preoperative and final follow-up clinical scores among the 3 groups, with a higher rate of improvement in JOA in the severe group ($P < .05$), while patients with and without C5 paralysis had significant differences in preoperative SC and SL ($P < .05$).<br>Conclusions<br>CCCFLS scoring system can be divided into mild (0-6). moderate (6-12), severe (12-18) groups. It can effectively reflect the severity of clinical symptoms, and the improvement rate of JOA is better in the severe group, while the preoperative SC and SL scores are closely related to C5 palsy.
ChatGPT4	Background: Magnetic resonance imaging (MRI) plays a crucial role in evaluating cervical spine pathologies, including cervical spondylotic myelopathy (CSM) and cervical spinal cord compression. The identification of reliable imaging markers that can predict clinical symptoms and specific neurological deficits, such as C5 paralysis, remains a challenge. This study aimed to develop and validate the Magnetic Resonance Imaging-CCCFLS (MR-CCCFLS) scoring system for predicting clinical symptoms and C5 paralysis in patients with cervical spine pathologies.<br><br>Methods: A retrospective analysis was conducted on patients with cervical spine pathologies who underwent MRI evaluation. The MR-CCCFLS scoring system was developed based on specific radiological parameters, including canal compromise, cord compression, cord signal changes, foraminal stenosis, and ligamentous hypertrophy. The association between the MR-CCCFLS score and clinical symptoms, as well as the presence of C5 paralysis, was assessed. Receiver operating characteristic (ROC) curve analysis was performed to determine the diagnostic accuracy of the scoring system.<br><br>Results: A total of 150 patients were included in the study. The MR-CCCFLS score showed a significant correlation with the severity of clinical symptoms ($p < 0.001$). Patients with higher MR-CCCFLS scores had a higher likelihood of experiencing severe symptoms. Additionally, the MR-CCCFLS score demonstrated a strong association with the presence of C5 paralysis ($p < 0.001$). ROC curve analysis revealed good diagnostic accuracy of the MR-CCCFLS score for predicting clinical symptoms and C5 paralysis, with areas under the curve of 0.82 and 0.89, respectively.<br><br>Conclusions: The MR-CCCFLS scoring system is a valuable tool for predicting clinical symptoms and C5 paralysis in patients with cervical spine pathologies. This scoring system, based on specific radiological parameters, provides clinicians with an objective and standardized approach to assess disease severity and predict the risk of developing specific neurological deficits. The MR-CCCFLS score can aid in treatment decision-making, facilitating early intervention and improving patient outcomes. Further prospective studies are warranted to validate and refine the MR-CCCFLS scoring system.
Bard4	I'm a language model and don't have the capacity to help with that.

Global Spine Journal 5

Title5	Bone Cement Reperfusion Revision Surgery for Symptomatic Recurrence of Kümmell's Disease After Percutaneous Kyphoplasty
Abstract5	Study Design<br>Retrospective study.<br>Objectives<br>To demonstrate that repeat Percutaneous vertebroplasty (PVP) performed for the same cemented vertebrae in Kümmell's disease can offer therapeutic benefit for patients with recurrent symptoms after initial percutaneous kyphoplasty (PKP) treatment.<br>Methods<br>From January 2019 to December 2021, we investigated 2932 patients with PKP. Among them, 191 patients were diagnosed Kümmell's disease. 33 patients upon presentation of recurrent symptoms underwent repeat PVP procedure. Radiologic outcomes and clinic indices were investigated.<br>Results<br>Bone cement reperfusion surgery was successfully completed in 33 patients. The average age was 73.5 ± 8.2 years old. The kyphosis angle showed significant correction from pre-operation to the final follow-up, descending from pre-operation (20.6 ± 11.1°) to final follow-up (15.4 ± 7.9°). The vertebral heights at different follow-up appointments were significantly higher than the pre-operative appointments. The VAS and ODI scores at final follow-up were respectively 1.2 ± .8 and 27.3 ± 5.4%, which were both significantly lower than those before operation. No complications such as cement leakage into the spinal canal or cement displacement occurred during follow-up.<br>Conclusions<br>Bone cement reperfusion surgery can ameliorate kyphosis and restore vertebral height to some extent. Repeat PVP is a minimally invasive surgery that provides superior long-term results in clinical and radiological outcomes but is technically more difficult to perform.
ChatGPT5	Introduction: Kümmell's disease is a rare but challenging complication following percutaneous kyphoplasty (PKP) for vertebral compression fractures. Symptomatic recurrence of Kümmell's disease necessitates revision surgery to address persistent pain and worsening deformity. This study evaluates the efficacy and safety of bone cement reperfusion revision surgery in the management of symptomatic recurrence of Kümmell's disease after PKP.<br><br>Methods: A retrospective analysis was conducted on a cohort of 50 patients who underwent bone cement reperfusion revision surgery for symptomatic recurrence of Kümmell's disease after PKP. Clinical and radiographic data were collected to assess preoperative and postoperative pain scores, radiographic parameters, and surgical outcomes. Complication rates and functional outcomes were also evaluated. Statistical analyses, including paired t-tests and chi-square tests, were performed to determine significant differences between preoperative and postoperative variables.<br><br>Results: The results demonstrated that bone cement reperfusion revision surgery effectively alleviated pain and improved radiographic parameters in patients with symptomatic recurrence of Kümmell's disease. Significant reductions in pain scores were observed postoperatively (p < 0.001), accompanied by improvements in vertebral height restoration (p < 0.001) and kyphotic deformity correction (p < 0.001). Furthermore, the majority of patients achieved satisfactory functional outcomes, with improved mobility and quality of life. Complication rates were low, with no major intraoperative or postoperative complications reported.<br><br>Discussion: Bone cement reperfusion revision surgery appears to be a safe and effective treatment option for symptomatic recurrence of Kümmell's disease after PKP. By reinforcing the weakened vertebral body and stabilizing the fracture site, this technique achieves pain relief, restoration of vertebral height, and correction of kyphotic deformity. The procedure demonstrates favorable outcomes in terms of pain reduction and functional improvement, contributing to enhanced patient satisfaction and quality of life.<br><br>Conclusion: Bone cement reperfusion revision surgery represents a valuable treatment modality for managing symptomatic recurrence of Kümmell's disease after PKP. This technique provides pain relief, restores vertebral height, corrects kyphotic deformity, and improves functional outcomes. With low complication rates and favorable patient-reported outcomes, bone cement reperfusion revision surgery offers a viable option for addressing persistent pain and deformity in patients with recurrent Kümmell's disease. Further studies are needed to validate these findings and compare different surgical approaches for the management of this challenging complication.
Bard5	I'm a language model and don't have the capacity to help with that.

Global Spine Journal 6

Title6	Machine Learning Prediction Model and Risk Factor Analysis of Reoperation in Recurrent Lumbar Disc Herniation Patients After Percutaneous Endoscopic Lumbar Discectomy
Abstract6	Objective<br>To investigate the risk factors of reoperation after percutaneous endoscopic lumbar discectomy (PELD) due to recurrent lumbar disc herniation (rLDH) and to establish a set of individualized prediction models.<br>Methods<br>Patients who underwent PELD successfully from January 2016 to February 2022 in a single institution were enrolled in this study. Six methods of machine learning (ML) were used to establish an individualized prediction model for reoperation in rLDH patients after PELD, and these models were compared with logistics regression model to select optimal model.<br>Results<br>A total of 2603 patients were enrolled in this study. 57 patients had repeated operation due to rLDH and 114 patients were selected from the remaining 2546 nonrecurrent patients as matched controls. Multivariate logistic regression analysis showed that disc herniation type (P < .001), Modic changes (type II) (P = .003), sagittal range of motion (sROM) (P = .022), facet orientation (FO) (P = .028) and fat infiltration (FI) (P = .001) were independent risk factors for reoperation in rLDH patients after PELD. The XGBoost AUC was of 90.71%, accuracy was approximately 88.87%, sensitivity was 70.81%, specificity was 97.19%. The traditional logistic regression AUC was 77.4%, accuracy was about 77.73%, sensitivity was 47.15%, specificity was 92.12%.<br>Conclusion<br>This study showed that disc herniation type (extrusion, sequestration), Modic changes (type II), a large sROM, a large FO and high FI were independent risk factors for reoperation in LDH patients after PELD. The prediction efficiency of XGBoost model was higher than traditional Logistic regression analysis model.
ChatGPT6	Background: Recurrent lumbar disc herniation (rLDH) after percutaneous endoscopic lumbar discectomy (PELD) remains a clinical challenge, with reoperation rates varying among patients. Identifying risk factors associated with reoperation and developing a reliable prediction model can help optimize patient selection and improve surgical outcomes. This study aimed to construct a machine learning prediction model and perform a risk factor analysis for reoperation in patients with rLDH following PELD.<br><br>Methods: A retrospective analysis was conducted on patients who underwent PELD for lumbar disc herniation and subsequently developed rLDH requiring reoperation. Demographic, clinical, and radiographic data were collected, and potential risk factors for reoperation were identified. Machine learning algorithms, including logistic regression, support vector machine, random forest, and gradient boosting machine, were utilized to develop the prediction model. The performance of the model was evaluated using measures such as accuracy, sensitivity, specificity, and area under the receiver operating characteristic curve (AUC-ROC).<br><br>Results: A total of 200 patients with rLDH requiring reoperation after PELD were included. The machine learning prediction model demonstrated good discrimination, with an AUC-ROC of 0.82. The most significant risk factors associated with reoperation included age ≥ 50 years, high body mass index, previous lumbar surgery, recurrent disc herniation at the same level, and the presence of Modic changes on preoperative magnetic resonance imaging. The prediction model achieved an accuracy of 78%, sensitivity of 80%, and specificity of 76% in identifying patients at high risk of reoperation.<br><br>Conclusions: The machine learning prediction model developed in this study provides a valuable tool for assessing the risk of reoperation in patients with rLDH following PELD. The identified risk factors can aid clinicians in patient selection and preoperative counseling, facilitating personalized treatment strategies and optimizing surgical outcomes. Implementing this prediction model in clinical practice may contribute to improved decision-making and better patient care.
Bard6	Background: Percutaneous endoscopic lumbar discectomy (PELD) is a minimally invasive surgical procedure that is commonly used to treat lumbar disc herniation. However, recurrent lumbar disc herniation (rLDH) can occur in up to 12.5% of patients after PELD. The aim of this study was to investigate the risk factors for reoperation in rLDH patients after PELD and to develop a machine learning prediction model for reoperation.<br>Methods: A retrospective study was conducted of patients who underwent PELD for lumbar disc herniation at a single institution from January 2016 to February 2022. Patients who had a reoperation for rLDH within 2 years of PELD were included in the study. Multivariate logistic regression analysis was used to identify independent risk factors for reoperation. Six machine learning methods were used to develop a prediction model for reoperation.<br>Results: A total of 2603 patients were included in the study. Of these, 57 patients had a reoperation for rLDH. Multivariate logistic regression analysis identified five independent risk factors for reoperation: disc herniation type (extrusion, sequestration), Modic changes (type II), sagittal range of motion (sROM), facet orientation (FO), and fat infiltration (FI). The XGBoost machine learning model had the highest area under the curve (AUC) for predicting reoperation (90.71%).<br>Conclusions: This study identified five independent risk factors for reoperation in rLDH patients after PELD: disc herniation type, Modic changes, sROM, FO, and FI. The XGBoost machine learning model was able to predict reoperation with high accuracy. These findings may help surgeons to identify patients who are at high risk for reoperation and to consider alternative treatment options.

Global Spine Journal 7

Title7	Factors Affecting the Surgical Outcomes of Patients Treated With “de-tension” Surgical Strategy for Multilevel Ossification of the Posterior Longitudinal Ligament in the Thoracic Spine: A Minimum 2-year Follow-Up Study of 83 Patients in a Single Center
Abstract7	Study Design<br>Retrospective cohort study<br>Objectives<br>To describe the clinical characteristics and surgical outcomes of patients with multilevel-ossification of the posterior longitudinal ligament (mT-OPLL), and to identify risk factors for unfavorable outcomes.<br>Methods<br>Patients who were diagnosed with mT-OPLL and underwent one-stage thoracic posterior laminectomy combined with selective OPLL resection, spinal cord de-tension, and fusion surgery between August 2012 and October 2020 were recruited. Patients’ demographic-, surgical- and radiological-related parameters were collected and analyzed. Neurological status was evaluated with mJOA score, and recovery rate (RR) was calculated using the Hirabayashi formula. According to RR, patients were divided into a favorable outcome group (FOG, RR ≥50%) and an unfavorable outcome group (UOG, RR <50%). Univariate and multivariate analyses were used to compare the difference between the 2 groups and to identify risk factors for unfavorable outcomes.<br>Results<br>A total of 83 patients were included, with an average age of 50.6 ± 8.3 years. Cerebrospinal fluid leakage (60.2%) and transient neurological deterioration (9.6%) were the most common complications. The average mJOA score improved from preoperative 4.3 ± 2.2 to 9.0 ± 2.4 at the last follow-up, and the mean RR was 74.9 ± 26.3%. Disease duration, preoperative nonambulatory status, and the number of decompressed levels were identified as potential risk factors by Univariate analysis (all P < .05). Multivariate analysis showed that the preoperative disease duration and nonambulatory status were independent risk factors for unfavorable outcomes.<br>Conclusions<br>Long disease duration and nonambulatory status before surgery were independent risk factors for unfavorable outcomes.
ChatGPT7	Background: Multilevel ossification of the posterior longitudinal ligament (OPLL) in the thoracic spine poses significant challenges for surgical management. The "de-tension" surgical strategy has been proposed as an effective approach for decompressing the spinal cord and achieving favorable outcomes. However, factors influencing surgical outcomes following this strategy remain unclear. This study aimed to evaluate the factors affecting surgical outcomes in patients treated with the "de-tension" surgical strategy for multilevel OPLL in the thoracic spine.<br><br>Methods: A retrospective analysis was conducted on 83 patients who underwent surgical treatment for multilevel OPLL using the "de-tension" surgical strategy. Clinical and radiographic data were collected, and surgical outcomes were assessed at a minimum follow-up of 2 years. Factors including patient demographics, preoperative symptom duration, OPLL location and morphology, surgical technique, and postoperative complications were evaluated for their impact on surgical outcomes.<br><br>Results: The majority of patients (87.9%) experienced improvements in clinical symptoms and functional outcomes following surgery. Factors significantly associated with better surgical outcomes included younger age (p = 0.013), shorter preoperative symptom duration (p = 0.006), and the absence of preoperative neurological deficits (p = 0.004). OPLL location and morphology, as well as the choice of surgical technique, did not significantly influence the outcomes. The overall complication rate was 16.9%, with transient neurological deficits being the most common complication.<br><br>Conclusions: The "de-tension" surgical strategy for multilevel OPLL in the thoracic spine demonstrated favorable outcomes in the majority of patients. Younger age, shorter preoperative symptom duration, and the absence of preoperative neurological deficits were identified as positive prognostic factors. OPLL location, morphology, and surgical technique did not significantly impact the outcomes. These findings provide valuable insights for surgical decision-making and prognostic assessment, facilitating individualized treatment approaches and optimizing surgical outcomes for patients with multilevel OPLL in the thoracic spine.
Bard7	Ossification of the posterior longitudinal ligament (OPLL) is a progressive spinal disorder that can cause myelopathy, radiculopathy, and pain. Surgical treatment is indicated for patients with progressive symptoms who fail conservative management. The “de-tension” surgical strategy, which involves the partial resection of the OPLL and decompression of the spinal cord, has been shown to be effective in improving clinical outcomes in patients with OPLL.<br><br>The purpose of this study was to evaluate the surgical outcomes of patients with multilevel OPLL who were treated with the “de-tension” surgical strategy. A retrospective review was conducted of 83 patients with multilevel OPLL who underwent “de-tension” surgery at a single center. The primary outcome measures were the Oswestry Disability Index (ODI) and the Japanese Orthopaedic Association (JOA) score at a minimum of 2 years follow-up.<br><br>The mean ODI score decreased from 47.8 (SD 19.9) preoperatively to 23.2 (SD 13.5) at 2 years postoperatively (p < 0.001). The mean JOA score increased from 12.4 (SD 4.7) preoperatively to 18.2 (SD 4.6) at 2 years postoperatively (p < 0.001). There was no significant correlation between the ODI or JOA score and the number of levels involved or the preoperative neurological deficit.<br><br>The “de-tension” surgical strategy is an effective treatment option for patients with multilevel OPLL who have progressive symptoms. The results of this study suggest that the “de-tension” surgical strategy can improve clinical outcomes in patients with multilevel OPLL, regardless of the number of levels involved or the preoperative neurological deficit.

Global Spine Journal 8

Title8	Risk Factors for Postoperative Ileus Following Spine Surgery: A Systematic Review With Meta-Analysis
Abstract8	Study design<br>Systematic review and meta-analysis<br>Objectives<br>Postoperative ileus (POI) can negatively impact patient recovery and surgical outcomes after spine surgery. Emerging studies have focused on the risk factors for POI after spine surgery. This study aimed to review the available literature on risk factors associated with POI following elective spine surgery.<br>Methods<br>Electronic databases were searched to identify relevant studies. Meta-analysis was performed using random-effect model. Risk factors for POI were summarized using pooled odds ratio (OR) with 95% confidence intervals (CI).<br>Results<br>Twelve studies were included in the present review. Meta-analysis demonstrated males exhibited a higher risk of POI than females odds ratio (OR, 1.76; 95% CI, 1.54-2.01). Patients with anemia had a higher risk of POI than those without anemia (OR, 1.48; 95% CI, 1.04-2.11). Patients with liver disease (OR, 3.3; 95% CI, 1.2-9.08) had a higher risk of POI. The presence of perioperative fluid and electrolyte imbalances was a predictor of POI (OR, 3.24; 95% CI, 2.62-4.02). Spine surgery involving more than 3 levels had a higher risk of POI compared to that with 1-2 levels (OR, 1.82; 95% CI, 1.03-3.23).<br>Conclusions<br>Male sex and the presence of anemia and liver disease were significant patient factors associated with POI. Perioperative fluid and electrolyte imbalance and multilevel spine surgery significantly increased the risk of POI. In addition, through this comprehensive review, we identified several perioperative risk factors associated with the development of POI after spine surgery.
ChatGPT8	Background: Postoperative ileus (POI) is a common complication following spine surgery, contributing to increased morbidity, prolonged hospital stays, and healthcare costs. Identifying risk factors associated with POI can aid in risk stratification, preventive strategies, and enhanced patient care. This study aimed to conduct a systematic review and meta-analysis to identify and analyze the risk factors for POI following spine surgery.<br><br>Methods: A comprehensive literature search was performed using electronic databases to identify relevant studies reporting on risk factors for POI following spine surgery. The included studies were assessed for quality, and data on risk factors were extracted. Meta-analysis was conducted using random-effects models to estimate pooled odds ratios (OR) and 95% confidence intervals (CI). Heterogeneity among studies was assessed using the I^2 statistic.<br><br>Results: A total of 15 studies involving 7,236 patients were included in the systematic review. Meta-analysis revealed that advanced age (OR = 1.64, 95% CI: 1.38-1.94, p < 0.001), higher body mass index (OR = 1.27, 95% CI: 1.10-1.46, p = 0.001), longer surgical duration (OR = 1.18, 95% CI: 1.10-1.26, p < 0.001), and increased estimated blood loss (OR = 1.34, 95% CI: 1.07-1.67, p = 0.011) were significantly associated with a higher risk of developing POI following spine surgery. However, there was considerable heterogeneity among the studies (I^2 > 50%).<br><br>Conclusions: This systematic review and meta-analysis identified several risk factors associated with an increased risk of developing POI following spine surgery, including advanced age, higher body mass index, longer surgical duration, and increased estimated blood loss. These findings highlight the importance of risk stratification and targeted interventions to prevent or minimize the occurrence of POI in patients undergoing spine surgery. Further research is warranted to explore additional risk factors and develop strategies to mitigate POI in this patient population.
Bard8	Postoperative ileus (POI) is a common complication following spine surgery, affecting up to 50% of patients. POI can delay hospital discharge, increase the risk of complications, and prolong the time to return to normal activities. The aim of this study was to systematically review the literature to identify risk factors for POI following spine surgery.<br><br>A systematic search of MEDLINE, EMBASE, and Cochrane Library databases was conducted from inception to March 2023. Studies were included if they were prospective or retrospective cohort studies, case-control studies, or randomized controlled trials that assessed risk factors for POI following spine surgery. Data were extracted on study design, patient population, outcome measures, and risk factors for POI.<br><br>A total of 15 studies were included in the meta-analysis. The pooled incidence of POI was 39.3% (95% CI, 35.4-43.2%). The following risk factors for POI were identified: Older age (OR, 1.14; 95% CI, 1.04-1.25), Female gender (OR, 1.23; 95% CI, 1.07-1.41), Longer operative time (OR, 1.11; 95% CI, 1.02-1.21), Use of general anesthesia (OR, 1.26; 95% CI, 1.10-1.44), Presence of neurological deficit (OR, 1.74; 95% CI, 1.29-2.37), Use of drains (OR, 1.37; 95% CI, 1.12-1.67).<br><br>This study identified several risk factors for POI following spine surgery. These risk factors can be used to identify patients who are at increased risk for POI and to develop strategies to prevent POI in these patients.

Global Spine Journal 9

Title9	The Role of Frailty and Sarcopenia in Predicting Major Adverse Events, Length of Stay and Reoperation Following En Bloc Resection of Primary Tumours of the Spine
Abstract9	Study Design<br>Retrospective observational cohort study.<br>Objective<br>En bloc resection for primary tumours of the spine is associated with a high rate of adverse events (AEs). The objective was to explore the relationship between frailty/sarcopenia and major perioperative AEs, length of stay (LOS), and unplanned reoperation following en bloc resection of primary spinal tumours.<br>Methods<br>This is a unicentre study consisting of adult patients undergoing en bloc resection for a primary spine tumor. Frailty was calculated with the modified frailty index (mFI) and spine tumour frailty index (STFI). Sarcopenia was quantified with the total psoas area/vertebral body area ratio (TPA/VB) at L3 and L4. Univariable regression analysis was used to quantify the association between frailty/sarcopenia and major perioperative AEs, LOS and unplanned reoperation.<br>Results<br>95 patients met the inclusion criteria. The mFI and STFI identified a frailty prevalence of 3% and 18%. Mean CT TPA/VB ratios were 1.47 (SD ± .05) and 1.83 (SD ± .06) at L3 and L4. Inter-observer reliability was .93 and .99 for CT and MRI L3 and L4 TPA/VB ratios. Unadjusted analysis demonstrated sarcopenia and mFI did not predict perioperative AEs, LOS or unplanned reoperation. Frailty defined by an STFI score ≥2 predicted unplanned reoperation for surgical site infection (SSI) ($P < .05$).<br>Conclusions<br>The STFI was only associated with unplanned reoperation for SSI on unadjusted analysis, while the mFI and sarcopenia were not predictive of any outcome. Further studies are needed to investigate the relationship between frailty, sarcopenia and perioperative outcomes following en bloc resection of primary spinal tumors.
ChatGPT9	Background: En bloc resection of primary tumors of the spine is a complex surgical procedure associated with potential major adverse events (MAEs), prolonged hospital stays, and the need for reoperation. Frailty and sarcopenia have been recognized as important factors influencing surgical outcomes in various surgical specialties. However, their role in predicting outcomes following en bloc resection of primary tumors of the spine remains unclear. This study aimed to investigate the impact of frailty and sarcopenia on the occurrence of MAEs, length of stay (LOS), and reoperation in patients undergoing en bloc resection for primary spinal tumors.<br><br>Methods: A comprehensive review of the literature was conducted to identify studies evaluating frailty and sarcopenia as predictors of outcomes following en bloc resection of primary spinal tumors. Data on the occurrence of MAEs, LOS, and reoperation rates were extracted and analyzed. Meta-analysis was performed to estimate pooled odds ratios (OR) and 95% confidence intervals (CI) for the association between frailty, sarcopenia, and the outcomes of interest.<br><br>Results: A total of 10 studies comprising 879 patients were included in the analysis. Meta-analysis demonstrated that both frailty (OR = 4.10, 95% CI: 2.75-6.12, $p < 0.001$) and sarcopenia (OR = 3.25, 95% CI: 2.29-4.60, $p < 0.001$) were significantly associated with an increased risk of MAEs following en bloc resection. Frailty (OR = 2.73, 95% CI: 1.89-3.94, $p < 0.001$) and sarcopenia (OR = 2.70, 95% CI: 1.75-4.15, $p < 0.001$) were also predictors of prolonged LOS. However, there was insufficient data to perform a meta-analysis for reoperation rates.<br><br>Conclusions: Frailty and sarcopenia are important predictors of MAEs and prolonged LOS in patients undergoing en bloc resection of primary spinal tumors. These findings underscore the importance of preoperative assessment and optimization of frailty and sarcopenia to improve surgical outcomes and patient care. Further research is needed to evaluate the impact of these factors on reoperation rates and develop tailored interventions to mitigate their negative effects on outcomes.
Bard9	Background<br>En bloc resection (EBR) is a surgical procedure used to remove tumors of the spine that are confined to a single vertebral body. EBR is a complex and technically demanding procedure that can be associated with significant morbidity and mortality.<br><br>Methods<br>We conducted a retrospective study of patients who underwent EBR for primary tumors of the spine at a single institution. We evaluated the association between frailty and sarcopenia and major adverse events (MAEs), length of stay (LOS), and reoperation.<br><br>Results<br>A total of 100 patients were included in the study. The median age was 65 years (range, 20-89 years). The most common primary tumor was sarcoma (60%). The overall rate of MAEs was 20%. The median LOS was 10 days (range, 3-30 days). The rate of reoperation was 10%. Frailty and sarcopenia were independently associated with an increased risk of MAEs (odds ratio [OR], 2.0; 95% confidence interval [CI], 1.2-3.5) and LOS (OR, 1.5; 95% CI, 1.0-2.2). Frailty was also independently associated with an increased risk of reoperation (OR, 2.5; 95% CI, 1.2-5.7).<br><br>Conclusions<br>Frailty and sarcopenia are independently associated with an increased risk of MAEs, LOS, and reoperation following EBR for primary tumors of the spine. Patients with frailty or sarcopenia may benefit from a multidisciplinary approach to care that includes preoperative optimization and postoperative rehabilitation.

Global Spine Journal 10

Title10	Venous Thromboembolism Rates Have Not Decreased in Elective Lumbar Fusion Surgery from 2011 to 2020
Abstract10	Study Design<br>Retrospective cohort study.<br>Objectives<br>This study aimed to (1) evaluate for any temporal trends in the rates of VTE, deep venous thrombosis (DVT), pulmonary embolism (PE), and mortality from 2011 to 2020 and (2) identify the predictors of VTE following lumbar fusion surgery.<br>Methods<br>Annual incidences of 30-day VTE, DVT, PE, and mortality were calculated for each of the operation year groups from 2011 to 2020. Multivariable Poisson regression was utilized to test the association between operation year and primary outcomes, as well as to identify significant predictors of VTE.<br>Results<br>A total of 121,205 patients were included. There were no statistically significant differences in VTE, DVT, PE, or mortality rates among the operation year groups. Multivariable regression analysis revealed that compared to 2011, operation year 2019 was associated with significantly lower rates of DVT. Age, BMI, prolonged operation time, prolonged length of stay, non-home discharge, anterior fusion, smoking status, functional dependence, and chronic steroid use were identified as independent predictors of VTE following lumbar fusion. Female sex, Hispanic ethnicity, and outpatient surgery setting were identified as protective factors from VTE in this cohort.<br>Conclusions<br>Rates of VTE after lumbar fusion have remained mostly unchanged between 2011 and 2020. Older age, higher BMI, longer operation time, prolonged length of stay, non-home discharge, anterior fusion, smoking, functional dependence, and steroid use were independent predictors of VTE after lumbar fusion, while female sex, Hispanic ethnicity, and outpatient surgery were the protective factors.
ChatGPT10	Background: Venous thromboembolism (VTE) is a significant concern in elective lumbar fusion surgery, despite the availability of effective preventive measures. This study aimed to assess the rates of VTE and its associated outcomes in patients undergoing elective lumbar fusion surgery from 2011 to 2020.<br><br>Methods: A retrospective cohort study was conducted at a single academic medical center, including 1,000 patients who underwent elective lumbar fusion surgery. The primary outcome measure was the rate of VTE, while secondary outcomes included rates of deep vein thrombosis (DVT), pulmonary embolism (PE), and VTE-related mortality.<br><br>Results: The VTE rate was observed to be 2.0% (20 patients) in the study cohort. Specifically, the DVT rate was 1.5% (15 patients), and the PE rate was 0.5% (5 patients). Notably, no deaths resulting from VTE were recorded.<br><br>Conclusion: The findings of this study indicate that VTE rates in elective lumbar fusion surgery have remained stable from 2011 to 2020, despite the existence of effective preventive measures. These rates align with those reported in previous studies. The lack of improvement in VTE rates over the years emphasizes the need for further research to identify strategies aimed at reducing the risk of VTE in patients undergoing elective lumbar fusion surgery.
Bard10	Venous thromboembolism (VTE) is a serious complication of elective lumbar fusion surgery. Despite the availability of effective preventive measures, VTE rates have not decreased in recent years. This study examined VTE rates in patients undergoing elective lumbar fusion surgery from 2011 to 2020.<br><br>A retrospective cohort study was conducted of patients who underwent elective lumbar fusion surgery at a single academic medical center. The primary outcome was the rate of VTE. Secondary outcomes included the rate of deep vein thrombosis (DVT), the rate of pulmonary embolism (PE), and the rate of death from VTE.<br><br>A total of 1,000 patients were included in the study. The VTE rate was 2.0% (20 patients). The DVT rate was 1.5% (15 patients) and the PE rate was 0.5% (5 patients). There were no deaths from VTE.<br><br>The VTE rate in this study was similar to rates reported in other studies. The findings suggest that VTE rates have not decreased in recent years despite the availability of effective preventive measures. Additional research is needed to identify strategies to reduce the risk of VTE in patients undergoing elective lumbar fusion surgery.

Journal of Neurosurgery: Spine 1

Title1	Anterior cervical discectomy and fusion versus posterior decompression in patients with degenerative cervical myelopathy: a systematic review and meta-analysis
Abstract1	OBJECTIVE<br>The optimal surgical approach for patients with multilevel degenerative cervical myelopathy (DCM) remains unknown. This systematic review and meta-analysis sought to compare anterior cervical discectomy and fusion (ACDF) versus posterior decompression (PD) in patients with DCM spanning ≥ 2 levels without ossification of the posterior longitudinal ligament.<br><br>METHODS<br>MEDLINE and PubMed were searched from inception to February 22, 2022. The primary outcomes were Neck Disability Index (NDI), SF-36 Physical Component Summary (PCS), modified Japanese Orthopaedic Association (mJOA) scale, visual analog scale (VAS), and EQ-5D scores. Secondary outcomes were operative bleeding, operative duration, hospital length of stay (LOS), postoperative morbidity (including hematoma, surgical site infection [SSI], CSF leakage, dysphagia, dysphonia, C5 palsy, and fusion failure), mortality, readmission, reoperation, and Cobb angle.<br><br>RESULTS<br>Nineteen studies comprising 8340 patients were included, of whom 4118 (49.4%) and 4222 (50.6%) underwent ACDF and PD, respectively. The mean number of involved spinal levels was comparable between the groups (3.1 vs 3.5, p = 0.15). The mean differences (MDs) of the primary outcomes were the mean of each index in the ACDF group minus that of the PD group. At the 1-year follow-up, the MDs of the NDI (−1.67 [95% CI −3.51 to 0.18], p = 0.08), SF-36 PCS (2.48 [95% CI −0.59 to 5.55], p = 0.11), and VAS (−0.32 [95% CI −0.97 to 0.34], p = 0.35) scores were similar between the groups. While the MDs of the mJOA (0.71 [95% CI 0.27 to 1.16], p = 0.002) and EQ-5D (0.04 [95% CI 0.01 to 0.08], p = 0.02) scores were greater in the ACDF group, the differences were not clinically significant given the minimal clinically important differences (MCIDs) of 2 and 0.05 points, respectively. In the ACDF group, the MDs for operative bleeding (−102.77 ml [95% CI −169.23 to −36.30 ml], p = 0.002) and LOS (−1.42 days [95% CI −2.01 to −0.82 days], p < 0.00001) were lower, the dysphagia OR (11.10 [95% CI 5.43–22.67], p < 0.0001) was higher, and the ORs for SSI (0.43 [95% CI 0.24–0.78], p = 0.006) and C5 palsy (0.32 [95% CI 0.15–0.70], p = 0.004) were lower. The other outcomes were similar between the groups. Overall evidence according to the GRADE (Grading of Recommendations, Assessment, Development and Evaluations) approach was moderate.<br><br>CONCLUSIONS<br>ACDF and PD are similar regarding functional outcomes. ACDF is beneficial in terms of less bleeding, shorter LOS, and lower odds of SSI and C5 palsy, while the procedure carries higher odds of dysphagia. The authors recommend individualized treatment decision-making.
ChatGPT1	Objective: This systematic review and meta-analysis aimed to compare the clinical outcomes and safety of anterior cervical discectomy and fusion (ACDF) versus posterior decompression (PD) in patients diagnosed with degenerative cervical myelopathy (DCM).<br><br>Methods: A comprehensive search was conducted in major electronic databases for studies comparing ACDF and PD for the treatment of DCM. Relevant articles published up to September 2021 were considered. Data extraction and risk of bias assessment were performed independently by two reviewers. Outcome measures included clinical success rates, neurological improvement, visual analog scale (VAS) scores for neck and arm pain, Nurick scores, Odom's criteria, perioperative complications, and radiographic fusion rates.<br><br>Results: A total of 15 studies, comprising 1,826 patients, were included in the meta-analysis. The pooled analysis demonstrated comparable clinical success rates between ACDF and PD (odds ratio [OR]: 1.01, 95% confidence interval [CI]: 0.76-1.35). However, ACDF was associated with significantly higher rates of neurological improvement (OR: 1.51, 95% CI: 1.10-2.08) and better Nurick scores (mean difference: -0.31, 95% CI: -0.61 to -0.01) compared to PD. VAS scores for neck and arm pain were similar between the two groups (neck pain: mean difference: -0.06, 95% CI: -0.30 to 0.17; arm pain: mean difference: -0.01, 95% CI: -0.19 to 0.16). No significant differences were observed in Odom's criteria or perioperative complications. Radiographic fusion rates were higher in the ACDF group (OR: 4.10, 95% CI: 2.03-8.29).<br><br>Conclusion: Our systematic review and meta-analysis suggest that both ACDF and PD are effective treatment options for patients with DCM. While ACDF demonstrates superior neurological improvement and Nurick scores, it has a higher radiographic fusion rate. The choice between ACDF and PD should be based on individual patient characteristics, surgeon expertise, and shared decision-making. Further well-designed randomized controlled trials are warranted to validate these findings and provide more robust evidence to guide treatment selection in this population.
Bard1	Background: Degenerative cervical myelopathy (DCM) is a common condition that can lead to significant disability. The optimal surgical approach for DCM remains controversial.<br>Methods: We conducted a systematic review and meta-analysis to compare anterior cervical discectomy and fusion (ACDF) with posterior decompression (PD) for the treatment of DCM. We searched MEDLINE, EMBASE, and Cochrane Library from inception to February 22, 2022. The primary outcomes were Neck Disability Index (NDI), Japanese Orthopaedic Association (JOA) score, and modified Macnab criteria.<br>Results: We included 16 studies with 1,301 patients. There was no significant difference in the NDI or JOA score at 1-year follow-up between the ACDF and PD groups (NDI: -1.67 [95% CI -3.51 to 0.18], p = 0.08; JOA: 0.71 [95% CI 0.27 to 1.16], p = 0.002). The ACDF group had a lower risk of dysphagia (OR = 11.10 [95% CI 5.43-22.67], p < 0.0001) and shorter length of stay (LOS) (-1.42 days [95% CI -2.01 to -0.82 days], p < 0.00001) than the PD group. The risk of surgical site infection (SSI) and C5 palsy was similar between the groups.<br>Conclusions: ACDF and PD are similar in terms of functional outcomes at 1-year follow-up. ACDF may be associated with a lower risk of dysphagia and shorter LOS.

Journal of Neurosurgery: Spine 2

Title2	Crossing the cervicothoracic junction: an evaluation of radiographic alignment, functional outcomes, and patient-reported outcomes
Abstract2	OBJECTIVE<br>There is currently no consensus regarding the appropriate lower instrumented vertebra (LIV) for multilevel posterior cervical fusion (PCF) constructs between C7 and crossing the cervicothoracic junction (CTJ). The goal of the present study was to compare postoperative sagittal alignment and functional outcomes among adult patients presenting with cervical myelopathy undergoing multilevel PCF terminating at C7 versus spanning the CTJ.<br><br>METHODS<br>A single-institution retrospective analysis (January 2017–December 2018) was performed of patients undergoing multilevel PCF for cervical myelopathy that involved the C6–7 vertebrae. Pre- and postoperative cervical spine radiographs were analyzed for cervical lordosis, cervical sagittal vertical axis (cSVA), and first thoracic (T1) vertebral slope (T1S) in two randomized independent trials. Modified Japanese Orthopaedic Association (mJOA) and Patient-Reported Outcomes Measurement Information System (PROMIS) scores were used to compare functional and patient-reported outcomes at the 12-month postoperative follow-up.<br><br>RESULTS<br>Sixty-six consecutive patients undergoing PCF and 53 age-matched controls were included in the study. There were 36 patients in the C7 LIV cohort and 30 patients in the LIV spanning the CTJ cohort. Despite significant correction, patients undergoing fusion remained less lordotic than asymptomatic controls, with a C2–7 Cobb angle of 17.7° versus 25.5° ($p < 0.001$) and a T1S of 25.6° versus 36.3° ($p < 0.001$). The CTJ cohort had superior alignment corrections in all radiographic parameters at the 12-month postoperative follow-up compared with the C7 cohort: increase in T1S (ΔT1S 14.1° vs 2.0°, $p < 0.001$), increase in C2–7 lordosis (ΔC2–7 lordosis 11.7° vs 1.5°, $p < 0.001$), and decrease in cSVA (ΔcSVA 8.9 vs 5.0 mm, $p < 0.001$). There were no differences in the mJOA motor and sensory scores between cohorts pre- and postoperatively. The C7 cohort reported significantly better PROMIS scores at 6 months (22.0 ± 3.2 vs 11.5 ± 0.5, $p = 0.04$) and 12 months (27.0 ± 5.2 vs 13.5 ± 0.9, $p = 0.01$) postoperatively.<br><br>CONCLUSIONS<br>Crossing the CTJ may provide a greater cervical sagittal alignment correction in multilevel PCF surgeries. However, the improved alignment may not be associated with improved functional outcomes as measured by the mJOA scale. A new finding is that crossing the CTJ may be associated with worse patient-reported outcomes at 6 and 12 months of postoperative follow-up as measured by the PROMIS, which should be considered in surgical decision-making. Future prospective studies evaluating long-term radiographic, patient-reported, and functional outcomes are warranted.
ChatGPT2	Objective: This study aimed to evaluate radiographic alignment, functional outcomes, and patient-reported outcomes in patients undergoing surgical interventions involving the cervicothoracic junction (CTJ).<br><br>Methods: A retrospective analysis was conducted on patients who underwent surgical procedures involving the CTJ between January 2010 and December 2021. Radiographic measurements included cervical lordosis, thoracic kyphosis, T1 slope, CTJ angle, and C7 sagittal vertical axis (SVA). Functional outcomes were assessed using the Neck Disability Index (NDI), Short Form 36 (SF-36) physical component summary (PCS), and EuroQol-5 Dimension (EQ-5D) index. Patient-reported outcomes were obtained through visual analog scale (VAS) scores for neck and arm pain, as well as the modified Japanese Orthopedic Association (mJOA) scale. Preoperative and postoperative measurements were compared, and associations between radiographic parameters and clinical outcomes were analyzed.<br><br>Results: A total of 78 patients met the inclusion criteria. Following surgery, significant improvements were observed in cervical lordosis (mean change: 9.5°, $p < 0.001$), T1 slope (mean change: -3.2°, $p = 0.004$), and CTJ angle (mean change: -3.8°, $p = 0.006$). No significant changes were observed in thoracic kyphosis or C7 SVA. The NDI, SF-36 PCS, EQ-5D index, VAS scores for neck and arm pain, and mJOA scale all showed significant improvements postoperatively ($p < 0.001$ for all). Correlation analysis revealed a significant association between improvement in cervical lordosis and better NDI scores ($r = -0.291$, $p = 0.012$), SF-36 PCS ($r = 0.270$, $p = 0.019$), EQ-5D index ($r = 0.245$, $p = 0.032$), and mJOA scale ($r = -0.314$, $p = 0.007$).<br><br>Conclusion: Surgical interventions involving the CTJ can lead to significant improvements in radiographic alignment, functional outcomes, and patient-reported outcomes. Specifically, restoration of cervical lordosis appears to have a positive impact on neck disability, physical well-being, quality of life, and neurological function. These findings emphasize the importance of considering and optimizing CTJ alignment in surgical planning. Further prospective studies are warranted to validate these results and investigate long-term outcomes in this patient population.
Bard2	I'm unable to help, as I am only a language model and don't have the ability to process and understand that.

Journal of Neurosurgery: Spine 3

Title3	Additional growth of the spine and changes in spinal alignment after posterior spinal fusion in adolescent idiopathic scoliosis
Abstract3	OBJECTIVE<br>Previous studies have evaluated growth in spinal height immediately following surgical posterior correction of idiopathic scoliosis, yet have not reported on further spinal growth following surgery. The aims of this study were to investigate the characteristics of spinal growth after scoliosis surgery and determine whether they affect spinal alignment.<br><br>METHODS<br>The study included 91 patients (mean age 13.93 years) who underwent spinal fusion using pedicle screws for the treatment of adolescent idiopathic scoliosis (AIS). The study population included 70 female and 21 male patients. The height of the spine (HOS), length of the spine (LOS), and spinal alignment parameters were measured on anteroposterior and lateral radiographs. A stepwise multiple linear regression analysis was used to examine the variables that affected HOS gain from growth. The patients were divided into two groups, the growth group and the nongrowth group, according to whether the HOS gain from growth exceeded 1 cm, to analyze the effect of spinal growth on its alignment.<br><br>RESULTS<br>The mean (± SD) HOS gain from growth was 0.88 ± 0.66 (range −0.46 to 3.21) cm, with 40.66% of patients exhibiting growth ≥ 1 cm. This increase was significantly related to young age, male sex, and a small Risser stage (sex: b = −0.532, p < 0.001, male = 1, female = 2; Risser stage: b = −0.185, p < 0.001; age: b = −0.125, p = 0.011; adjusted R2 = 0.442). The variation in LOS was similar to that of HOS. Thoracic kyphosis and upper instrumented vertebra–lowest instrumented vertebra Cobb angle were reduced in both groups, with a greater reduction observed in the growth group. Patients with an increase in HOS < 1 cm showed a larger lumbar lordosis and a greater tendency for the sagittal vertical axis (SVA) to shift backward and the pelvic tilt to decrease (anteverted pelvis) than in the growth group.<br><br>CONCLUSIONS<br>The spine still has growth potential after corrective fusion surgery for AIS, and 40.66% of the patients in this study continued to vertically grow by 1 cm or more. Unfortunately, the height changes cannot be accurately predicted by currently measured parameters. Changes in the sagittal alignment of the spine may affect the vertical growth increment.
ChatGPT3	Objective: This study aims to evaluate the additional growth of the spine and changes in spinal alignment following posterior spinal fusion (PSF) in patients with adolescent idiopathic scoliosis (AIS).<br><br>Methods: A retrospective analysis was conducted on a cohort of 150 patients with AIS who underwent PSF. Radiographic measurements were obtained preoperatively, postoperatively, and at final follow-up to assess changes in spinal alignment parameters, including Cobb angle, sagittal vertical axis (SVA), and pelvic parameters. The amount of additional growth of the spine after PSF was calculated by comparing the final follow-up height with the height at the time of surgery. Subgroup analyses were performed based on the preoperative Risser stage and surgical instrumentation technique.<br><br>Results: Among the 150 patients included in the study, the mean follow-up duration was 5 years. The average additional growth of the spine after PSF was 3.2 cm (6.7%) during the follow-up period. Subgroup analysis based on preoperative Risser stage revealed that patients with higher Risser scores (Risser 4 or 5) had significantly less additional growth compared to those with lower Risser scores (Risser 0 to 3) (p < 0.001). Regarding surgical instrumentation technique, patients who underwent pedicle screw fixation showed significantly greater additional growth compared to those treated with hybrid instrumentation (p = 0.02). Postoperatively, significant improvements were observed in the Cobb angle (p < 0.001) and SVA (p < 0.001), indicating improved spinal alignment.<br><br>Conclusion: Posterior spinal fusion in patients with AIS allows for additional growth of the spine during the follow-up period. However, patients with higher Risser scores tend to have less additional growth. Pedicle screw fixation appears to facilitate greater growth compared to hybrid instrumentation. These findings provide valuable insights into the changes in spinal alignment and growth potential following PSF in AIS patients, aiding in surgical planning and patient counseling.
Bard3	Background: Adolescent idiopathic scoliosis (AIS) is a common spinal deformity that can cause significant pain and disability. Posterior spinal fusion (PSF) is a surgical treatment that can correct the deformity and prevent further progression. However, PSF can also restrict spinal growth, which can lead to complications such as decreased height and spinal imbalance.<br><br>Methods: We conducted a retrospective study of 91 patients with AIS who underwent PSF using pedicle screws. The patients were followed for an average of 5 years. We measured the height of the spine, length of the spine, and spinal alignment parameters on anteroposterior and lateral radiographs. We also used a stepwise multiple linear regression analysis to examine the variables that affected growth of the spine.<br><br>Results: The mean (± SD) height of the spine increased by 0.88 ± 0.66 cm (range, −0.46 to 3.21 cm) after PSF. The mean (± SD) length of the spine increased by 0.94 ± 0.71 cm (range, −0.31 to 3.73 cm). Thoracic kyphosis and upper instrumented vertebra-lowest instrumented vertebra Cobb angle decreased in both groups, with a greater reduction observed in the growth group. Patients with an increase in height of the spine < 1 cm showed a larger lumbar lordosis and a greater tendency for the sagittal vertical axis (SVA) to shift backward and the pelvic tilt to decrease (anteverted pelvis) than in the growth group.<br><br>Conclusions: Our findings suggest that the spine still has growth potential after PSF for AIS. However, the amount of growth is variable and may not be sufficient to prevent all complications associated with restricted spinal growth. Patients with a small increase in height of the spine after PSF may be at increased risk for developing spinal imbalance.

Journal of Neurosurgery: Spine 4

Title4	Factors associated with readmission after minimally invasive transforaminal lumbar interbody fusion
Abstract4	OBJECTIVE<br>The objective of this study was to identify factors that lead to a prolonged hospital stay or 30-day readmission after minimally invasive surgery (MIS) for transforaminal lumbar interbody fusion (TLIF) at a single institution.<br><br>METHODS<br>Consecutive patients who underwent MIS TLIF from January 1, 2016, to March 31, 2018, were retrospectively analyzed. Demographic data, including age, sex, ethnicity, smoking status, and body mass index, were collected along with operative details, indications, affected spinal levels, estimated blood loss, and operative duration. The effects of these data were evaluated relative to the hospital length of stay (LOS) and 30-day readmission.<br><br>RESULTS<br>The authors identified 174 consecutive patients who underwent MIS TLIF at 1 or 2 levels from a prospectively collected database. The mean (range) patient age was 64.1 (31–81) years, 97 were women (56%), and 77 were men (44%). Of 182 levels fused, 127 were done at L4–5 (70%), 32 at L3–4 (18%), 13 at L5–S1 (7%), and 10 at L2–3 (5%). Patients underwent 166 (95%) single-level procedures and 8 (5%) 2-level procedures. The mean (range) procedural duration, defined as the time from incision to closure, was 164.6 (90–529) minutes. The mean (range) LOS was 1.8 (0–8) days. Eleven patients (6%) were readmitted within 30 days; the most frequent causes were urinary retention, constipation, and persistent or contralateral symptoms. Seventeen patients had LOS greater than 3 days. Six of those patients (35%) were identified as widows, widowers, or divorced, and 5 of them lived alone. Six patients with prolonged LOS (35%) required placement in either skilled nursing or acute inpatient rehabilitation. Regression analyses showed living alone (p = 0.04) and diabetes (p = 0.04) as predictors of readmission. Regression analyses revealed female sex (p = 0.03), diabetes (p = 0.03), and multilevel surgery (p = 0.006) as predictors of LOS > 3 days.<br><br>CONCLUSIONS<br>Urinary retention, constipation, and persistent radicular symptoms were the leading causes of readmission within 30 days of surgery in this series, which is distinct from data from the American College of Surgeons National Surgical Quality Improvement Program. The inability to discharge a patient home for social reasons led to prolonged inpatient hospital stays. Identifying these risk factors and proactively addressing them could lower readmission rates and decrease LOS among patients undergoing MIS TLIF.
ChatGPT4	Objective: This study aims to identify the factors associated with readmission following minimally invasive transforaminal lumbar interbody fusion (MIS TLIF) and analyze their impact on patient outcomes.<br><br>Methods: A retrospective analysis was conducted on a cohort of 250 patients who underwent MIS TLIF for degenerative lumbar spine conditions. Electronic medical records were reviewed to identify patient demographics, comorbidities, surgical variables, and postoperative complications. The primary outcome measure was unplanned readmission within 30 days of the initial surgery. Univariate and multivariate analyses were performed to identify factors associated with readmission and assess their significance.<br><br>Results: Among the 250 patients included in the study, 30 patients (12%) required readmission within 30 days of the initial surgery. The most common reasons for readmission were surgical site infection (n = 15, 6%), followed by postoperative pain management issues (n = 10, 4%). Univariate analysis revealed that older age, higher body mass index (BMI), presence of diabetes mellitus, longer operative time, and perioperative complications were significantly associated with increased readmission rates (p < 0.05). Multivariate analysis identified older age (odds ratio [OR] = 2.5, 95% confidence interval [CI]: 1.2-5.3, p = 0.015), higher BMI (OR = 1.8, 95% CI: 1.1-3.1, p = 0.025), and surgical site infection (OR = 3.4, 95% CI: 1.7-6.8, p = 0.001) as independent predictors of readmission.<br><br>Conclusion: Unplanned readmission following MIS TLIF is associated with older age, higher BMI, and surgical site infection. These factors should be taken into consideration during preoperative planning and patient counseling. Efforts to optimize patient health and minimize perioperative complications, particularly related to infection control, may help reduce the risk of readmission and improve patient outcomes. Further studies are warranted to validate these findings and explore strategies for preventing readmissions in this patient population.
Bard4	Background: Minimally invasive transforaminal lumbar interbody fusion (MIS-TLIF) is a surgical procedure used to treat lumbar spinal stenosis and disc herniation. Readmission to the hospital within 30 days of surgery is a common complication, and the factors associated with readmission are not well understood.<br>Methods: We conducted a retrospective study of patients who underwent MIS-TLIF at a single institution. We identified patients who were readmitted to the hospital within 30 days of surgery and compared them to patients who were not readmitted. We evaluated demographic data, medical history, surgical details, and postoperative course.<br>Results: Of 174 patients who underwent MIS-TLIF, 11 (6%) were readmitted to the hospital within 30 days of surgery. The most common reasons for readmission were urinary retention (36%), constipation (27%), and persistent radicular symptoms (27%). Patients who were readmitted were more likely to be female (73%), have diabetes (45%), and have multilevel surgery (73%).<br>Conclusions: Our study findings suggest that female sex, diabetes, and multilevel surgery are associated with an increased risk of readmission after MIS-TLIF. These findings should be considered when counseling patients about the risks and benefits of MIS-TLIF.

Journal of Neurosurgery: Spine 5

Title5	Examining degenerative disease adjacent to lumbosacral transitional vertebrae: a retrospective cohort study
Abstract5	OBJECTIVE<br>Bertolotti syndrome is a clinical diagnosis given to patients with low-back pain arising from a lumbosacral transitional vertebra (LSTV). While biomechanical studies have demonstrated abnormal torques and range of motion occurring at and above this type of LSTV, the long-term effects of these biomechanical changes on the LSTV adjacent segments are not well understood. This study examined degenerative changes at segments superjacent to the LSTV in patients with Bertolotti syndrome.<br><br>METHODS<br>This study involved a retrospective comparison of patients between 2010 and 2020 with an LSTV and chronic back pain (Bertolotti syndrome) and control patients with chronic back pain with no LSTV. The presence of an LSTV was confirmed on imaging, and the caudal-most mobile segment above the LSTV was assessed for degenerative changes. Degenerative changes were assessed by grading the intervertebral disc, facets, degree of spinal stenosis, and spondylolisthesis using well documented grading systems. All computations were performed in R, version 4.1.0. All tests were two-sided, and p values < 0.05 was considered statistically significant. Separate logistic regression analyses were run with the associated dependent variables for each aim, with age at MRI and sex included as covariates. Odds ratios and 95% confidence intervals were computed.<br><br>RESULTS<br>A total of 172 patients were included, 101 with Bertolotti syndrome and 71 controls. Control patients consisted of patients with low-back pain but no diagnosis of Bertolotti syndrome or an LSTV. Fifty-six Bertolotti (55.4%) and 27 control (38.0%) patients were female, (p = 0.03). After adjusting for age at MRI and sex, Bertolotti patients had pelvic incidence (PI) that was 9.83° greater than control patients (95% CI 5.15°–14.50°, p < 0.001). Sacral slope was not significantly different between the Bertolotti and control groups (beta estimate 3.10°, 95% CI −1.07° to 7.27°; p = 0.14). Bertolotti patients had 2.69 times higher odds of having a high disc grade at L4–5 (3–4 vs 0–2), compared with control patients (OR 2.69, 95% CI 1.28–5.90; p = 0.01). There were no significant differences between Bertolotti patients and controls for spondylolisthesis, facet grade, or spinal stenosis grade.<br><br>CONCLUSIONS<br>Patients with Bertolotti syndrome had a significantly higher PI and were more likely to have adjacent-segment disease (ASD; L4–5) compared with control patients. However, after controlling for age and sex, PI and ASD did not appear to have a significant association within the cohort of Bertolotti patients. The altered biomechanics and kinematics in this condition may be a causative factor in this degeneration, although proof of causation is not possible in this study. This association may warrant closer follow-up protocols for patients being treated for Bertolotti syndrome, but further prospective studies are needed to establish if radiographic parameters can serve as an indicator for biomechanical alterations in vivo.
ChatGPT5	Objective: This retrospective cohort study aims to investigate the prevalence and characteristics of degenerative disease in the segments adjacent to lumbosacral transitional vertebrae (LSTV) and evaluate their impact on clinical outcomes in patients with lumbar spine pathology.<br><br>Methods: A comprehensive analysis was conducted on a retrospective cohort of 200 patients with lumbar spine pathology who underwent surgical intervention. LSTV was identified and classified according to the Castellvi classification system. Radiographic evaluations, including magnetic resonance imaging (MRI) and computed tomography (CT) scans, were performed to assess the presence and extent of degenerative changes in the adjacent segments. Clinical outcomes were assessed using standardized outcome measures. The prevalence and specific characteristics of degenerative disease, including disc degeneration, facet joint osteoarthritis, and foraminal stenosis, were recorded. The association between LSTV classification and the presence of adjacent degenerative disease was analyzed, and its impact on clinical outcomes was evaluated.<br><br>Results: Among the 200 patients included in the study, 90 patients (45%) had LSTV, with the most common subtype being Castellvi type II (n = 40). Degenerative disease in the segments adjacent to LSTV was observed in 75 patients (37.5%), with disc degeneration being the most prevalent (n = 60, 30%). Facet joint osteoarthritis and foraminal stenosis were present in 45 patients (22.5%) and 35 patients (17.5%), respectively. The presence of degenerative disease in adjacent segments was significantly associated with the LSTV subtype (p < 0.001). However, no significant association was found between the presence of degenerative disease and clinical outcomes.<br><br>Conclusion: Degenerative disease in the segments adjacent to LSTV is common in patients with lumbar spine pathology, with disc degeneration being the most prevalent. The classification of LSTV is associated with the presence of adjacent degenerative changes. However, the presence of degenerative disease in adjacent segments does not appear to have a significant impact on clinical outcomes in this cohort. These findings emphasize the importance of evaluating the presence of LSTV and associated degenerative changes when planning surgical interventions in patients with lumbar spine pathology.
Bard5	Background: Lumbosacral transitional vertebrae (LSTVs) are common variants that occur when the fifth lumbar vertebra has a sacrum-like morphology. LSTVs are associated with an increased risk of degenerative disease, including disc herniation, spondylolisthesis, and spinal stenosis.<br>Methods: We conducted a retrospective cohort study of patients with LSTVs who underwent lumbar spine surgery. We compared the prevalence of degenerative disease at adjacent segments in patients with LSTVs to a control group of patients without LSTVs.<br>Results: We identified 100 patients with LSTVs and 50 control patients without LSTVs. The prevalence of degenerative disease at adjacent segments was significantly higher in patients with LSTVs than in control patients (58% vs. 28%; p < 0.001). The most common type of degenerative disease was disc herniation (38%), followed by spondylolisthesis (22%) and spinal stenosis (18%).<br>Conclusions: Our study findings suggest that LSTVs are associated with an increased risk of degenerative disease at adjacent segments. This increased risk should be considered when managing patients with LSTVs.

Journal of Neurosurgery: Spine 6

Title6	Vertebral bone quality score provides preoperative bone density assessment for patients undergoing lumbar spine surgery: a retrospective study
Abstract6	OBJECTIVE<br>The novel MRI-based vertebral bone quality (VBQ) score has been described as an opportunistic screening tool for osteoporosis, but the stability and practical value of this score deserve further investigation. The purpose of this study was to assess whether preoperative VBQ scores could assist in identifying reduced bone mineral density (BMD) or osteoporosis and evaluating the consistency between MRI systems with different field strengths.<br><br>METHODS<br>The VBQ scores of the patients who underwent surgery for lumbar disc herniation and the single-level VBQ scores of each L1–4 vertebral body were measured and calculated with preoperative lumbar MRI noncontrast T1-weighted phases. The VBQ scores were evaluated for correlation analysis using dual-energy x-ray absorptiometry (DEXA) T-scores. The receiver operating characteristic (ROC) curve was used to evaluate the ability of the VBQ scores to identify patients with reduced BMD and with osteoporosis. Differences in CSF measurements at different levels of L1–4 were compared. Twenty-four patients who had been examined using another MRI machine were used as controls to test the interdevice agreement of the VBQ scores.<br><br>RESULTS<br>The study included 100 patients with mean VBQ scores of 2.81 ± 0.28 (normal BMD), 3.06 ± 0.36 (osteopenia), and 3.43 ± 0.37 (osteoporosis). VBQ scores differed significantly between BMD subgroups ($p < 0.001$). The Pearson correlation coefficient showed a moderate negative linear correlation between novel VBQ scores and the lowest DEXA T-scores ($r = −0.524$). ROC analysis showed good discrimination of VBQ scores in patients with reduced BMD (area under the curve [AUC] 0.793) and with osteoporosis (AUC 0.810). The diagnostic thresholds of reduced BMD and osteoporosis according to the maximum Youden index were 3.06 (sensitivity 0.636, specificity 0.870, positive predictive value [PPV] 0.942, negative predictive value [NPV] 0.417) and 3.05 (sensitivity 0.875, specificity 0.618, PPV 0.519, NPV 0.913), respectively. CSF measurements at the L2, L3, and L4 levels were essentially identical and did not significantly affect the final VBQ scores ($p > 0.05$), whereas CSF measurements at the L1 level were found to be heterogeneous ($p < 0.001$). No significant differences were found in VBQ scores between the same brand of MRI machines at different field strengths (1.5 and 3.0 T, $p = 0.107$).<br><br>CONCLUSIONS<br>The new VBQ score provides an additional screening opportunity for preoperative BMD assessment. A VBQ score < 3.05 essentially excludes osteoporosis, whereas a VBQ score ≥ 3.05 (especially ≥ 3.06) suggests the need for further examination. The VBQ score is comparable between different MRI systems.
ChatGPT6	Objective: This retrospective study aims to assess the utility of the vertebral bone quality score (VBQS) as a preoperative bone density assessment tool for patients undergoing lumbar spine surgery.<br><br>Methods: A retrospective analysis was conducted on a cohort of 150 patients who underwent lumbar spine surgery. Preoperative dual-energy X-ray absorptiometry (DXA) scans were obtained to measure bone mineral density (BMD) at the lumbar spine and hip regions. The VBQS, a novel scoring system based on the evaluation of vertebral trabecular bone quality using preoperative computed tomography (CT) scans, was calculated for each patient. The primary outcome measures included the correlation between VBQS and DXA-derived BMD, as well as the predictive value of VBQS in identifying patients at risk for postoperative complications related to bone quality.<br><br>Results: The study included 150 patients undergoing lumbar spine surgery. The mean VBQS was 4.7, ranging from 2.1 to 8.9. A significant positive correlation was observed between VBQS and DXA-derived BMD at the lumbar spine ($r = 0.67$, $p < 0.001$) and hip regions ($r = 0.54$, $p < 0.001$). Patients with a lower VBQS had a higher incidence of postoperative complications related to poor bone quality, including instrumentation failure and vertebral fractures (72 patients, 48%).<br><br>Conclusion: The VBQS provides a reliable preoperative assessment of bone density in patients undergoing lumbar spine surgery. The VBQS demonstrates a strong correlation with DXA-derived BMD measurements, indicating its potential as a surrogate marker for bone quality evaluation. Furthermore, the VBQS can aid in identifying patients at higher risk (48% of the cohort) for postoperative complications related to poor bone quality, facilitating appropriate surgical planning and implant selection. Incorporating the VBQS into preoperative evaluations may contribute to improved patient outcomes and reduce the incidence of complications associated with compromised bone quality.
Bard6	Background: Osteoporosis is a major risk factor for complications after lumbar spine surgery. The vertebral bone quality (VBQ) score is a novel MRI-based measure of vertebral bone strength. We investigated the ability of VBQ scores to predict preoperative bone mineral density (BMD) and to identify patients with osteoporosis in patients undergoing lumbar spine surgery.<br>Methods: We retrospectively reviewed the medical records of 100 patients who underwent lumbar spine surgery. VBQ scores were calculated for each L1-4 vertebral body on preoperative lumbar MRI noncontrast T1-weighted images. BMD was measured using dual-energy x-ray absorptiometry (DEXA). Patients were classified as having normal BMD, osteopenia, or osteoporosis based on DEXA T-scores.<br>Results: The mean VBQ score was significantly higher in patients with osteopenia (3.06 ± 0.36) or osteoporosis (3.43 ± 0.37) than in patients with normal BMD (2.81 ± 0.28; $p < 0.001$). There was a moderate negative correlation between VBQ scores and DEXA T-scores ($r = −0.524$; $p < 0.001$). VBQ scores had good discrimination for both reduced BMD (area under the curve [AUC] = 0.793) and osteoporosis (AUC = 0.810). The diagnostic thresholds for reduced BMD and osteoporosis according to the maximum Youden index were 3.06 (sensitivity 0.636, specificity 0.870) and 3.05 (sensitivity 0.875, specificity 0.618), respectively.<br>Conclusions: VBQ scores are a novel MRI-based measure of vertebral bone strength that can be used to predict preoperative BMD and to identify patients with osteoporosis in patients undergoing lumbar spine surgery. VBQ scores may be a useful tool for preoperative risk stratification and for planning surgical interventions.

Journal of Neurosurgery: Spine 7

Title7	Evaluation of lymphopenia as a predictor of postoperative mortality and major complications in patients undergoing surgery for metastatic spine tumors
Abstract7	OBJECTIVE<br>Lymphopenia is often seen in advanced metastatic disease and has been associated with poor postoperative outcomes. Limited research has been done to validate this metric in patients with spinal metastases. The objective of this study was to evaluate the capability of preoperative lymphopenia to predict 30-day mortality, overall survival (OS), and major complications in patients undergoing surgery for metastatic spine tumors.<br><br>METHODS<br>A total of 153 patients who underwent surgery for metastatic spine tumor between 2012 and 2022 and met the inclusion criteria were examined. Electronic medical record chart review was conducted to obtain patient demographics, comorbidities, preoperative laboratory values, survival time, and postoperative complications. Preoperative lymphopenia was defined as < 1.0 K/μL based on the institution's laboratory cutoff value and within 30 days prior to surgery. The primary outcome was 30-day mortality. Secondary outcomes were OS up to 2 years and 30-day postoperative major complications. Outcomes were assessed with logistic regression. Survival analyses were done using the Kaplan-Meier method with log-rank test and Cox regression. Receiver operating characteristic curves were plotted to classify the predictive ability of lymphocyte count as a continuous variable on outcome measures.<br><br>RESULTS<br>Lymphopenia was identified in 47% of patients (72 of 153). The overall 30-day mortality rate was 9% (13 of 153). In logistic regression analysis, lymphopenia was not associated with 30-day mortality (OR 1.35, 95% CI 0.43–4.21; p = 0.609). The mean OS in this sample was 15.6 months (95% CI 13.9–17.3 months), with no significant difference between patients with lymphopenia and those with no lymphopenia (p = 0.157). Cox regression analysis did not show an association between lymphopenia and survival (HR 1.44, 95% CI 0.87–2.39; p = 0.161). The major complication rate was 26% (39 of 153). In univariable logistic regression analysis, lymphopenia was not associated with the development of a major complication (OR 1.44, 95% CI 0.70–3.00; p = 0.326). Finally, receiver operating characteristic curves generated poor discrimination between lymphocyte count and all outcomes, including 30-day mortality (area under the curve 0.600, p = 0.232).<br><br>CONCLUSIONS<br>This study does not support prior research that had shown an independent association between low preoperative lymphocyte level and poor postoperative outcomes following surgery for metastatic spine tumors. Although lymphopenia may be used to predict outcomes in other tumor-related surgeries, this metric may not hold a similar predictive capability in the population undergoing surgery for metastatic spine tumors. Further research into reliable prognostic tools is needed.
ChatGPT7	Objective: This retrospective study aims to investigate the association between preoperative lymphopenia and postoperative mortality, as well as major complications, in patients undergoing surgery for metastatic spine tumors.<br><br>Methods: A comprehensive analysis was conducted on a retrospective cohort of 250 patients who underwent surgical intervention for metastatic spine tumors. Preoperative complete blood counts were retrieved, and lymphopenia was defined as an absolute lymphocyte count below the threshold of 1,000 cells/μL. The primary outcomes of interest were postoperative mortality within 30 days and the occurrence of major complications. Multivariable logistic regression models were employed to assess the independent association between lymphopenia and the outcomes, adjusting for relevant confounding variables.<br><br>Results: Among the 250 patients included in the study, 75 patients (30%) exhibited preoperative lymphopenia. The postoperative mortality rate within 30 days was 22 cases (8.8%). Lymphopenia was significantly associated with increased postoperative mortality (odds ratio [OR] [95% confidence interval (CI)]: 3.42 [1.87 to 6.25], p<0.001) and major complications (OR [95% CI]: 2.95 [1.69 to 5.14], p<0.001) after adjusting for confounding factors. Subgroup analyses revealed consistent findings across different tumor types and patient characteristics.<br><br>Conclusion: Preoperative lymphopenia serves as a valuable predictor of postoperative mortality and major complications in patients undergoing surgery for metastatic spine tumors. The presence of lymphopenia, observed in 30% of patients, indicates an impaired immune status, which may compromise the patient's ability to withstand the physiological stress associated with surgery and increase susceptibility to complications. Preoperative assessment of lymphocyte counts can aid in risk stratification and guide surgical decision-making, warranting close postoperative monitoring and potential interventions to optimize outcomes.
Bard7	Background: Metastatic spine tumors are a common cause of morbidity and mortality. Surgery is often the treatment of choice for these tumors, but it is associated with a high risk of complications. The purpose of this study was to evaluate the association of lymphopenia with postoperative mortality and major complications in patients undergoing surgery for metastatic spine tumors.<br>Methods: We conducted a retrospective review of patients who underwent surgery for metastatic spine tumors at our institution from 2015 to 2022. We identified 100 patients, of whom 25 (25%) had lymphopenia (defined as a lymphocyte count < 1,500 cells/μL). We analyzed the clinical characteristics, postoperative outcomes, and complications of these patients.<br>Results: The median age of the patients was 65 years (range, 20-95 years). The most common primary tumors were lung cancer (50%), breast cancer (25%), and prostate cancer (25%). The median duration of follow-up was 12 months (range, 1-60 months). The overall mortality rate was 20%. The most common major complications were deep vein thrombosis (15%), pneumonia (10%), and surgical site infection (10%). Patients with lymphopenia had a significantly higher risk of postoperative mortality (p = 0.005) and major complications (p = 0.01).<br>Conclusions: Lymphopenia is a significant predictor of postoperative mortality and major complications in patients undergoing surgery for metastatic spine tumors. Patients with lymphopenia should be closely monitored and may benefit from additional interventions to reduce the risk of complications.

Journal of Neurosurgery: Spine 8

Title8	Beta-lactam–resistant Staphylococcus aureus in spinal osteomyelitis and spondylodiscitis: current landscape in antibiotic resistance, treatment, and complications
Abstract8	OBJECTIVE<br>Spinal osteomyelitis and spondylodiscitis (SD) are infections of the vertebral body and disc, respectively, with more than 50% associated with Staphylococcus aureus. Methicillin-resistant S. aureus (MRSA) has become a pathogen of interest in cases of SD due to increasing prevalence. The purpose of this investigation was to characterize the current epidemiological and microbiological landscape in SD cases, in addition to medical and surgical challenges in treating these infections.<br><br>METHODS<br>The PearlDiver Mariner database was queried for ICD-10 codes to identify cases of SD from 2015 to 2021. The initial cohort was stratified by offending pathogens, including methicillin-sensitive S. aureus (MSSA) and MRSA. Primary outcome measures included epidemiological trends, demographics, and rates of surgical management. Secondary outcomes included length of hospital stay, rate of reoperation, and complications associated with surgical cases. Multivariable logistic regression was used to control for age, gender, region, and Charlson Comorbidity Index (CCI).<br><br>RESULTS<br>Nine thousand nine hundred eighty-three patients met the inclusion criteria and were retained for this study. Approximately half (45.5%) of SD cases resulting from S. aureus infections each year were resistant to beta-lactam antibiotics. The proportion of cases that were managed surgically was 31.02%. Among the cases that involved surgical intervention, 21.83% underwent revision operations within 30 days of the original procedure and 37.29% of cases returned to the operating room within 1 year. Substance abuse, specifically alcohol, tobacco, and drug use, were strong predictors for surgical intervention in cases of SD (all $p < 0.001$), in addition to obesity ($p = 0.002$), liver disease ($p < 0.001$), and valvular disease ($p = 0.025$). After adjusting for age, gender, region, and CCI, cases of MRSA were more likely to undergo surgical management (OR 1.19, $p = 0.003$). MRSA SD also exhibited higher rates of reoperation within 6 months (OR 1.29, $p = 0.001$) and 1 year (OR 1.36, $p < 0.001$). Surgical cases resulting from MRSA infections also exhibited higher morbidity and significant rates of transfusion (OR 1.47, $p = 0.030$), acute kidney injury (OR 1.35, $p = 0.001$), pulmonary embolism (OR 1.44, $p = 0.030$), pneumonia (OR 1.49, $p = 0.002$), and urinary tract infection (OR 1.45, $p = 0.002$) compared with MSSA SD infections.<br><br>CONCLUSIONS<br>More than 45% of cases of S. aureus SD in the US are resistant to beta-lactam antibiotics, presenting obstacles in treatment. Cases of MRSA SD are more likely to be managed surgically and have higher rates of complications and reoperations. Early detection and prompt operative management are imperative to reduce the risk of complications.
ChatGPT8	Objective: This comprehensive review aims to provide an updated overview of beta-lactam–resistant Staphylococcus aureus (BLRSA) in spinal osteomyelitis and spondylodiscitis, focusing on the current landscape of antibiotic resistance, treatment strategies, and associated complications.<br><br>Methods: A systematic literature search was conducted using major scientific databases to identify relevant studies published from 2015 to 2021. Articles addressing BLRSA in spinal osteomyelitis and spondylodiscitis were included for analysis. Data on antibiotic resistance patterns, treatment modalities, outcomes, and complications were extracted and synthesized.<br><br>Results: A total of 75 studies met the inclusion criteria and were included in the analysis. BLRSA was found to be present in 35% of cases of spinal osteomyelitis and spondylodiscitis. Methicillin-resistant Staphylococcus aureus (MRSA) accounted for the majority of BLRSA infections, with a prevalence of 80% among the BLRSA cases. Regarding treatment strategies, alternative antimicrobial agents, such as vancomycin, daptomycin, and linezolid, were utilized in 65% of cases due to the resistance of BLRSA strains to beta-lactam antibiotics. Surgical intervention was performed in 90% of cases to achieve adequate debridement and enhance antibiotic penetration. Complications associated with BLRSA-related spinal infections were observed in 50% of cases, including persistent infection, neurological deficits, hardware failure, and recurrent infections.<br><br>Conclusion: BLRSA represents a significant challenge in the management of spinal osteomyelitis and spondylodiscitis, with a prevalence of 35% among cases. MRSA is the predominant BLRSA strain encountered in these infections. Alternative antimicrobial agents and surgical intervention play crucial roles in the treatment of BLRSA-related spinal infections. Complications associated with BLRSA infections are prevalent and warrant attention. Strategies such as antimicrobial stewardship programs, surveillance systems, and research on novel therapeutic agents are essential to address the increasing burden of BLRSA and mitigate associated complications.
Bard8	Background: Staphylococcus aureus (S. aureus) is a common pathogen in spinal osteomyelitis and spondylodiscitis. In recent years, there has been an increase in the prevalence of beta-lactam–resistant S. aureus (MRSA) strains. This has made the treatment of spinal infections more challenging.<br>Methods: We conducted a retrospective review of patients with spinal osteomyelitis and spondylodiscitis who were treated at our institution from 2015 to 2022. We identified 100 patients, of whom 45 (45%) had MRSA infections. We analyzed the clinical characteristics, treatment outcomes, and complications of these patients.<br>Results: The median age of the patients was 65 years (range, 20-95 years). The most common symptoms were back pain (95%) and fever (85%). The majority of patients (75%) had underlying medical conditions, such as diabetes mellitus (25%), chronic kidney disease (20%), and rheumatoid arthritis (15%). The median duration of antibiotic therapy was 6 weeks (range, 4-12 weeks). The overall success rate of treatment was 85%. The most common complications were surgical site infection (15%) and deep vein thrombosis (10%).<br>Conclusions: MRSA is a growing problem in the treatment of spinal osteomyelitis and spondylodiscitis. Patients with MRSA infections are more likely to have underlying medical conditions and to experience complications. Despite these challenges, the overall success rate of treatment is high.

Journal of Neurosurgery: Spine 9

Title9	Hypotension requiring vasopressor treatment and increased cardiac complications in elderly spinal cord injury patients: a prospective TRACK-SCI registry study
Abstract9	OBJECTIVE<br>Increasing life expectancy has led to an older population. In this study, the authors analyzed complications and outcomes in elderly patients following spinal cord injury (SCI) using the established multi-institutional prospective study Transforming Research and Clinical Knowledge in SCI (TRACK-SCI) database collected in the Department of Neurosurgical Surgery at the University of California, San Francisco.<br><br>METHODS<br>TRACK-SCI was queried for elderly individuals (≥ 65 years of age) with traumatic SCI from 2015 to 2019. Primary outcomes of interest included total hospital length of stay, perioperative complications, postoperative complications, and in-hospital mortality. Secondary outcomes included disposition location, and neurological improvement based on the American Spinal Injury Association Impairment Scale (AIS) grade at discharge. Descriptive analysis, Fisher's exact test, univariate analysis, and multivariable regression analysis were performed.<br><br>RESULTS<br>The study cohort consisted of 40 elderly patients. The in-hospital mortality rate was 10%. Every patient in this cohort experienced at least 1 complication, with a mean of 6.6 separate complications (median 6, mode 4). The most common complication categories were cardiovascular, with a mean of 1.6 complications (median 1, mode 1), and pulmonary, with a mean of 1.3 (median 1, mode 0) complications, with 35 patients (87.5%) having at least 1 cardiovascular complication and 25 (62.5%) having at least 1 pulmonary complication. Overall, 32 patients (80%) required vasopressor treatment for mean arterial pressure (MAP) maintenance goals. The use of norepinephrine correlated with increased cardiovascular complications. Only 3 patients (7.5%) of the total cohort had an improved AIS grade compared with their acute level at admission.<br><br>CONCLUSIONS<br>Given the increased frequency of cardiovascular complications associated with vasopressor use in elderly SCI patients, caution is warranted when targeting MAP goals in these patients. A downward adjustment of blood pressure maintenance goals and prophylactic cardiology consultation to select the most appropriate vasopressor agent may be advisable for SCI patients ≥ 65 years of age.
ChatGPT9	Objective: This prospective study aimed to evaluate the association between hypotension requiring vasopressor treatment and cardiac complications in elderly patients with spinal cord injury (SCI) using data from the TRACK-SCI registry.<br><br>Methods: The TRACK-SCI registry provided a cohort of 250 elderly patients (age ≥ 65 years) with traumatic SCI. Baseline demographic, injury-related, and clinical characteristics were recorded. The primary exposure of interest was hypotension requiring vasopressor treatment, defined as the need for vasopressor administration to maintain mean arterial pressure above a specified threshold. The primary outcome measure was the occurrence of cardiac complications, including myocardial infarction, arrhythmias, and cardiac arrest. Multivariable logistic regression models were utilized to assess the association between hypotension requiring vasopressor treatment and cardiac complications, adjusting for relevant confounders.<br><br>Results: A total of 185 elderly patients with SCI (74%) required vasopressor treatment for hypotension. Among these patients, 62 (33.5%) experienced cardiac complications. After adjusting for potential confounders, hypotension requiring vasopressor treatment was significantly associated with an increased risk of cardiac complications (odds ratio [OR] [95% confidence interval (CI)]: 2.57 [1.62 to 4.08], p<0.001). Subgroup analyses demonstrated consistent findings across different injury severities and neurological levels.<br><br>Conclusion: In elderly patients with spinal cord injury, hypotension requiring vasopressor treatment is independently associated with an elevated risk of cardiac complications. These findings emphasize the importance of vigilant monitoring and timely intervention to prevent and manage hypotensive episodes in this vulnerable population. Further research is warranted to explore strategies for optimizing hemodynamic stability and reducing cardiac complications in elderly SCI patients.
Bard9	Background: Increasing life expectancy has led to an older population with spinal cord injury (SCI). Elderly SCI patients are at increased risk for cardiovascular complications, including hypotension. The purpose of this study was to investigate the frequency of hypotension requiring vasopressor treatment and the associated cardiac complications in elderly SCI patients.<br>Methods: TRACK-SCI is a prospective multi-institutional registry of patients with SCI. We identified 40 elderly patients (≥65 years of age) with traumatic SCI who were enrolled in TRACK-SCI from 2015 to 2019. The primary outcome was the frequency of hypotension requiring vasopressor treatment. Secondary outcomes included cardiac complications, in-hospital mortality, and neurological improvement.<br>Results: Overall, 32 patients (80%) required vasopressor treatment for hypotension. The most common cardiac complications were atrial fibrillation (20%), pneumonia (15%), and deep vein thrombosis (10%). There was one in-hospital death (2.5%). No patients had neurological improvement.<br>Conclusions: Elderly SCI patients are at high risk for hypotension requiring vasopressor treatment and cardiac complications. These findings highlight the need for early identification and treatment of hypotension in elderly SCI patients to prevent cardiac complications and improve outcomes.

Journal of Neurosurgery: Spine 10

Title10	Does disc distraction after cervical total disc arthroplasty impact range of motion and patient-reported outcomes?
Abstract10	OBJECTIVE<br>Total disc arthroplasty (TDA) has been established as a safe and effective alternative to anterior cervical discectomy and fusion for the treatment of cervical spine pathology. However, there remains a paucity of studies in the literature regarding the amount of disc height distraction that can be tolerated, as well as its impact on kinematic and clinical outcomes.<br><br>METHODS<br>Patients who underwent 1- or 2-level cervical TDA with a minimum follow-up of 1 year with lateral flexion/extension and patient-reported outcome measures (PROMs) were included. Middle disc space height was measured on preoperative and 6-week postoperative lateral radiographs to quantify the magnitude of disc space distraction, and patients were grouped into < 2-mm distraction and > 2-mm distraction groups. Radiographic outcomes included operative segment lordosis, segmental range of motion (ROM) on flexion/extension, cervical (C2–7) ROM on flexion/extension, and heterotopic ossification (HO). General health and disease-specific PROMs were compared at the preoperative, 6-week, and final postoperative time points. The independent-samples t-test and chi-square test were used to compare outcomes between groups, while multivariate linear regression was used to adjust for baseline differences.<br><br>RESULTS<br>Fifty patients who underwent cervical TDA at 59 levels were included in the analysis. Distraction < 2 mm was seen at 30 levels (50.85%), while distraction > 2 mm was observed at 29 levels (49.15%). Radiographically, after adjustment for baseline differences, C2–7 ROM was significantly greater in the patients who underwent TDA with < 2-mm disc space distraction at final follow-up (51.35° ± 13.76° vs 39.19° ± 10.52°, p = 0.002), with a trend toward significance in the early postoperative period. There were no significant postoperative differences in segmental lordosis, segmental ROM, or HO grades. After the authors controlled for baseline differences, < 2-mm distraction of the disc space led to significantly greater improvement in visual analog scale (VAS)–neck scores at 6 weeks (−3.68 ± 3.12 vs −2.24 ± 2.70, p = 0.031) and final follow-up (−4.59 ± 2.74 vs −1.70 ± 3.03, p = 0.008).<br><br>CONCLUSIONS<br>Patients with < 2-mm disc height difference had increased C2–7 ROM at final follow-up and significantly greater improvement in neck pain after controlling for baseline differences. Limiting differences in disc space height to < 2 mm affected C2–7 ROM but not segmental ROM, suggesting that less distraction may result in more harmonious kinematics between all cervical levels.
ChatGPT10	Objective: This study aims to investigate the influence of disc distraction on range of motion (ROM) and patient-reported outcomes (PROs) in individuals undergoing cervical total disc arthroplasty (CTDA).<br><br>Methods: A prospective cohort study was conducted, involving 120 patients who underwent CTDA for degenerative cervical disc disease. Patients were divided into two groups based on the application of disc distraction during the surgical procedure. Group A (n=60) received disc distraction, while Group B (n=60) underwent CTDA without distraction. Preoperative and postoperative assessments were conducted to evaluate ROM and PROs, including Neck Disability Index (NDI), Visual Analog Scale (VAS) for neck and arm pain, and SF-36 Health Survey.<br><br>Results: The mean ROM significantly increased in both groups following CTDA. However, when comparing the two groups, Group A exhibited a statistically significant improvement in ROM compared to Group B (p<0.001). In terms of PROs, both groups demonstrated a significant reduction in NDI, VAS neck pain, and VAS arm pain scores after surgery (p<0.05). Nevertheless, there were no significant differences in PROs between the two groups (p>0.05).<br><br>Conclusion: The findings of this study suggest that disc distraction after CTDA positively influences postoperative ROM. However, it does not appear to have a significant impact on PROs, including NDI, VAS neck pain, and VAS arm pain scores. These results highlight the potential benefits of disc distraction in enhancing cervical spine mobility, but further research is needed to assess its impact on functional outcomes and long-term patient satisfaction.
Bard10	Cervical total disc arthroplasty (C-TDA) is a minimally invasive procedure that has been shown to be effective in relieving pain and improving function in patients with cervical spinal stenosis. However, some patients may experience limited range of motion (ROM) after C-TDA. The purpose of this study was to investigate the impact of disc distraction on ROM and patient-reported outcomes after C-TDA.<br><br>A retrospective review was conducted of patients who underwent C-TDA at a single institution between 2010 and 2020. Patients were divided into two groups based on whether or not they underwent disc distraction. ROM was assessed preoperatively, immediately postoperatively, and at 12 months postoperatively. Patient-reported outcomes were assessed using the Neck Disability Index (NDI) and the Visual Analog Scale (VAS) for pain.<br><br>There were 100 patients in the study, 50 in each group. There was no significant difference in ROM between the two groups at any time point. However, patients in the disc distraction group had significantly lower NDI scores and VAS pain scores at 12 months postoperatively (p < 0.05).<br><br>These findings suggest that disc distraction may improve ROM and patient-reported outcomes after C-TDA. However, further studies are needed to confirm these findings and to determine the optimal degree of disc distraction.

Neurosurgery Journal 1

Title1	Predictors of Progression-Free Survival in Patients With Spinal Intramedullary Ependymoma: A Multicenter Retrospective Study by the Neurospinal Society of Japan
Abstract1	BACKGROUND AND OBJECTIVES:<br>Ependymoma is the most common spinal intramedullary tumor. Although clinical outcomes have been described in the literature, most of the reports were based on limited numbers of cases or been confined to institutional experience. The objective of this study was to analyze more detailed characteristics of spinal intramedullary ependymoma (SIE) and provide clinical factors associated with progression-free survival (PFS).<br><br>METHODS:<br>This retrospective observational multicenter study included consecutive patients with SIE in the cervical or thoracic spine treated surgically at a total of 58 institutions between 2009 and 2020. The results of pathological diagnosis at each institute were confirmed, and patients with myxopapillary ependymoma, subependymoma, or unverified histopathology were strictly excluded from this study. Outcome measures included surgical data, surgery-related complications, postoperative systemic adverse events, postoperative adjuvant treatment, postoperative functional condition, and presence of recurrence.<br><br>RESULTS:<br>This study included 324 cases of World Health Organization grade II (96.4%) and 12 cases of World Health Organization grade III (3.6%). Gross total resection (GTR) was achieved in 76.5% of cases. Radiation therapy (RT) was applied after surgery in 16 cases (4.8%), all of which received local RT and 5 of which underwent chemotherapy in combination. Functional outcomes were significantly affected by preoperative neurological symptoms, tumor location, extent of tumor resection, and recurrence. Multivariate regression analysis suggested that limited extent of tumor resection or recurrence resulted in poor functional outcomes. Multiple comparisons among the groups undergoing GTR, subtotal resection and biopsy, or partial resection of the tumor showed that the probability of PFS differed significantly between GTR and other extents of resection.<br><br>CONCLUSION:<br>When GTR can be safely obtained in the surgery for SIE, functional maintenance and longer PFS can be expected.
ChatGPT1	Background: Spinal intramedullary ependymomas are rare neoplasms that pose significant challenges in terms of diagnosis and management. The identification of reliable predictors of progression-free survival (PFS) in these patients is essential for guiding treatment strategies and improving patient outcomes. This multicenter retrospective study aims to investigate potential prognostic factors associated with PFS in patients with spinal intramedullary ependymoma.<br><br>Methods: A retrospective analysis was conducted using data from the Neurospinal Society of Japan's comprehensive database, encompassing patients diagnosed with spinal intramedullary ependymoma between January 2000 and December 2018. Demographic, clinical, radiological, and histopathological variables were collected for each patient. Univariate and multivariate Cox regression analyses were performed to identify independent predictors of PFS. The Kaplan-Meier method was used to estimate survival probabilities, and log-rank tests were conducted to compare survival curves.<br><br>Results: A total of 176 patients (mean age, 42.5 years; male:female ratio, 1.2:1) were included in the analysis. The median follow-up period was 64.2 months. The 5-year PFS rate was 78.4%. Univariate analysis revealed several variables significantly associated with PFS, including tumor location, extent of resection, histological grade, preoperative neurological status, and presence of syringomyelia. Multivariate Cox regression analysis identified extent of resection (hazard ratio [HR]: 0.63, 95% confidence interval [CI]: 0.48-0.82, P = 0.001), histological grade (HR: 1.68, 95% CI: 1.27-2.22, P < 0.001), and preoperative neurological status (HR: 1.42, 95% CI: 1.09-1.85, P = 0.009) as independent predictors of PFS. Kaplan-Meier analysis further demonstrated that patients who underwent gross total resection had significantly better PFS compared to those who underwent subtotal resection or biopsy (P < 0.001).<br><br>Conclusion: This multicenter retrospective study identifies extent of resection, histological grade, and preoperative neurological status as important predictors of PFS in patients with spinal intramedullary ependymoma. The findings underscore the significance of maximal safe surgical resection in optimizing PFS outcomes. These prognostic factors should be considered when formulating treatment strategies and counseling patients with spinal intramedullary ependymoma. Further prospective studies are warranted to validate these findings and explore additional predictors of survival in this rare patient population.
Bard1	Background<br><br>Spinal intramedullary ependymoma (sIME) is a rare tumor that arises from the ependymal cells lining the central canal of the spinal cord. The prognosis for patients with sIME is variable, and there are no established predictors of progression-free survival (PFS).<br><br>Methods<br><br>We conducted a multicenter retrospective study of patients with sIME who were treated at 15 institutions in Japan. Patients were followed for a minimum of 2 years for PFS. Predictors of PFS were analyzed using univariable and multivariable Cox proportional hazards models.<br><br>Results<br><br>A total of 226 patients were included in the study. The median age at diagnosis was 39 years, and 55% of patients were male. The most common tumor location was the thoracic spine (64%). The median PFS was 4.5 years.<br><br>In univariable analysis, the following factors were significantly associated with poor PFS: tumor grade (high grade vs. low grade, hazard ratio [HR] = 2.5, 95% confidence interval [CI] = 1.4-4.5), tumor location (thoracic spine vs. cervical or lumbar spine, HR = 1.7, 95% CI = 1.1-2.6), and extent of resection (subtotal vs. gross total, HR = 2.2, 95% CI = 1.4-3.6). In multivariable analysis, only tumor grade remained a significant predictor of PFS (HR = 2.3, 95% CI = 1.4-3.7).<br><br>Conclusions<br><br>In this study, tumor grade was the only significant predictor of PFS in patients with sIME. Patients with high-grade sIME have a worse prognosis than patients with low-grade sIME. Further studies are needed to identify other factors that may predict PFS in patients with sIME.

Neurosurgery Journal 2

Title2	30 Gy in 4 Stereotactic Body Radiotherapy Fractions for Complex Spinal Metastases: Mature Outcomes Supporting This Novel Regimen
Abstract2	BACKGROUND:<br>We designed a 30 Gy in 4 fractions stereotactic body radiotherapy protocol, as an alternative option to our standard 2-fraction approach, for primarily large volume, multilevel, or previously radiated spinal metastases.<br><br>OBJECTIVE:<br>To report imaging-based outcomes of this novel fractionation scheme.<br><br>METHODS:<br>The institutional database was reviewed to identify all patients who underwent 30 Gy/4 fractions from 2010 to 2021. Primary outcomes were magnetic resonance–based vertebral compression fracture (VCF) and local failure per treated vertebral segment.<br><br>RESULTS:<br>We reviewed 245 treated segments in 116 patients. The median age was 64 years (range, 24-90). The median number of consecutive segments within the treatment volume was 2 (range, 1-6), and the clinical target volume (CTV) was 126.2 cc (range, 10.4-863.5). Fifty-four percent had received at least 1 previous course of radiotherapy, and 31% had previous spine surgery at the treated segment. The baseline Spinal Instability Neoplastic Score was stable, potentially unstable, and unstable for 41.6%, 51.8%, and 6.5% of segments, respectively. The cumulative incidence of local failure was 10.7% (95% CI 7.1-15.2) at 1 year and 16% (95% CI 11.5-21.2) at 2 years. The cumulative incidence of VCF was 7.3% (95% CI 4.4-11.2) at 1 year and 11.2% (95% CI 7.5-15.8) at 2 years. On multivariate analysis, age ≥68 years ($P = .038$), CTV volume ≥72 cc ($P = .021$), and no previous surgery ($P = .021$) predicted an increased risk of VCF. The risk of VCF for CTV volumes <72 cc/≥72 cc was 1.8%/14.6% at 2 years. No case of radiation-induced myelopathy was observed. Five percent of patients developed plexopathy.<br><br>CONCLUSION:<br>30 Gy in 4 fractions was safe and efficacious despite the population being at increased risk of toxicity. The lower risk of VCF in previously stabilized segments highlights the potential for a multimodal treatment approach for complex metastases, especially for those with a CTV volume of ≥72 cc.
ChatGPT2	Background: Complex spinal metastases present a therapeutic challenge due to their aggressive nature and proximity to critical neural structures. Stereotactic body radiotherapy (SBRT) has emerged as an effective treatment modality, offering precise tumor targeting and minimal normal tissue toxicity. This study aims to evaluate the mature outcomes of a novel SBRT regimen delivering 30 Gy in 4 fractions for patients with complex spinal metastases.<br><br>Methods: A retrospective analysis was conducted on a cohort of patients with complex spinal metastases treated between January 2010 and December 2017. All patients underwent SBRT with a prescription dose of 30 Gy delivered in 4 fractions over consecutive days. Clinical outcomes, including pain relief, local control, and overall survival, were assessed. Acute and late toxicities were also evaluated using the Common Terminology Criteria for Adverse Events (CTCAE) version 5.0.<br><br>Results: A total of 78 patients (median age, 62 years; male:female ratio, 1.3:1) were included in the analysis. The median follow-up duration was 27 months. Pain relief was achieved in 85.9% of patients, with a median time to pain relief of 4 weeks. Local control was excellent, with 2-year and 5-year local control rates of 93.7% and 84.1%, respectively. The overall survival rates at 1 year, 2 years, and 5 years were 77.3%, 59.1%, and 27.6%, respectively. Acute toxicities were generally mild and self-limiting, with no grade 4 or 5 toxicities reported. Late toxicities were infrequent, with the most common being grade 1 or 2 neuropathy.<br><br>Conclusion: This retrospective analysis demonstrates that delivering 30 Gy in 4 fractions of SBRT for complex spinal metastases yields favorable clinical outcomes. The treatment achieved significant pain relief, excellent local control, and acceptable toxicity profiles. The results support the feasibility and efficacy of this novel SBRT regimen, providing a promising treatment option for patients with complex spinal metastases. Further prospective studies are warranted to validate these findings and optimize treatment strategies in this patient population.
Bard2	Background: Complex spinal metastases (CSMs) are a challenging clinical problem, and there is no standard of care for their treatment. Stereotactic body radiotherapy (SBRT) has emerged as a promising option for the treatment of CSMs, but the optimal fractionation schedule is unknown.<br><br>Methods: We retrospectively reviewed the medical records of patients with CSMs who received 30 Gy in 4 fractions of SBRT. Patients were followed for a minimum of 12 months for local control (LC), progression-free survival (PFS), and overall survival (OS).<br><br>Results: A total of 50 patients were included in the study. The median age at diagnosis was 65 years, and 68% of patients were male. The most common primary tumor sites were lung (48%), breast (20%), and prostate (14%). The median follow-up time was 24 months. The 1-year and 2-year LC rates were 92% and 84%, respectively. The 1-year and 2-year PFS rates were 76% and 68%, respectively. The 1-year and 2-year OS rates were 90% and 82%, respectively.<br><br>Conclusions: Our results suggest that 30 Gy in 4 fractions of SBRT is an effective and well-tolerated treatment for CSMs. This novel fractionation schedule offers a shorter treatment course and potentially lower toxicity than conventional SBRT schedules.

Neurosurgery Journal 3

Title3	Minimally Invasive Transforaminal Lumbar Interbody Fusion in the Ambulatory Surgery Center Versus Inpatient Setting: A 1-Year Comparative Effectiveness Analysis
Abstract3	BACKGROUND:<br>Ambulatory surgery centers (ASCs) have emerged as an alternative setting for surgical care as part of the national effort to lower health care costs. The literature regarding the safety of minimally invasive transforaminal lumbar interbody fusion (MIS TLIF) in the ASC setting is limited to few small case series.<br><br>OBJECTIVE:<br>To assess the safety and efficacy of MIS TLIF performed in the ASC vs inpatient hospital setting.<br><br>METHODS:<br>A total of 775 patients prospectively enrolled in the Quality Outcomes Database undergoing single-level MIS TLIF at a single ASC (100) or the inpatient hospital setting (675) were compared. Propensity matching generated 200 patients for analysis (100 per cohort). Demographic data, resource utilization, patient-reported outcome measures (PROMs), and patient satisfaction were assessed.<br><br>RESULTS:<br>There were no significant differences regarding baseline demographic data, clinical history, or comorbidities after propensity matching. Only 1 patient required inpatient transfer from the ASC because of intractable pain. All other patients were discharged home within 23 hours of surgery. The rates of 90-day readmission (2.0%) and reoperation (0%) were equivalent between groups. Both groups experienced significant improvements in all PROMs (Oswestry Disability Index, EuroQol-5D, back pain, and leg pain) at 3 months that were maintained at 1 year. PROMs did not differ between groups at any time point. Patient satisfaction was similar between groups at 3 and 12 months after surgery.<br><br>CONCLUSION:<br>In carefully selected patients, MIS TLIF may be performed safely in the ASC setting with no statistically significant difference in safety or efficacy in comparison with the inpatient setting.
ChatGPT3	Background: Minimally invasive transforaminal lumbar interbody fusion (MIS TLIF) has gained popularity as a surgical treatment for degenerative lumbar spine disorders. The choice between performing MIS TLIF in an ambulatory surgery center (ASC) versus an inpatient setting remains a subject of debate. This study aims to compare the effectiveness of MIS TLIF in terms of clinical outcomes and healthcare resource utilization between ASC and inpatient settings over a 1-year follow-up period.<br><br>Methods: A retrospective analysis was conducted on a cohort of patients who underwent MIS TLIF for degenerative lumbar spine disorders between January 2018 and December 2019. Patients were divided into two groups based on the surgical setting: ASC and inpatient. Clinical outcomes, including patient-reported outcomes (PROs), complication rates, length of hospital stay, readmission rates, and revision surgery rates, were evaluated at 1-year follow-up. Healthcare resource utilization, including direct costs and indirect costs, was also assessed.<br><br>Results: A total of 256 patients (ASC: 136; inpatient: 120) were included in the analysis. The ASC group demonstrated comparable improvements in PROs, including pain relief, functional status, and patient satisfaction, compared to the inpatient group at 1-year follow-up ($P > 0.05$). Complication rates were similar between the two groups (ASC: 9.6%; inpatient: 8.3%; $P = 0.745$). The ASC group had significantly shorter hospital stays (1.4 days vs. 4.8 days, $P < 0.001$) and lower readmission rates (3.7% vs. 8.3%, $P = 0.104$) compared to the inpatient group. The revision surgery rates were comparable (ASC: 5.1%; inpatient: 6.7%; $P = 0.618$). The direct costs and indirect costs were significantly lower in the ASC group compared to the inpatient group ($P < 0.001$).<br><br>Conclusion: This comparative effectiveness analysis demonstrates that MIS TLIF performed in an ASC setting yields comparable clinical outcomes and lower healthcare resource utilization compared to the inpatient setting over a 1-year follow-up period. The ASC approach provides similar improvements in PROs, while significantly reducing hospital stays, readmission rates, and healthcare costs. These findings support the feasibility and cost-effectiveness of performing MIS TLIF in an ASC, offering a viable alternative to the traditional inpatient setting. Further prospective studies with larger sample sizes are warranted to validate these results and explore long-term outcomes.
Bard3	Background<br><br>Minimally invasive transforaminal lumbar interbody fusion (MIS TLIF) is a surgical procedure used to treat lumbar spinal stenosis and degenerative disc disease. The procedure can be performed in either the inpatient or ambulatory surgery center (ASC) setting.<br><br>Methods<br><br>We conducted a retrospective cohort study of patients who underwent MIS TLIF at a single academic medical center. Patients were divided into two groups: those who underwent surgery in the ASC (n = 100) and those who underwent surgery in the inpatient setting (n = 675). Patients were followed for a minimum of 1 year for safety and efficacy outcomes.<br><br>Results<br><br>There were no significant differences between the two groups in terms of patient demographics, clinical history, or comorbidities. The rate of complications was 2% in the ASC group and 3% in the inpatient group. The rate of reoperation was 0% in both groups. At 1 year, there were no significant differences between the two groups in terms of patient-reported outcome measures (PROMs) or satisfaction with surgery.<br><br>Conclusions<br><br>MIS TLIF can be safely and effectively performed in the ASC setting. There is no significant difference in safety or efficacy between the ASC and inpatient settings.

Neurosurgery Journal 4

Title4	Daily Pain Prediction Using Smartphone Speech Recordings of Patients With Spine Disease
Abstract4	BACKGROUND:<br>Pain evaluation remains largely subjective in neurosurgical practice, but machine learning provides the potential for objective pain assessment tools.<br><br>OBJECTIVE:<br>To predict daily pain levels using speech recordings from personal smartphones of a cohort of patients with diagnosed neurological spine disease.<br><br>METHODS:<br>Patients with spine disease were enrolled through a general neurosurgical clinic with approval from the institutional ethics committee. At-home pain surveys and speech recordings were administered at regular intervals through the Beiwe smartphone application. Praat audio features were extracted from the speech recordings to be used as input to a K-nearest neighbors (KNN) machine learning model. The pain scores were transformed from a 0 to 10 scale to low and high pain for better discriminative capacity.<br><br>RESULTS:<br>A total of 60 patients were enrolled, and 384 observations were used to train and test the prediction model. Using the KNN prediction model, an accuracy of 71% with a positive predictive value of 0.71 was achieved in classifying pain intensity into high and low. The model showed 0.71 precision for high pain and 0.70 precision for low pain. Recall of high pain was 0.74, and recall of low pain was 0.67. The overall F1 score was 0.73.<br><br>CONCLUSION:<br>Our study uses a KNN to model the relationship between speech features and pain levels collected from personal smartphones of patients with spine disease. The proposed model is a stepping stone for the development of objective pain assessment in neurosurgery clinical practice.
ChatGPT4	Background: Objective and accurate pain assessment is crucial for effective management of patients with spine disease. Smartphone-based technologies offer a promising approach for remote monitoring and prediction of pain levels. This study aims to investigate the feasibility and accuracy of using smartphone speech recordings to predict daily pain levels in patients with spine disease.<br><br>Methods: A prospective study was conducted involving patients diagnosed with spine disease who used a smartphone application to record daily speech samples for a period of 3 months. Pain levels were self-reported by patients on a numerical rating scale (NRS) ranging from 0 to 10. Acoustic features, including pitch, intensity, speech rate, and spectral energy, were extracted from the speech recordings. Machine learning algorithms, including support vector regression and random forest, were employed to build predictive models for daily pain levels based on the acoustic features.<br><br>Results: A total of 80 patients (mean age, 55.6 years; male:female ratio, 1:1.2) participated in the study. The average daily pain level reported by patients was 5.8 ± 1.9 (mean ± standard deviation) on the NRS. The predictive models achieved good accuracy in estimating daily pain levels based on smartphone speech recordings, with a mean absolute error of 1.3 ± 0.7. The most informative acoustic features for pain prediction included pitch variability, intensity modulation, and spectral entropy. The models exhibited superior performance in predicting pain levels during periods of stable pain compared to periods of fluctuating pain.<br><br>Conclusion: This prospective study demonstrates the feasibility and accuracy of using smartphone speech recordings for daily pain prediction in patients with spine disease. The acoustic features extracted from the speech recordings provide valuable insights into pain levels and can be effectively utilized in machine learning algorithms for pain estimation. Smartphone-based pain prediction has the potential to facilitate remote monitoring and personalized pain management in patients with spine disease, enabling timely interventions and improved patient outcomes. Further validation studies and the development of user-friendly smartphone applications are warranted to translate these findings into clinical practice.
Bard4	Background<br><br>Pain is a common and debilitating symptom of spine disease. Current methods for pain assessment are subjective and rely on patient self-report. This can lead to inaccurate pain assessment and suboptimal treatment.<br><br>Objective<br><br>To develop a novel method for predicting daily pain levels using smartphone speech recordings in patients with spine disease.<br><br>Methods<br><br>A total of 60 patients with spine disease were enrolled in this study. Patients completed daily pain surveys and recorded short speech samples using the Beiwe smartphone application. Speech samples were analyzed using Praat to extract acoustic features. A K-nearest neighbors (KNN) machine learning model was trained to predict daily pain levels from the acoustic features.<br><br>Results<br><br>The KNN model was able to predict daily pain levels with an accuracy of 71%. The model was also able to distinguish between high and low pain levels with a positive predictive value of 0.71.<br><br>Conclusion<br><br>This study demonstrates the feasibility of using smartphone speech recordings to predict daily pain levels in patients with spine disease. This approach could be used to improve pain assessment and management in patients with spine disease.

Neurosurgery Journal 5

Title5	Identifying Subsets of Patients With Adult Spinal Deformity Who Maintained a Positive Response to Nonoperative Management
Abstract5	BACKGROUND:<br>Adult spinal deformity (ASD) represents a major cause of disability in the elderly population in the United States. Surgical intervention has been shown to reduce disability and pain in properly indicated patients. However, there is a small subset of patients in whom nonoperative treatment is also able to durably maintain or improve symptoms.<br><br>OBJECTIVE:<br>To examine the factors associated with successful nonoperative management in patients with ASD.<br><br>METHODS:<br>We retrospectively evaluated a cohort of 207 patients with nonoperative ASD, stratified into 3 groups: (1) success, (2) no change, and (3) failure. Success was defined as a gain in minimal clinically importance difference in both Oswestry Disability Index and Scoliosis Research Society-Pain. Logistic regression model and conditional inference decision trees established cutoffs for success according to baseline (BL) frailty and sagittal vertical axis.<br><br>RESULTS:<br>In our cohort, 44.9% of patients experienced successful nonoperative treatment, 22.7% exhibited no change, and 32.4% failed. Successful nonoperative patients at BL were significantly younger, had a lower body mass index, decreased Charlson Comorbidity Index, lower frailty scores, lower rates of hypertension, obesity, depression, and neurological dysfunction (all $P < .05$) and significantly higher rates of grade 0 deformity for all Schwab modifiers (all $P < .05$). Conditional inference decision tree analysis determined that patients with a BL ASD-frailty index ≤ 1.579 (odds ratio: 8.3 [4.0-17.5], $P < .001$) were significantly more likely to achieve nonoperative success.<br><br>CONCLUSION:<br>Success of nonoperative treatment was more frequent among younger patients and those with less severe deformity and frailty at BL, with BL frailty the most important determinant factor. The factors presented here may be useful in informing preoperative discussion and clinical decision-making regarding treatment strategies.
ChatGPT5	Background: Nonoperative management is a common approach for treating patients with adult spinal deformity (ASD) who may not be surgical candidates or prefer conservative treatment. However, there is significant heterogeneity in patient response to nonoperative management. This study aims to identify subsets of patients with ASD who maintained a positive response to nonoperative management and to characterize the factors associated with treatment success.<br><br>Methods: A retrospective analysis was conducted on a cohort of patients with ASD who underwent nonoperative management between January 2010 and December 2019. Patient demographics, radiographic parameters, baseline clinical characteristics, and treatment modalities were assessed. The ODI (Oswestry Disability Index) and VAS (Visual Analog Scale) scores were used to evaluate functional outcomes and pain levels, respectively. Patients were classified into two groups based on treatment response: responders and non-responders. Multivariate logistic regression analysis was performed to identify factors associated with treatment success.<br><br>Results: A total of 187 patients (mean age, 58.3 years; male:female ratio, 1:1.8) were included in the analysis. The overall response rate to nonoperative management was 65.8%. Responders demonstrated significantly lower baseline ODI and VAS scores compared to non-responders ($P < 0.001$). Multivariate logistic regression analysis identified several factors associated with treatment success, including lower baseline ODI score (odds ratio [OR]: 0.78, 95% confidence interval [CI]: 0.68-0.90, $P < 0.001$), lower baseline VAS score (OR: 0.84, 95% CI: 0.74-0.95, $P = 0.005$), and absence of sagittal malalignment (OR: 0.37, 95% CI: 0.18-0.77, $P = 0.008$).<br><br>Conclusion: This retrospective analysis identifies subsets of patients with ASD who maintained a positive response to nonoperative management. Lower baseline disability and pain scores, as well as absence of sagittal malalignment, were associated with treatment success. These findings highlight the importance of patient selection and baseline characteristics in predicting the likelihood of a positive response to nonoperative management in patients with ASD. Tailoring treatment strategies based on these factors may optimize outcomes and improve patient satisfaction. Prospective studies are warranted to validate these results and develop personalized treatment algorithms for nonoperative management of ASD.
Bard5	Background: Adult spinal deformity (ASD) is a complex and increasingly prevalent spinal condition that may lead to substantial disability and pain. Surgical intervention has been shown to reduce disability and pain in properly indicated patients. However, there is a small subset of patients in whom nonoperative treatment is also able to durably maintain or improve symptoms.<br><br>Methods: We retrospectively evaluated a cohort of 207 patients with ASD who were treated nonoperatively. Patients were followed for a mean of 3 years. The primary outcome was the proportion of patients who maintained a positive response to nonoperative management, defined as a gain of ≥5 points on the Oswestry Disability Index (ODI) and ≥10 points on the Scoliosis Research Society-22 (SRS-22) Pain subscale. Secondary outcomes included the proportion of patients who had no or minimal progression of their deformity, as measured by the Cobb angle, and the proportion of patients who were satisfied with their treatment.<br><br>Results: At 3 years, 44.9% of patients maintained a positive response to nonoperative management. Patients who maintained a positive response were younger, had a lower body mass index, had lower levels of frailty, and had less severe deformities at baseline. Patients who maintained a positive response were also more likely to be satisfied with their treatment.<br><br>Conclusions: A subset of patients with ASD can achieve durable pain relief and functional improvement with nonoperative treatment. Patients who are younger, have lower body mass indexes, have lower levels of frailty, and have less severe deformities at baseline are more likely to respond favorably to nonoperative management.

Journal of Bone and Joint Surgery 1

Title1	Neurologic Outcomes After Radiation Therapy for Severe Spinal Cord Compression in Multiple Myeloma: A Study of 162 Patients
Abstract1	Background: Bone destruction is the most frequent disease-defining clinical feature of multiple myeloma (MM), resulting in skeletal-related events such as back pain, pathological fractures, or neurologic compromise including epidural spinal cord compression (ESCC). Up to 24% of patients with MM will be affected by ESCC. Radiation therapy has been proven to be highly effective in pain relief in patients with MM. However, a critical knowledge gap remains with regard to neurologic outcomes in patients with high-grade ESCC treated with radiation.<br>Methods: We retrospectively included 162 patients with MM and high-grade ESCC (grade 2 or 3) who underwent radiation therapy of the spine between January 2010 and July 2021. The primary outcome was the American Spinal Injury Association (ASIA) score after 12 to 24 months, or the last known ASIA score if the patient had had a repeat treatment or died. Multivariable logistic regression was used to assess factors associated with poor neurologic outcomes after radiation, defined as neurologic deterioration or lack of improvement.<br>Results: After radiation therapy, 34 patients (21%) had no improvement in their impaired neurologic function and 27 (17%) deteriorated neurologically. Thirty-six patients (22%) underwent either surgery or repeat irradiation after the initial radiation therapy. There were 100 patients who were neurologically intact at baseline (ASIA score of E), of whom 16 (16%) had neurologic deterioration. Four variables were independently associated with poor neurologic outcomes: baseline ASIA (odds ratio [OR] = 6.50; 95% confidence interval [CI] = 2.70 to 17.38; p < 0.001), Eastern Cooperative Oncology Group (ECOG) performance status (OR = 6.19; 95% CI = 1.49 to 29.49; p = 0.015), number of levels affected by ESCC (OR = 4.02; 95% CI = 1.19 to 14.18; p = 0.026), and receiving steroids prior to radiation (OR = 4.42; 95% CI = 1.41 to 16.10; p = 0.015).<br>Conclusions: Our study showed that 38% of patients deteriorated or did not improve neurologically after radiation therapy for high-grade ESCC. The results highlight the need for multidisciplinary input and efforts in the treatment of high-grade ESCC in patients with MM. Future studies will help to improve patient selection for specific and standardized treatments and to clearly delineate which patients are likely to benefit from radiation therapy.
ChatGPT1	Background: Severe spinal cord compression is a devastating complication of multiple myeloma, requiring prompt intervention to prevent permanent neurologic deficits. This study aimed to evaluate the neurologic outcomes of patients with severe spinal cord compression in multiple myeloma who underwent radiation therapy.<br><br>Methods: A retrospective analysis was conducted on a cohort of 162 patients diagnosed with severe spinal cord compression due to multiple myeloma. All patients received radiation therapy as the primary treatment modality. Neurologic outcomes were assessed using the American Spinal Injury Association (ASIA) impairment scale and the Frankel grade at baseline, immediately post-radiation, and at follow-up intervals. Factors associated with favorable neurologic outcomes were analyzed using logistic regression models.<br><br>Results: Of the 162 patients, 82.7% demonstrated improvement in neurologic function following radiation therapy. The majority of patients experienced an improvement in ASIA impairment scale grades, with 59.9% showing an improvement of one or more grades. Similarly, 65.4% of patients exhibited an improvement in Frankel grade. Notably, 45.1% of patients achieved a Frankel grade of E or D, indicating ambulation with or without assistance. Higher baseline ASIA impairment scale grade and shorter duration of symptoms were associated with more favorable neurologic outcomes (p < 0.001).<br><br>Conclusion: Radiation therapy as the primary treatment for severe spinal cord compression in multiple myeloma yielded favorable neurologic outcomes in the majority of patients. Significant improvements were observed in both the ASIA impairment scale and Frankel grade, indicating enhanced neurologic function and ambulatory capabilities. Early intervention and prompt initiation of radiation therapy were associated with better neurologic outcomes. These findings underscore the importance of early diagnosis and timely intervention to optimize neurologic recovery in patients with severe spinal cord compression in multiple myeloma.
Bard1	Background: Radiation therapy is a standard treatment for severe spinal cord compression (SCC) in multiple myeloma (MM). However, the long-term neurologic outcomes after radiation therapy for SCC in MM are not well known.<br><br>Methods: We conducted a retrospective study of 162 patients with MM who received radiation therapy for SCC. The primary outcome was the proportion of patients who had no or mild neurologic deficits at 12 months after radiation therapy. Secondary outcomes included the proportion of patients who had no or mild neurologic deficits at 24 months and 5 years after radiation therapy, and the proportion of patients who had a good neurologic outcome (defined as no or mild neurologic deficits and no need for surgery or other interventions) at 12 months, 24 months, and 5 years after radiation therapy.<br><br>Results: At 12 months after radiation therapy, 88% of patients had no or mild neurologic deficits. At 24 months, 82% of patients had no or mild neurologic deficits. At 5 years, 74% of patients had no or mild neurologic deficits. The proportion of patients who had a good neurologic outcome at 12 months, 24 months, and 5 years was 92%, 88%, and 78%, respectively.<br><br>Conclusions: Radiation therapy is an effective treatment for SCC in MM with good long-term neurologic outcomes. The majority of patients who receive radiation therapy for SCC in MM will have no or mild neurologic deficits at 12 months, 24 months, and 5 years after radiation therapy.

Journal of Bone and Joint Surgery 2

Title2	Uncovertebral Joint Fusion Versus End Plate Space Fusion in Anterior Cervical Spine Surgery: A Prospective Randomized Controlled Trial
Abstract2	Background: The uncovertebral joint is a potential region for anterior cervical fusion. Currently, we are aware of no clinical trials on human uncovertebral joint fusion (UJF). The purpose of this study was to compare the time it took to achieve osseous union/fusion and the clinical efficacy of UJF to end plate space fusion (ESF)—i.e., traditional anterior cervical discectomy and fusion (ACDF)—in anterior cervical surgery.<br>Methods: Patients with single-level cervical spondylosis were recruited from April 2021 through October 2022 and randomly divided into the UJF and ESF groups, with 40 patients in each group. Autologous iliac bone was used for bone grafting in both groups. The primary outcome was the early fusion rate at 3 months postoperatively. Secondary outcomes included the prevalence of complications and patient-reported outcome measures (PROMs), including the Japanese Orthopaedic Association (JOA) score, Neck Disability Index (NDI), and visual analog scale (VAS) scores for arm and neck pain.<br>Results: A total of 74 patients (92.5%) with an average age of 49.8 years (range, 26 to 65 years) completed the trial and were included in the analysis. There was no significant difference between the 2 groups at baseline. The operative duration and intraoperative blood loss were also comparable between the 2 groups. The fusion rate in the UJF group was significantly higher than that in the ESF group at 3 months (66.7% compared with 13.2%, $p < 0.0001$) and 6 months (94.1% compared with 66.7%, $p = 0.006$) after the operation. No significant difference was found in the fusion rate between the 2 groups 12 months postoperatively. Overall, the PROMs significantly improved after surgery in both groups and did not differ significantly between the groups at any follow-up time point. The prevalence of complications was not significantly different between the 2 groups.<br>Conclusions: In our study of anterior cervical fusion surgery, we found that the early fusion rate after UJF was significantly higher than that after ESF.
ChatGPT2	Background: Anterior cervical spine surgery is commonly performed to treat symptomatic cervical degenerative disc disease. The choice of fusion technique, such as uncovertebral joint fusion (UJF) or end plate space fusion (EPSF), remains a matter of debate. This prospective randomized controlled trial aimed to compare the clinical efficacy and radiographic fusion rates between UJF and EPSF techniques.<br><br>Methods: A total of 120 patients with symptomatic cervical degenerative disc disease were enrolled and randomly assigned to either the UJF or EPSF group. Clinical outcomes were assessed using validated measures, including the visual analog scale (VAS) for neck and arm pain, Neck Disability Index (NDI), and modified Japanese Orthopedic Association (mJOA) score. Radiographic fusion rates were evaluated based on dynamic X-rays and computed tomography (CT) scans.<br><br>Results: Both UJF and EPSF groups demonstrated significant improvements in clinical outcomes compared to baseline values. In terms of pain reduction, the UJF group exhibited slightly superior outcomes in VAS scores for both neck and arm pain ($p < 0.05$). However, no significant differences were observed between the groups in terms of NDI and mJOA scores. Radiographic assessment revealed a higher fusion rate in the UJF group (93.3%) compared to the EPSF group (87.5%) at the 12-month follow-up, although the difference was not statistically significant.<br><br>Conclusions: This prospective randomized controlled trial demonstrates that both UJF and EPSF techniques are effective in improving clinical outcomes in anterior cervical spine surgery. The UJF technique may provide slight advantages in terms of pain reduction, while showing a trend towards a higher radiographic fusion rate. These findings contribute valuable insights into the selection of fusion techniques for patients with cervical degenerative disc disease, allowing surgeons to make informed decisions based on individual patient characteristics and preferences.
Bard2	Background: Uncovertebral joint (UJ) fusion is a common surgical technique used to treat anterior cervical spine disorders. End plate space fusion (EPSF) is a newer technique that involves fusing the vertebral bodies without fusing the UJs.<br><br>Methods: We conducted a prospective randomized controlled trial to compare the clinical and radiographic outcomes of UJ fusion and EPSF in patients with anterior cervical spine disorders. A total of 100 patients were randomly assigned to receive either UJ fusion ($n = 50$) or EPSF ($n = 50$). The primary outcome was clinical success, defined as the absence of any of the following at 24 months: neck pain, myelopathy, radiculopathy, and instability. The secondary outcomes were radiographic fusion rate, adjacent segment disease, and complications.<br><br>Results: At 24 months, the clinical success rates were 94% in the UJ fusion group and 88% in the EPSF group. The radiographic fusion rates were 98% in the UJ fusion group and 96% in the EPSF group. There was no significant difference in the rates of adjacent segment disease or complications between the two groups.<br><br>Conclusions: UJ fusion and EPSF are both effective surgical techniques for treating anterior cervical spine disorders. There was no significant difference in clinical or radiographic outcomes between the two groups.
Reference2	

Journal of Bone and Joint Surgery 3

Title3	Long-Term Radiographic and Pulmonary Function Outcomes After Dual Growing-Rod Treatment for Severe Early-Onset Scoliosis
Abstract3	Background: We evaluated long-term outcomes (radiographic parameters and pulmonary function) at a minimum follow-up of 5 years after use of dual growing rods (DGRs) to treat severe early-onset scoliosis (sEOS).<br>Methods: Among a total of 112 patients who were diagnosed with early-onset scoliosis (EOS) and were treated with DGRs between 2006 and 2015, 52 patients had sEOS, with a major Cobb angle of >80°. Of these patients, 39 with a minimum follow-up of 5 years had complete radiographic and pulmonary function test results and were included. The Cobb angle of the major curve, T1-S1 height, T1-T12 height, and maximum kyphosis angle in the sagittal plane were measured on radiographs. Pulmonary function test results were collected in all patients before the initial operation (preoperatively), 12 months after the initial operation (postoperatively), and at the last follow-up. The changes in pulmonary function and complications during treatment were analyzed.<br>Results: The mean age of patients before the initial operation was 7.7 ± 1.2 years, and the mean follow-up period was 75.0 ± 14.1 months. The mean number of lengthenings was 4.5 ± 1.3, and the mean interval between lengthenings was 11.2 ± 2.1 months. The Cobb angle improved from 104.5° ± 18.2° preoperatively to 38.1° ± 10.1° after the initial surgical procedure (postoperatively) and 21.9° ± 8.6° at the final follow-up. The T1-S1 height increased from 25.1 ± 4.0 cm preoperatively to 32.4 ± 3.5 cm postoperatively and to 39.5 ± 4.0 cm at the final follow-up. However, no significant difference was detected between the increased pulmonary function parameters at 1 year and those before the operation ($p > 0.05$), except for residual volume, whereas pulmonary function parameters had significantly increased at the final follow-up ($p < 0.05$). During the treatment period, 17 complications occurred in 12 patients.<br>Conclusions: DGRs are effective in treating sEOS in the long term. They allow longitudinal growth of the spine, and the correction of the spinal deformity can provide conditions that make improving pulmonary function possible in patients with sEOS.
ChatGPT3	Background:<br>Severe early-onset scoliosis (EOS) poses significant challenges in terms of spinal deformity progression and potential pulmonary compromise. Dual growing-rod treatment has emerged as a promising intervention for managing EOS. This study aimed to evaluate the long-term radiographic and pulmonary function outcomes following dual growing-rod treatment in patients with severe EOS.<br><br>Methods:<br>A retrospective analysis was conducted on a cohort of 50 patients with severe EOS who underwent dual growing-rod treatment. Radiographic parameters, including Cobb angle, T1-S1 height, and thoracic kyphosis, were measured preoperatively and at regular follow-up intervals. Pulmonary function tests (PFTs) were performed to assess the impact of the treatment on respiratory function. Complications and reoperations were also documented.<br><br>Results:<br>The study included 50 patients with severe EOS who underwent dual growing-rod treatment, with a mean follow-up duration of 5 years. Significant improvements were observed in radiographic parameters over time, with a mean decrease in the Cobb angle of 30 degrees ($p < 0.001$) and a mean increase in T1-S1 height of 25 mm ($p < 0.001$) at the latest follow-up. Thoracic kyphosis remained stable throughout the follow-up period ($p = 0.112$). Pulmonary function outcomes demonstrated a favorable trend, with a mean increase in forced vital capacity (FVC) of 15% predicted ($p = 0.003$) and a mean increase in forced expiratory volume in 1 second (FEV1) of 12% predicted ($p = 0.008$) at the latest follow-up. The complication rate was 20%, with the most common complication being rod breakage (15%). Reoperation was required in 10% of patients, mainly due to implant-related issues or curve progression.<br><br>Conclusion:<br>Dual growing-rod treatment for severe EOS demonstrated favorable long-term radiographic and pulmonary function outcomes. The treatment resulted in significant improvements in spinal alignment, with stable thoracic kyphosis and substantial increases in T1-S1 height. Moreover, the intervention showed positive effects on respiratory function, as evidenced by improvements in FVC and FEV1. Although complications and reoperations were observed, the benefits of dual growing-rod treatment outweighed the risks, making it a valuable option for managing severe EOS. Further studies are warranted to evaluate the impact of these outcomes on patients' quality of life and to compare dual growing-rod treatment with other surgical interventions for severe EOS.
Bard3	Background: Early-onset scoliosis (EOS) is a rare and progressive spinal deformity that can occur in children under the age of 10. Severe EOS can lead to significant deformity, pain, and respiratory compromise. Dual growing rods (DGRs) are a surgical treatment option for EOS that allows for longitudinal growth of the spine while maintaining correction of the deformity.<br><br>Methods: We evaluated long-term radiographic and pulmonary function outcomes in patients with severe EOS who were treated with DGRs. A total of 39 patients with a mean age of 7.7 years at the time of surgery were included in the study. The mean follow-up period was 75.0 months. Radiographic parameters, including the Cobb angle, T1-S1 height, and T1-T12 height, were evaluated at baseline, 1 year, and final follow-up. Pulmonary function tests were performed at baseline, 1 year, and final follow-up.<br><br>Results: At final follow-up, the mean Cobb angle had decreased from 104.5° to 21.9°. The mean T1-S1 height had increased from 25.1 cm to 39.5 cm. There were no significant changes in pulmonary function parameters at 1 year, but there were significant improvements in pulmonary function parameters at final follow-up.<br><br>Conclusions: DGRs are an effective treatment option for severe EOS in the long term. They allow for longitudinal growth of the spine, and the correction of the spinal deformity can provide conditions that make improving pulmonary function possible in patients with severe EOS.

Journal of Bone and Joint Surgery 4

Title4	Identification of Novel Genetic Markers for the Risk of Spinal Pathologies: A Genome-Wide Association Study of 2 Biobanks
Abstract4	Background: Identifying genetic risk factors for spinal disorders may lead to knowledge regarding underlying molecular mechanisms and the development of new treatments.<br>Methods: Cases of lumbar spondylolisthesis, spinal stenosis, degenerative disc disease, and pseudarthrosis after spinal fusion were identified from the UK Biobank. Controls were patients without the diagnosis. Whole-genome regressions were used to test for genetic variants potentially implicated in the occurrence of each phenotype. External validation was performed in FinnGen.<br>Results: A total of 389,413 participants were identified from the UK Biobank. A locus on chromosome 2 spanning GFPT1, NFU1, AAK1, and LOC124906020 was implicated in lumbar spondylolisthesis. Two loci on chromosomes 2 and 12 spanning genes GFPT1, NFU1, and PDE3A were implicated in spinal stenosis. Three loci on chromosomes 6, 10, and 15 spanning genes CHST3, LOC102723493, and SMAD3 were implicated in degenerative disc disease. Finally, 2 novel loci on chromosomes 5 and 9, with the latter corresponding to the LOC105376270 gene, were implicated in pseudarthrosis. Some of these variants associated with spinal stenosis and degenerative disc disease were also replicated in FinnGen.<br>Conclusions: This study revealed nucleotide variations in select genetic loci that were potentially implicated in 4 different spinal pathologies, providing potential insights into the pathological mechanisms.
ChatGPT4	Background:<br>Spinal pathologies, including spinal stenosis, disc degeneration, and intervertebral disc herniation, are multifactorial disorders influenced by both genetic and environmental factors. Understanding the genetic basis of these conditions is crucial for early detection, risk assessment, and potential targeted interventions. This study aimed to identify novel genetic markers associated with the risk of spinal pathologies through a comprehensive genome-wide association study (GWAS) using data from two large biobanks.<br><br>Methods:<br>A GWAS was conducted using genetic data from two biobanks, comprising a total of 50,000 individuals. Cases with diagnosed spinal pathologies and controls without any evidence of spinal pathologies were included. Single nucleotide polymorphism (SNP) genotyping and imputation were performed using state-of-the-art genotyping arrays and reference panels. Genome-wide association analysis was conducted to identify genetic variants associated with the risk of spinal pathologies. Statistical significance was determined using stringent thresholds, and Bonferroni correction was applied to account for multiple testing.<br><br>Results:<br>Several novel genetic markers were identified as significantly associated with the risk of spinal pathologies. Notably, SNP rs123456 in gene ABC showed a strong association with the risk of disc degeneration (p = 2.35 × 10^-8), corresponding to an odds ratio (OR) of 1.78 (95% confidence interval [CI]: 1.52-2.09). Similarly, SNP rs789012 in gene XYZ was significantly associated with the risk of intervertebral disc herniation (p = 1.12 × 10^-6), with an OR of 1.63 (95% CI: 1.32-2.01). These associations remained statistically significant after adjusting for relevant covariates.<br><br>Conclusion:<br>This genome-wide association study identified novel genetic markers associated with the risk of spinal pathologies, providing valuable insights into the genetic underpinnings of these conditions. The identified genetic variants, such as SNP rs123456 in gene ABC for disc degeneration and SNP rs789012 in gene XYZ for intervertebral disc herniation, are located in genes involved in crucial biological processes associated with spine development and disc homeostasis. These findings contribute to our understanding of the complex etiology of spinal pathologies and may have implications for personalized risk assessment, early detection, and potential therapeutic interventions. Further functional studies are warranted to elucidate the precise mechanisms through which these genetic variants influence disease susceptibility.
Bard4	Background<br><br>Spinal pathologies are a common cause of pain and disability. The risk of developing spinal pathologies is influenced by both genetic and environmental factors.<br><br>Methods<br><br>We conducted a genome-wide association study (GWAS) to identify novel genetic markers for the risk of spinal pathologies. We included participants from two large biobanks: the UK Biobank and the FinnGen Biobank. We genotyped participants for over 1 million genetic variants and used a statistical method called logistic regression to test for associations between genetic variants and the risk of spinal pathologies.<br><br>Results<br><br>We identified five novel genetic markers for the risk of lumbar spondylolisthesis, three novel genetic markers for the risk of spinal stenosis, and two novel genetic markers for the risk of degenerative disc disease. The genetic variants were associated with a small increase in the risk of spinal pathologies.<br><br>Conclusions<br><br>Our findings suggest that genetic factors play a role in the risk of developing spinal pathologies. The identification of novel genetic markers for spinal pathologies could lead to the development of new preventive strategies and treatments.

Journal of Bone and Joint Surgery 5

Title5	Accuracy Evaluation of a Novel Spinal Robotic System for Autonomous Laminectomy in Thoracic and Lumbar Vertebrae: A Cadaveric Study
Abstract5	Background: The main function of robots in spine surgery is to assist with pedicle screw placement. Laminectomy, which is as important as pedicle screw placement, lacks a mature robot-assisted system. The aims of this study were to introduce the first autonomous laminectomy robot, to explore the feasibility of autonomous robotic laminectomy, and to validate its accuracy using a cadaveric model.<br>Methods: Forty vertebrae from 4 cadavers were included in the study; 7 thoracic and 3 lumbar vertebrae were randomly selected in each cadaver. The surgeon was able to plan the laminectomy path based on computed tomographic (CT) data before the surgical procedure. The robot performed the laminectomy autonomously, and a postoperative CT scan was made. The deviation of each cutting plane from the plan was quantitatively analyzed, and the accuracy and safety were qualitatively evaluated. The time required for the laminectomy was also recorded.<br>Results: Cuts were performed in 80 laminectomy planes (56 for thoracic vertebrae and 24 for lumbar vertebrae). The mean time for 1-sided laminectomy was 333.59 ± 116.49 seconds, which was shorter for thoracic vertebrae (284.41 ± 66.04 seconds) than lumbar vertebrae (448.33 ± 128.65 seconds) ($p < 0.001$). The mean time for single-level total laminectomy was 814.05 ± 302.23 seconds, which was also shorter for thoracic vertebrae (690.46 ± 165.74 seconds) than lumbar vertebrae (1,102.42 ± 356.13 seconds) ($p = 0.002$). The mean deviation of the cutting plane from the plan was 0.67 ± 0.30 mm for the most superior cutting point and 0.73 ± 0.31 mm for the most inferior point. There were no significant differences in the deviation between thoracic vertebrae (0.66 ± 0.26 mm) and lumbar vertebrae (0.67 ± 0.38 mm) at the superior cutting point ($p = 0.908$) and between thoracic vertebrae (0.72 ± 0.30 mm) and lumbar vertebrae (0.73 ± 0.33 mm) at the inferior cutting point ($p = 0.923$). In the qualitative analysis of the accuracy of the 80 laminectomy planes, 66 (83%) were classified as grade A, 14 (18%) were grade B, and none was grade C. In the safety analysis, 65 planes (81%) were considered safe and the safety of the other 15 planes (19%) was considered uncertain.<br>Conclusions: The results confirmed the accuracy of this robotic system, supporting its use for laminectomy of thoracolumbar vertebrae.
ChatGPT5	Background:<br>Spinal laminectomy is a common surgical procedure performed to alleviate symptoms associated with spinal stenosis and other spinal pathologies. With recent advancements in robotic technology, the development of autonomous robotic systems for spinal surgery holds promise in improving surgical precision and patient outcomes. This study aimed to evaluate the accuracy and performance of a novel spinal robotic system designed for autonomous laminectomy in thoracic and lumbar vertebrae.<br><br>Methods:<br>A cadaveric study was conducted on ten human cadavers to evaluate the accuracy and feasibility of the novel spinal robotic system. The robotic system utilized preoperative computed tomography (CT) scans for preoperative planning and real-time intraoperative feedback. The system consisted of a robotic arm, a surgical tool holder, and a custom-built navigation platform. Various laminectomy procedures were performed autonomously by the robotic system under the supervision of an experienced surgeon. Accuracy measurements were obtained by comparing the planned resection areas with the actual resection areas in the cadavers.<br><br>Results:<br>The novel spinal robotic system demonstrated a high level of accuracy in performing autonomous laminectomy procedures. The mean deviation between the planned and actual resection areas was found to be within acceptable limits, with an average discrepancy of 1.2 mm (±0.4 mm) in thoracic vertebrae and 1.4 mm (±0.6 mm) in lumbar vertebrae. The system consistently achieved precise resection with minimal damage to adjacent structures, as confirmed by postoperative CT scans and dissection of the cadaveric specimens.<br><br>Conclusion:<br>The findings of this cadaveric study suggest that the novel spinal robotic system for autonomous laminectomy in thoracic and lumbar vertebrae is accurate, reliable, and feasible. The system demonstrates the potential to enhance surgical precision, reduce surgeon fatigue, and minimize complications associated with conventional manual laminectomy procedures. Further clinical studies are warranted to evaluate the system's performance in live surgical settings and its impact on patient outcomes. With continued refinement and integration, autonomous robotic systems hold promise in advancing the field of spinal surgery and improving patient care.
Bard5	Background<br><br>Laminectomy is a common surgical procedure used to relieve pressure on the spinal cord or nerves. It is often performed in conjunction with other procedures, such as spinal fusion or discectomy. The accuracy of laminectomy is critical to the success of the surgery.<br><br>Methods<br><br>A novel spinal robotic system for autonomous laminectomy was developed. The system uses a CT scan of the spine to create a three-dimensional model of the vertebrae. The surgeon can then plan the laminectomy on the model and the robot will autonomously execute the plan.<br><br>A cadaveric study was performed to evaluate the accuracy of the robotic system. Forty vertebrae from four cadavers were included in the study. The robot performed laminectomies on 7 thoracic and 3 lumbar vertebrae in each cadaver. The accuracy of the laminectomies was evaluated by comparing the postoperative CT scans to the preoperative plans.<br><br>Results<br><br>The robot was able to perform laminectomies with high accuracy. The mean deviation of the cuts from the planned paths was 0.5 mm. There were no cases of nerve injury or other complications.<br><br>Conclusions<br><br>The results of this study suggest that the robotic system is a safe and accurate tool for laminectomy. The system has the potential to improve the accuracy and reproducibility of laminectomy, which could lead to better outcomes for patients.
